# Genetic and epigenetic determinants of reactivation of Mecp2 and the inactive X chromosome in neural stem cells

**DOI:** 10.1016/j.stemcr.2022.01.008

**Published:** 2022-02-10

**Authors:** H. Mira-Bontenbal, B. Tan, C. Gontan, S. Goossens, R.G. Boers, J.B. Boers, C. Dupont, M.E. van Royen, W.F.J. IJcken, P. French, A. Bedalov, J. Gribnau

**Affiliations:** 1 Oncode Institue, Department of Developmental Biology, Erasmus MC, Rotterdam, the Netherlands; 2 Department of Pathology, Erasmus MC, Rotterdam, the Netherlands; 3 Center for Biomics, Department of Cell Biology, Erasmus MC, Rotterdam, the Netherlands; 4 Department of Neurology, Erasmus MC Cancer Institute, University Medical Center, Rotterdam, the Netherlands; 5 Clinical Research Division, Fred Hutchinson Cancer Research Center, 1100 Fairview Avenue N., Seattle, WA 98109, USA

**Keywords:** Mecp2, neural stem cells, X chromosome reactivation, Xist, 5-azacitidine, ASO, Rett Syndrome

## Abstract

Rett syndrome may be treated by reactivating the silent copy of *Mecp2* from the inactive X chromosome in female cells. Most studies that model *Mecp2* reactivation have used mouse fibroblasts rather than neural cells, which would be critical for phenotypic reversal, and rely on fluorescent reporters that lack adequate sensitivity. Here, we present a mouse model based on a dual bioluminescent and fluorescent reporter to assess the level of reactivation of *Mecp2* and the inactive X chromosome by treating neural stem cells with 5-azacytidine and *Xist* knockdown. We show that reactivation of *Mecp2* and other X-linked genes correlates with CpG density, with distance from escapees, and, very strongly, with the presence of short interspersed nuclear elements. In addition, X-linked genes reactivated in neural stem cells overlap substantially with early reactivating genes by induced pluripotent stem cell reprogramming of fibroblasts or neuronal progenitors, indicating that X chromosome reactivation follows similar paths regardless of the technique or cell type used.

## Highlights

• Xist knockdown and 5-Aza treatment lead to Mecp2 reactivation in neural stem cells
• · SINE density and gene activity are potent indicators of X-linked gene reactivation
• Genes reactivated in different cell types by different means overlap considerably

## Teaser

In this article, Hegias Mira-Bontenbal, Joost Gribnau, and colleagues show that SINEs and activity signatures on the active X chromosome are strong predictors of X-linked gene reactivation from the inactive X in mouse neural stem cells. Different mechanisms to reactivate the X lead to a similar pool of reactivated genes in different cell types, implying a general path to reactivation.

## Introduction

Rett syndrome (RTT) is the second most prevalent cause of intellectual disability in girls after Down syndrome, affecting 1 in 10,000 live female births (Weaving et al., 2005). It is caused by heterozygous mutations in the methyl-CpG-binding protein 2 (MECP2), whose gene is X linked and subject to random X chromosome inactivation (XCI) during early embryogenesis. RTT-affected girls are thus mosaic in terms of *MECP2* expression: half of their cells will express the wild-type (WT) copy of *MECP2*, while the other half will express the mutant *MECP2* allele. This also implies that RTT-affected cells have a silenced WT *MECP2* copy located on the inactive X chromosome (Xi). Previous work has shown that postnatal re-expression of WT *Mecp2* copies in an RTT mouse model causes its phenotype to revert (Giacometti et al., 2007; Guy et al., 2007), which has sparked major interest in the RTT field in re-expressing WT MECP2 in human RTT patients. One way of achieving this is by reactivation of the endogenous WT copy of *MECP2* on the Xi in RTT cells.

In mice, XCI is initiated early in pre-implantation development, where at the eight-cell stage the paternally inherited X is inactivated (Okamoto et al., 2004). Subsequently, the inactive X is reactivated in the inner cell mass (ICM), followed by random XCI of either the maternally or the paternally inherited X. From there on, the inactive state is inherited by all daughter cells, and only in the developing oocyte is the inactive X reactivated (Mak et al., 2004). Hence, cells in the ICM of the female mouse blastocyst and female embryonic stem cells (ESCs) bear two active X chromosomes. Upon development and epiblast formation or ESC differentiation, one of the X chromosomes is randomly chosen to upregulate expression of the long non-coding RNA *Xist* (Monkhorst et al., 2008). This results in the coating of a single X chromosome with *Xist* and recruitment of proteins such as SPEN, RBM15, HDAC3, and the polycomb repressive complexes PRC1 and PRC2 to silence X-linked genes in *cis* (Chu et al., 2015; Fang et al., 2004; McHugh et al., 2015; Minajigi et al., 2015; Moindrot et al., 2015; Monfort et al., 2015; Napoles et al., 2004; Plath et al., 2004). Eventually, CpGs at promoters become methylated to lock XCI down (Gendrel et al., 2013).

Several studies have delved into the mechanics of *Mecp2* reactivation or, in more general terms, X chromosome reactivation (XCR) in mouse cells and tissues by looking for factors that are important in maintaining *Xist* expression, by directly knocking down *Xist*, or by inhibiting the DNA methyltransferase DNMT1 (Adrianse et al., 2018; Bhatnagar et al., 2014; Carrette et al., 2018; Lessing et al., 2016; Przanowski et al., 2018; Sripathy et al., 2017). The combination of *Xist* knockdown using short hairpin RNA (shRNA) or antisense oligonucleotides (ASOs) with 5-azacytidine (5-Aza; a DNMT1 inhibitor) treatment synergistically reactivated *Mecp2* fused to a firefly luciferase reporter on the Xi of a mouse fibroblast cell line (Carrette et al., 2018; Sripathy et al., 2017). In addition, blocking the PI3K/AKT/mTor pathway using inhibition of SGK1, a downstream effector of PDPK1, or mTOR with GSK650394 or rapamycin, respectively, resulted in biallelic expression of *Mecp2* in mouse fibroblasts, while inhibition of ACVR1 with LDN193189 led to similar results (Przanowski et al., 2018). Treatment of fibroblasts carrying a GFP transgene on the Xi with rapamycin, GSK650394, or LDN193189 led to increased fluorescence (Przanowski et al., 2018), confirming that the PI3K/AKT/mTOR and BMP pathways are involved in maintenance of repression of the Xi. *In vivo*, injection of GSK650394 and LDN193189 into brains of *Xist*^−/+^:*Mecp2*^+/GFP^ mice where *Mecp2* is fused to GFP on the Xi resulted as well in significant GFP expression (Przanowski et al., 2018). Additional studies also showed that inhibition of DNMT1 and Aurora kinases results in synergistic reactivation of an Xi-linked GFP transgene (Lessing et al., 2016). A more suitable approach to performing high-throughput chemical compound screens for *Mecp2* reactivation requires the generation of an improved mouse model, the derivation of its associated cell lines closer to the neuronal target cells, and the use of a highly sensitive luciferase, instead of fluorescence, whose expression is under the control of the endogenous *Mecp2* promoter and not a transgene on the X chromosome.

Here, we have developed a mouse model system where *Mecp2* is fused to NLuc, a luciferase enzyme smaller and 100 times brighter than the regular firefly luciferase. We have also introduced a fluorescent TdTomato reporter downstream of NLuc separated by a P2A signal. This dual capability permits not only measurement of NLuc activity at a populational level, but also measurement of Tomato fluorescence at the single-cell level. Our *Xist*^−/+^:*Mecp2*^+/*NLucTom*^ compound mice display complete skewed XCI of the reporter allele and are generated in a highly polymorphic C57BL/6:Cast/Eij (maternal:paternal) F1 hybrid background, providing a wealth of SNPs for X-chromosome-wide allele-specific expression analysis. From these mutant mice, we have isolated mouse embryonic fibroblasts (MEFs), ESCs, and neural stem cells (NSCs) for further studies and to provide them to the community. We show that 5-Aza treatment in combination with *Xist* knockdown in NSCs leads to XCR with a striking resemblance to induced pluripotent stem cell (iPSC)-reprogramming-specific XCR (Janiszewski et al., 2019; Bauer et al., 2021), suggesting a general pattern in the capability of X-linked genes to reactivate independent of the mechanism or cell type. In this article, we highlight the potential of our model to study XCR.

## Results

### Generation of *Mecp2*-*NanoLuciferase-TdTomato* mice

**Figure 1:**
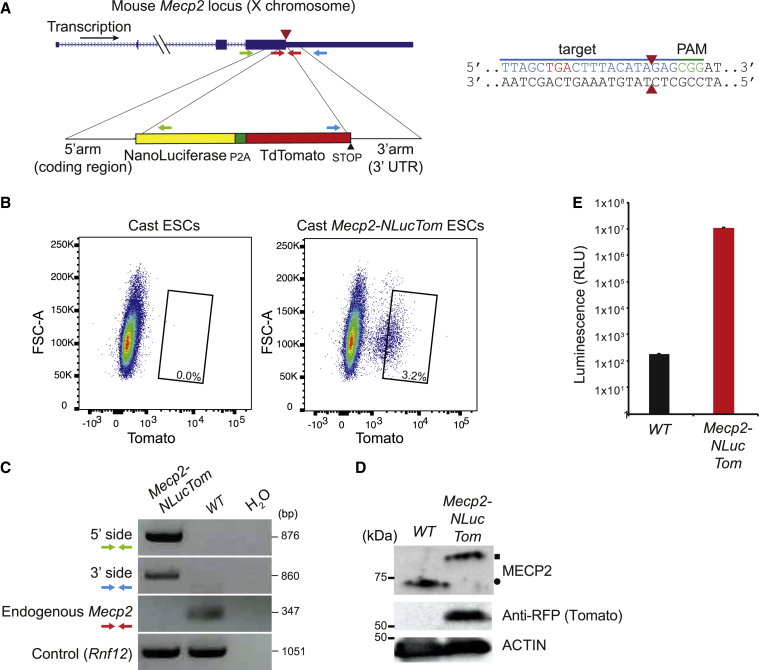
*Mecp2*^*NLucTom/Y*^ male cast ESCs show proper reporter integration and expression (A) Mouse *Mecp2* locus with the NLuc and Tomato donor vector. Green, blue, and red primer sets were used to amplify the 5′ integration site (forward primer outside the 5′ homology arm, inside the coding region, and reverse primer inside NLuc), the 3′ integration site (forward primer in Tomato and reverse primer outside of 3′ homology arm), and the non-targeted endogenous end of *Mecp2*, respectively (see [C]). The guide RNA, PAM, cutting site (red arrowheads), and *Mecp2*'s TGA STOP codon are depicted on the right. Primer sequences are found in Table S1. (B) FACS plots depicting Tomato fluorescence before and after transfection of WT male cast ESCs with CRISPR-Cas9 and the donor vector depicted in (A). The rectangle shows the sorted population, 3.2% of the total live population. (C) Genomic PCR with primers described in (A) on FACS-sorted *Mecp2*^*NLucTom/Y*^ cast ESCs and parental WT ESCs. A control locus PCR band is depicted (*Rnf12*). (D) Western blot analysis of FACS-sorted *Mecp2*^*NLucTom/Y*^ ESCs and parental WT ESCs. Tomato is translated as an independent protein, thanks to the P2A signal. Loading control, actin. MECP2-NLuc and WT MECP2 are indicated by a square and a circle, respectively. (E) NLuc activity assay of FACS-sorted *Mecp2*^*NLucTom/Y*^ ESCs and parental WT ESCs (500,000 cells analyzed per well, average activity ± SD, n = 3 independent biological replicates).

To obtain highly polymorphic *Xist*^−/+^:*Mecp2*^+/*NLucTom*^ mice, we first generated *Mecp2*^*NLucTom/Y*^ ESCs in a Cast/EiJ (cast) background. We transfected WT male cast ESCs with the *NanoLuciferase-P2A-TdTomato* (NLucTom) construct, where NLuc is fused to the C terminus of *Mecp2* and TdTomato (Tomato from here on) is translated as an independent protein, thanks to a P2A self-cleaving peptide (Figure 1A). Fluorescence-activated cell sorting (FACS) analysis showed a distinct Tomato-positive cell population that was sorted and expanded (Figure 1B). PCR analysis using primers spanning the 5′- and 3′-specific integration sites and primers against the endogenous allele confirmed proper integration on DNA obtained from sorted Tomato-positive cells (Figures 1A and 1C, Table S1). This resulted in the appearance of a higher-molecular-weight band of MECP2 by immunoblotting owing to its fusion to NLuc (19 kDa, Figure 1D). Luminescence analysis showed very strong NLuc activity in *Mecp2*^*NLucTom/Y*^ ESCs compared with WT ESCs (Figure 1E). Cells were then injected into blastocysts and a cast colony of *Mepc2-NLucTom* mice was generated. *Mecp2*^*NLucTom/Y*^ mice are viable with normal lifespan and do not show any RTT-related phenotype, indicating that the fusion of NLuc to MECP2 is not deleterious to its function (Figure S1A). Immunofluorescence (IF) for NLuc and Tomato fluorescence analysis in a *Mecp2*^*+/LucTom*^ fully cast female brain shows that MECP2-NLuc and Tomato are expressed in 45% of the cells, as expected from random XCI (Figure S1B). Moreover, *Mecp2*^+/*LucTom*^ female brains also show high NLuc activity compared with WT controls, highlighting the usefulness of this system for *in vivo* studies (Figure S1C). We have thus generated a *Mecp2-NLucTom* mouse colony in a cast background.

### Generation of *Xist*^−/+^:*Mecp2*^+/*NLucTom*^, *Xist*^−/+^:*Mecp2*^−/*NLucTom*^, *Mecp2*^+/*NLucTom*^, and *Mecp2*^*NLucTom/NLucTom*^ cell lines

**Figure 2:**
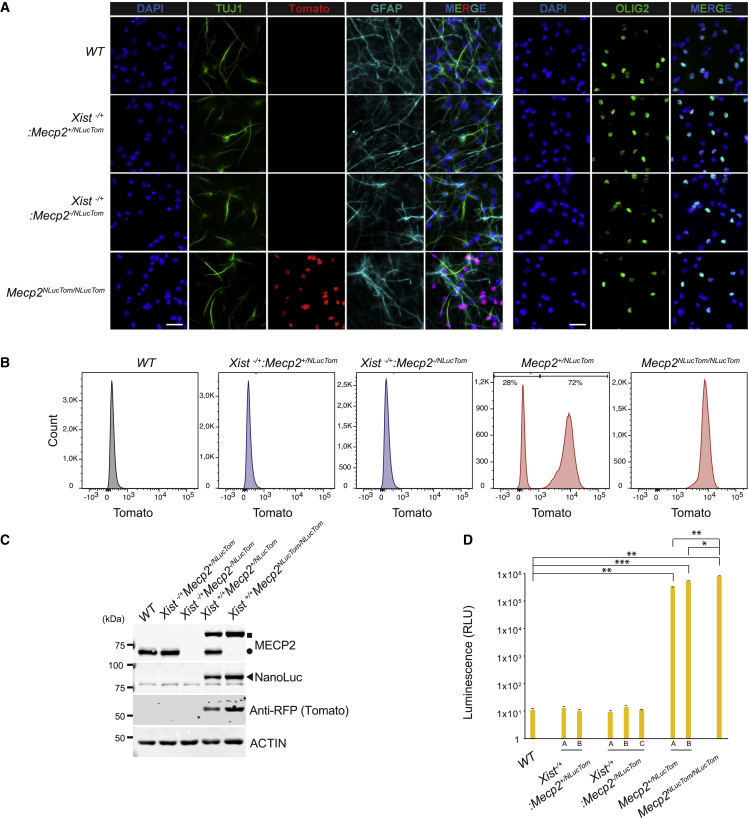
Characterization of *Xist*^−/+^:*Mecp2*^+/*NLucTom*^, *Xist*^−/+^:*Mecp2*^−/*NLucTom*^, *Mecp2*^+/*NLucTom*^, and *Mecp2*^*NLucTom/NLucTom*^ NSCs (A) IF of TUJ1 (green, left), GFAP (turquoise), and OLIG2 (green, right) of WT, *Xist*^−/+^:*Mecp2*^+/*NLucTom*^, *Xist*^−/+^:*Mecp2*^−/*NlucTom*^, and *Mecp2*^*NLucTom/NLucTom*^ NSCs differentiated toward neurons, astrocytes, and oligodendrocytes. Tomato fluorescence was measured directly. Blue, DAPI. White scale bars, 25 μm; n = 1. (B) FACS analysis of Tomato fluorescence of WT, *Xist*^−/+^:*Mecp2*^+/*NLucTom*^, *Xist*^−/+^:*Mecp2*^−/*NLucTom*^, and *Mecp2*^*NLucTom/NlucTom*^ NSCs. The percentage of Tomato-positive *Mecp2*^+/*NlucTom*^ NSCs is shown. (C) Western blot analysis of WT, *Xist*^−/+^:*Mecp2*^+/*NlucTom*^, *Xist*^−/+^:*Mecp2*^−/*NlucTom*^, and *Mecp2*^*NLucTom/NLucTom*^ NSCs showing expression of MECP2-NLuc in *Mecp2*^+/*NLucTom*^ and *Mecp2*^*NLucTom/NLucTom*^ NSCs but not in *Xist*^−/+^:*Mecp2*^+/*NLucTom*^, *Xist*^−/+^:*Mecp2*^−/*NLucTom*^ NSCs as expected. MECP2-NLuc, WT MECP2, and NLuc are indicated by a square, a circle, and a triangle, respectively. Tomato is expressed as an independent protein. (D) NLuc activity assay of several clones of WT, *Xist*^−/+^:*Mecp2*^+/*NlucTom*^, *Xist*^−/+^:*Mecp2*^−/*NlucTom*^, *Mecp2*^+/*NlucTom*^, and *Mecp2*^*NLucTom/NLucTom*^ NSCs, showing an increase of four to five levels of magnitude of NLuc activity from an active X chromosome in *Mecp2*^+/*NLucTom*^ and *Mecp2*^*NLucTom/NLucTom*^ NSCs (50,000 cells were analyzed per clone per well, average activity ± SD, n = 3 independent biological replicates). ^∗^p < 0.05, ^∗∗^p < 0.01, ^∗∗∗^p < 0.001, two-tailed Student’s t test.

To study *Mecp2* reactivation, we crossed cast *Mecp2*^*NLucTom/Y*^ males with C57BL/6 (Bl6) WT, *Zp3-Cre:Xist*^+/*2lox*^, or *Zp3-Cre:Xist*^+/*2lox*^*:Mecp2*^+/*2lox*^ females (Figure S2A). Oocyte-specific expression of Cre, thanks to the *Zp3-Cre* transgene, results in recombination of *loxP* sites before fertilization, resulting in female embryos that are *Xist*^−/+^:*Mecp2*^+/*NLucTom*^ and *Xist*^−/+^:*Mecp2*^−/*NLucTom*^ (among other genotypes). In this way, we isolated Bl6:cast WT control, *Xist*^−/+^:*Mecp2*^+/*NLucTom*^, *Xist*^−/+^:*Mecp2*^−/*NLucTom*^, *Mecp2*^+/*NLucTom*^, and cast *Mecp2*^*NLucTom/NLucTom*^ MEF, ESC, and NSC lines. Genotyping of the F1 NSCs confirmed proper integration of the NLuc-Tomato cassette at the *Mecp2* locus in mice (Figure S2B). We confirmed by IF SOX2 expression and absence of the differentiated neuron-specific marker TUJ-1 in our NSC lines (Figure S2C). Our NSC lines were also able to differentiate into TUJ-1-expressing neurons, GFAP-expressing astrocytes, and OLIG2-expressing oligodendrocytes, confirming their stemness (Figure 2A). However, *in vitro* grown neurons with RTT (*Xist*^−/+^:*Mecp2*^−/*NLucTom*^) did not show any differences compared with WT or *Xist*^−/+^:*Mecp2*^+/*NLucTom*^ neurons in terms of nuclear size, number of roots and extremities per nucleus, or total neurite length per nucleus (Figure S2D).

Full skewing of XCI of the paternal cast allele in *Xist*^−/+^:*Mecp2*^+/*NLucTom*^ and *Xist*^−/+^:*Mecp2*^−/*NLucTom*^ NSCs was confirmed by FACS analysis (Figure 2B). In addition, *Mecp2*^+/*NLucTom*^ NSCs displayed skewed XCI as expected from their hybrid origin, where around 60%–70% of the cells were reported to show inactivation of the Bl6 allele (Cattanach and Williams, 1972). *Mecp2*^*NLucTom/NLucTom*^ NSCs displayed a single Tomato-positive peak. Completely skewed XCI in *Xist*^−/+^:*Mecp2*^+/*NLucTom*^ and *Xist*^−/+^:*Mecp2*^−/*NLucTom*^ NSCs and absence of *Mecp2* expression in *Xist*^−/+^:*Mecp2*^−/*NLucTom*^ NSCs were also demonstrated by immunoblotting analysis (Figure 2C). *Mecp2*^+/*NLucTom*^ and *Mecp2*^*NLucTom/NLucTom*^ NSCs showed a higher-molecular-weight band for MECP2-NLuc fusion protein and Tomato expression. Moreover, NLuc activity analysis showed that several *Mecp2*^+/*NLucTom*^ and *Mecp2*^*NLucTom/NLucTom*^ NSC clones had high levels of NLuc activity, and as expected, several *Xist*^−/+^:*Mecp2*^+/*NLucTom*^ and *Xist*^−/+^:*Mecp2*^−/*NLucTom*^ NSC clones did not (Figure 2D). The background levels of NLuc expression in *Xist*^−/+^:*Mecp2*^+/*NLucTom*^ and *Xist*^−/+^:*Mecp2*^−/*NLucTom*^ NSCs were identical to WT cells, indicating that escape of *Mecp2-NLuc* from the inactive cast X chromosome is virtually non-existent from *in vivo*-derived NSCs. To quantify the level of transcriptional repression of *Mecp2-NLuc-Tom* on the Xi, we compared the NLuc activity in cells with the reporter on the active and inactive X. The reporter exhibited a >30,000 times lower level of activity on the Xi compared with the active X chromosome (Figure 2D). Finally, the average NLuc activity arising from two hybrid heterozygous *Mecp2*^+/*NLucTom*^ clones represented 41% and 65% of the activity of the homozygous *Mecp2*^*NLucTom/NLucTom*^ clone.

### Reactivation of the inactive *Mecp2*-NLuciferase allele

Compounds LDN193189 and GSK650394, which inhibit ACVR1 and SGK1, respectively, have been shown to reactivate an inactive GFP reporter on the Xi in fibroblasts and an inactive *Mecp2-GFP* fusion gene in mouse brains (Przanowski et al., 2018). In addition, the HDAC1/3 inhibitor RG2833 has been shown to facilitate XCR during reprogramming of female Xi-linked GFP transgenic MEFs (Janiszewski et al., 2019).

**Figure 3:**
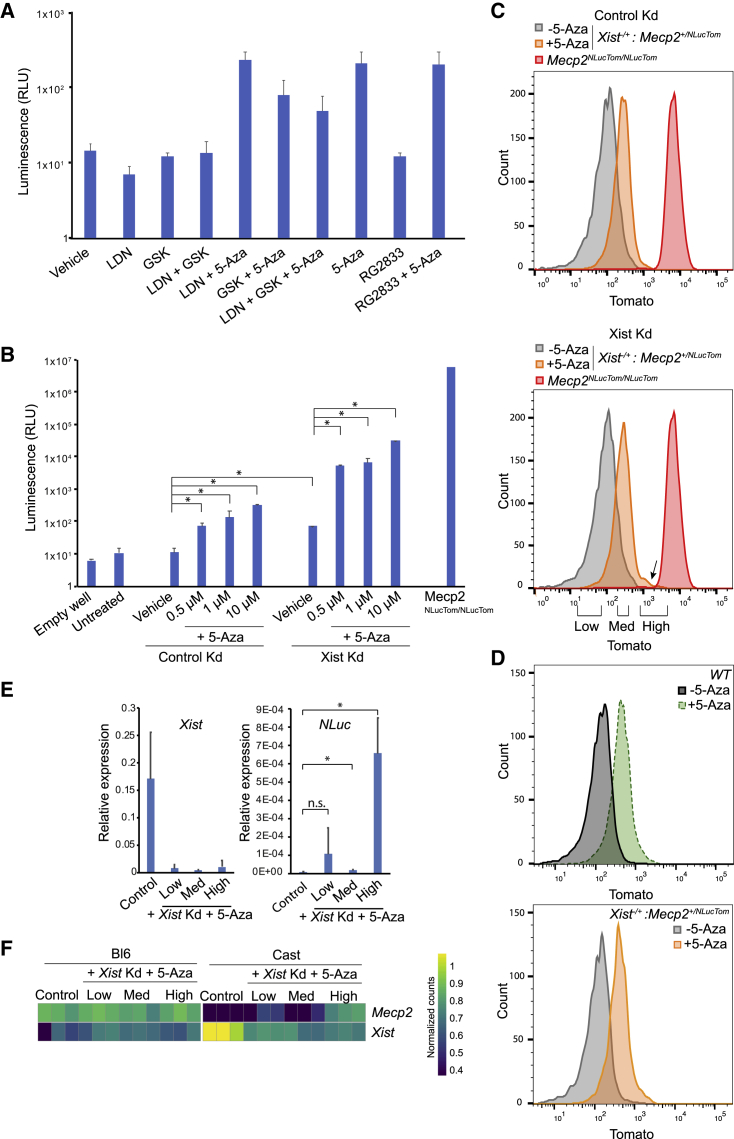
*Xist* knockdown and 5-Aza treatment of *Xist*^−/+^:*Mecp2*^+/*NLucTom*^ NSCs leads to reactivation of the NLuc-Tomato dual reporter (A) NLuc activity assay of *Xist*^−/+^:*Mecp2*^*+/NLucTom*^ NSCs treated with LDN193189 (LDN), GSK650394 (GSK), RG2833, or 5-Aza in different combinations for 7 days (300,000 cells per well, average activity ± SD, n = 3 independent biological replicates). (B) NLuc activity assay of *Xist*^−/+^:*Mecp2*^+/*NLucTom*^ NSCs treated with different concentrations of 5-Aza in combination with control ASOs or *Xist* ASOs for 3 days (average activity ± SD, n = 3 independent biological replicates). Significant differences are indicated with an asterisk (500,000 cells were analyzed per well, two-tailed Student’s t test, ∗p < 0.05). (C) FACS plots depicting *Xist*^−/+^:*Mecp2*^+/*NLucTom*^ NSCs treated with control or *Xist* ASOs with (orange) or without (gray) 5-Aza for 3 days. *Mecp2*^*NLucTom/NLucTom*^ NSCs are shown as Tomato-positive controls (red). FACS-sorted populations that were subsequently analyzed by RNA-seq are shown (Low, Med, High). The shoulder in the *Xist* K_d_ and 5-Aza-treated sample is shown by an arrow; this corresponds to the Tomato-High population; n = 1. (D) FACS plots depicting WT and *Xist*^−/+^:*Mecp2*^+/*NLucTom*^ NSCs treated with (dotted green and orange lines, respectively) or without (black and gray, respectively) 5-Aza for 3 days; n = 1. (E) Relative *Xist* and *NLuc* expression by qRT-PCR analysis in FACS-sorted Tomato-Low, -Med, and -High *Xist*^−/+^:*Mecp2*^+/*NLucTom*^ NSCs after knockdown of *Xist* and 5-Aza treatment versus non-sorted control cells (average activity ± SD, n = 3 independent biological replicates). (F) Expression heatmap of *Mecp2* and *Xist* across the different samples and alleles; n = 3 independent biological replicates.

We therefore treated our NSCs with LDN193189, GSK650394, RG2833, and/or decitabine (structurally very similar to 5-Aza, and called 5-Aza henceforth) for 7 days. Single treatments with LDN193189, GSK650394, or RG2833 and combined treatment with LDN193189 or GSK650394 did not result in *Mecp2* reactivation, however (Figure 3A). Combined treatment of LDN193189 or RG2833 with 5-Aza showed reactivation of the silent NLuc reporter, comparable to single treatment with 5-Aza, indicating that in our hands, 5-Aza is the only tested drug that reactivates the silent copy of *Mecp2*.

Previous work has also shown that 5-Aza treatment in combination with *Xist* knockdown results in XCR in MEFs (Carrette et al., 2018). Therefore, we performed a similar analysis on our *Xist*^−/+^:*Mecp2*^+/*NLucTom*^ NSCs. Treatment of cells with 0.5 μM 5-Aza for 3 days resulted in a significant 10-fold upregulation of NLuc activity (Figure 3B). If *Xist* was knocked down with ASOs in combination with larger amounts of 5-Aza, reactivation was synergistic and 100-fold higher compared with *Xist* knockdown only, and much higher compared with the background of untreated cells (Figures 3B andS3A). Nevertheless, this reactivation still represented around 0.5%–1% of *Mecp2-NLuc* expression from an active X chromosome in homozygous *Mecp2*^*NLucTom/NLucTom*^ NSCs.

**Figure 4:**
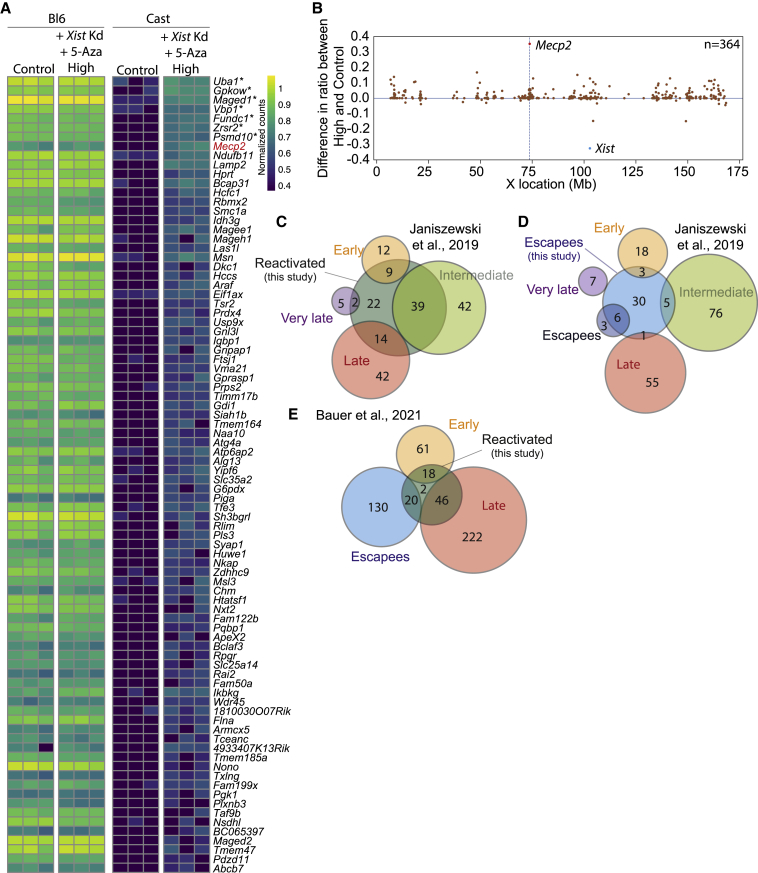
Reactivation of many X-linked genes after *Xist* knockdown and 5-Aza treatment (A) Heatmap showing the normalized allele-specific counts for the 86 genes that are significantly reactivated from the cast Xi in the FACS-sorted Tomato-High population compared with the negative control sample. *Mecp2* is in red. The seven genes more easily reactivated than *Mecp2* bear an asterisk. n = 3 independent biological RNA replicates that were sequenced for all conditions. (B) Difference between the ratios of cast expression to total expression (Bl6 + cast) of Tomato-High and control per gene along the X chromosome. Only genes with sufficient allele-specific reads in both conditions are shown. (C) Venn diagram of the overlap between the reactivated gene list in this study and the early, intermediate, late, or very late reactivated gene subclasses during iPSC reprogramming of MEFs (Janiszewski et al., 2019). (D) Venn diagram of the overlap between the escapees in our study and the early, intermediate, late, very late, and escape gene subclasses during iPSC reprogramming of MEFs (Janiszewski et al., 2019). (E) Venn diagram of the overlap of the reactivated gene list in this study and the early and late reactivating genes during iPSC reprogramming of NPCs (Bauer et al., 2021). Note that 38/86 (44%) of our reactivated genes are either escapees or early reactivating genes in Bauer et al. (2021).

While NLuc bioluminescence analysis is performed at a populational level, flow cytometry allows us to distinguish Tomato fluorescence at the single-cell level. FACS analysis showed that the entire population of cells shifts toward increased Tomato expression after 10 μM 5-Aza treatment for 3 days (Figure 3C), irrespective of whether *Xist* is knocked down or not. This disagrees with the fact that *Xist* knockdown and 10 μM 5-Aza-treated cells show a 100-fold increase in NLuc activity compared with 10 μM 5-Aza-only-treated cells (Figure 3B), suggesting that NLuc bioluminescence is more sensitive than fluorescence. However, control experiments indicated that the shift of the entire population after 5-Aza treatment toward higher Tomato is due to autofluorescence, since WT female cells equally treated with 5-Aza also show indistinguishable increased Tomato fluorescence (Figure 3D). We noticed, however, a small shoulder on the Tomato-High side of the *Xist* ASO-plus-5-Aza-treated population (Figure 3C). We proceeded to use FACS to sort three independent biological replicates of the Tomato-Low, -Med, and -High populations of *Xist*^−/+^:*Mecp2*^+/*NLucTom*^ mNSCs treated with *Xist* ASOs and 5-Aza and subsequently performed RNA sequencing (RNA-seq), along with control ASO and non-5-Aza-treated non-FACS-sorted cells (control). We first confirmed by qRT-PCR proper knockdown of *Xist* in all three Tomato populations and significant upregulation of *NLuc* in the Tomato-Med and -High populations (Figure 3E). Among the 2,612 genes on the X chromosome, we obtained sufficient allelic expression information from 447 active genes, of which 45 were classified as escapees, such as previously described *Mid1*, *Eif2s3x*, *Kdm5c*, and *Ddx3x* (Figure S3B and S3C; Yang, 2010; Berletch, 2015). As expected, *Xist* was expressed from the cast Xi and had decreased expression after its knockdown (Figures 3E and 3F). Allele-specific differential expression analysis showed that 86 genes became reactivated from the cast Xi in the Tomato-High population upon *Xist* knockdown and 5-Aza treatment, *Mecp2* included (Figures 3F and 4A). Reactivated genes were seemingly located in a random fashion along the X chromosome, although several clusters were observed (Figure 4B). Among these 86 genes, 7 were more significantly reactivated than *Mecp2* (Figure 4A, Table S2). In addition, *Mecp2* reactivation in the Tomato-Low and -Med populations was not significant by RNA-seq analysis, as expected from the FACS analysis indicating these populations reflect autofluorescence. However, most of the genes within the 86-gene pool in the Tomato-High population were readily reactivated in the Tomato-Low and -Med populations (in black, Figure S3D, Table S2), while several genes were significantly reactivated only in the Tomato-High population, as was *Mecp2* (in orange, Figure S3D, Table S2). This again suggests that reactivation by *Xist* knockdown and a DNMT1 inhibitor happens more readily for other genes than for *Mecp2*.

In a previous study of iPSC reprogramming of female MEFs, the authors describe different X-linked gene subclasses based on their XCR kinetics, namely early, intermediate, late, and very late reactivation (Janiszewski et al., 2019). We compared our pool of reactivated genes with theirs and observed that 9 and 39 of our genes were among the 21 early (43%) and 81 intermediate (48%) reactivated iPSC genes, respectively (Figure 4C). Similarly, 14/56 (25%) and 2/7 (29%) of our genes were found in their different late and very late reactivation gene subclasses, respectively. This means that a small number of our reactivated genes (22/86, 26%) were not reactivated or were not expressed in the iPSC study. Of note, 6 of their 9 escapees are among our escapee gene pool (67%), while only 9 of their 165 reactivated genes (5%) were in our escapee gene list (Figure 4D), suggesting that our escapee genes are not spuriously reactivated genes, owing to culture conditions, for instance.

XCR kinetics during iPSC reprogramming has also been recently studied in neuronal progenitor cells (NPCs) that were generated through differentiation of ESCs (Bauer et al., 2021). We split their list of reactivated genes into early and late reactivating genes and noticed that 18/79 (23%) and 46/268 (17%) of our genes were found in their early reactivating and late reactivating gene lists, respectively (Figure 4E). Interestingly, we noticed that 20 escapees of their 150-escapee list are genes that were reactivated in our study. This means that 38/86 (44%) of our reactivated genes are either escapees or early reactivating genes in the NPC reprogramming study.

### Genomic and epigenomic features of X chromosome reactivation

**Figure 5:**
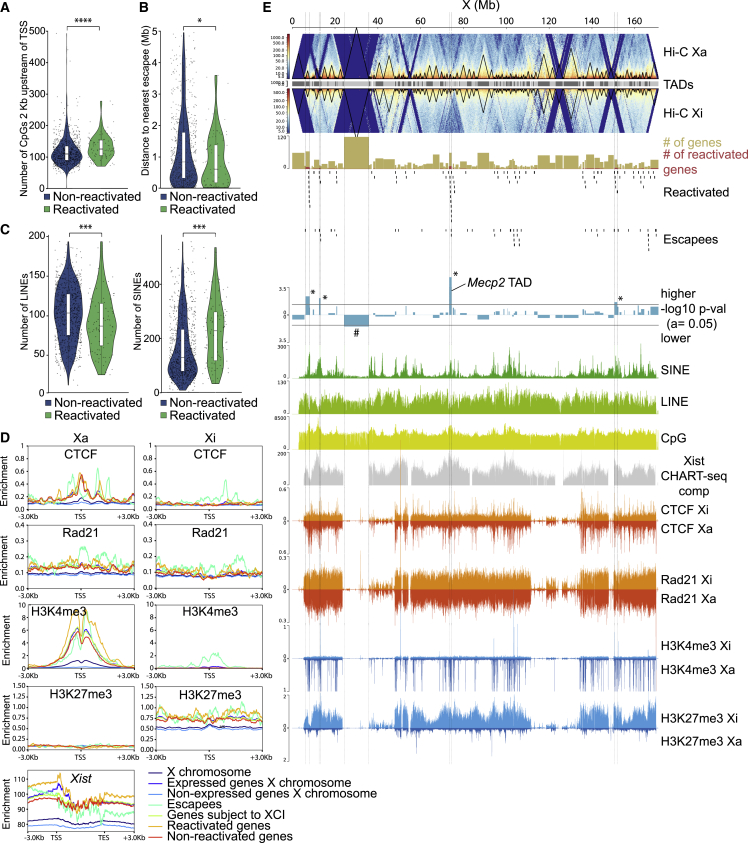
Reactivation of many X-linked genes correlates with genomic and epigenomic features (A) Violin plot depicting the number of CpGs in a bin of 2 kb upstream of the TSS of non-reactivated (blue) and reactivated (green) genes.^∗∗∗∗^p < 0.0001, Mann-Whitney test. (B) Violin plots of the distance to the nearest escapee in megabases of non-reactivated (blue) and reactivated genes (green). ^∗^p < 0.05, Mann-Whitney test (C) Violin plots of the number of LINEs and SINEs in a window of ±100 kb around the TSSs of non-reactivated genes (blue) and reactivated genes (green). ^∗∗∗^p < 0.001, Mann-Whitney test. (D) Average density plots of CTCF and Rad21 binding to, and H3K4me3 and H3K27me3 deposition at, the TSS ± 3 kb of the different gene subclasses on the Xa and Xi, and CHART-seq composite *Xist* enrichment at gene bodies (TSS-TES [transcription end site]) ± 3 kb. *Xist* was removed from the escapee list here in order not to bias the escapee analysis. (E) Genome browser overview showing several genetic and epigenetic features from female NPCs, split into Xa- and Xi-specific signals. Female NPC Hi-C data from Bauer et al. (2021) are shown. For each TAD, the number of overlapping reactivated genes and escapees is identified, as plotted here along the X chromosome. The beige and red rectangles show the number of genes and reactivated genes per TAD, respectively. The −log10 of the p value of a binomial test between the ratio of reactivated genes/total genes per TAD and for the whole X chromosome is shown in blue for each TAD. TADs with a higher or lower ratio than average are plotted in inverse directions. A p value of 0.05 is indicated by a dotted line, and TADs with significantly higher or lower ratios are indicated by ^∗^ or #, respectively. SINE, LINE, CpG, *Xist* (CHART-seq), CTCF, Rad21, H3K4me3, and H3K27me3 densities along the X chromosome are depicted in different colors. Data for CTCF, Rad21, H3K4me3, and H3K27me3 are split into Xa- and Xi-specific densities.

Since our NSCs were subject to 5-Aza treatment, we investigated whether gene reactivation is dependent on CpG-methylation loss. We first analyzed the density of CpGs in the reactivated and non-reactivated subclasses and found that reactivated genes have significantly more CpGs near their transcription start site (TSS) than non-reactivated genes, but did not show differences in CpG density in their gene bodies (Figures 5A and S4A). We subsequently performed methylated DNA sequencing (MeD-seq) analysis (Boers et al., 2018) on the control and Tomato-High populations to assess the methylation status on the Xi. We additionally analyzed male WT NSCs to assess the methylation status of CpGs on the Xa and in this way be able to infer CpG methylation on the Xi of our female cells. We observed a global decrease in methylation on the X chromosome as expected from the 5-Aza treatment (Figure S4B). However, we surprisingly could not detect a correlation between loss of CpG methylation and reactivation of genes on the Xi (Figure S4B). While male NSCs showed low levels of promoter DNA methylation, as expected from expressed genes on the Xa, reactivated promoters in female cells were not significantly demethylated overall in the Tomato-High population compared with control NSCs, although methylation seemed lower for some of them (Figures S4B and S4C, Table S3). Indeed, 16 of our 86 promoters of reactivated genes showed significantly lower levels of DNA methylation (Figure S4C, Table S3). Nevertheless, cluster analysis of the CpG methylation status of promoters of both reactivated and non-reactivated genes did not result in clustering of reactivated promoters (Figure S4D). Altogether, loss of methylation was not a clear indicator of X-linked gene reactivation, pointing to other mechanisms at play.

We subsequently performed genomic feature correlation analyses on our list of reactivated genes. First, we did not detect a correlation between the position of reactivated genes on the X and proximity to *Xist*, as previously described for X-linked reactivated genes during iPSC reprogramming (Janiszewski et al., 2019) (Figure S5A). However, genes that are reactivated tend to be closer to escapees than non-reactivated genes (Figure 5B), suggesting that proximity to an escapee is a determining factor in the reactivation potential of X-linked genes, as previously described (Bauer et al., 2021; Loda et al., 2017). In addition, we find that genes that are more easily reactivated tend to have significantly fewer long interspersed nuclear elements (LINEs) and more short interspersed nuclear elements (SINEs) around their TSSs (Figure 5C). We found no relationship between specific SINE subclasses and reactivated genes (Figure S5B). We then organized LINEs near genes by evolutionary age (Figure S5C) (Sookdeo et al., 2013) and size (full-length LINEs of ≥6 kb or shorter LINEs) (Figure S5D). Age or size of LINEs in nearby genes is not a predictor of gene reactivation capacity. Finally, we found that DNA flanking SINEs and LINEs close to non-reactivated and reactivated genes shows no difference in methylation levels (Figure S5E). MeD-seq analysis, which is based on 32-bp restriction fragments, prevents direct DNA methylation analysis of LINEs and SINEs owing to their repetitive nature.

We next investigated the correlation between our different gene subclasses with published CTCF and Rad21 binding profiles and several chromatin marks from chromatin immunoprecipitation sequencing (ChIP-seq) datasets obtained from ESC-derived female neural progenitor cells (Wang et al., 2018). Escapees tend to show increased enrichment of CTCF at their TSSs (Figure 5D), as has been previously described (Bonev et al., 2017; Loda et al., 2017). In addition, our reactivated genes tend to have slightly more CTCF binding at their TSSs on the Xa compared with non-reactivated genes, while also bearing increased H3K4me3 deposition on the Xa and very similar H3K27me3 levels on the Xi (Figure 5D). Finally, we also measured the density of *Xist* molecules using a published capture hybridization analysis of RNA targets (CHART)-seq dataset from NPCs (Wang et al., 2018) at promoters of X-linked genes and saw more enrichment of *Xist* at promoters of reactivated genes compared with non-reactivated genes (Figure 5D), in line with what has been previously published for active compartments on the X chromosome (Bauer et al., 2021).

We also examined whether certain topologically associating domains (TADs) are more easily reactivated or prevented from reactivation than others by crossing our gene subclasses with previously published TAD data from NPCs (Bonev et al., 2017). Based on the number of reactivated genes within each TAD, we identified four TADs with significantly more reactivated genes compared with the whole X chromosome (as indicated by an asterisk) (Figure 5E). The TAD containing *Mecp2* shows the significantly largest ratio of reactivated genes, likely because the RNA-seq analysis was performed on Tomato-High (*Mecp2*-reactivated) sorted cells, and suggests that co-activation of nearby genes is limited to the same TAD. Indeed, 9 of the 22 genes that were reactivated in this study, but not by iPSC reprogramming of MEFs (Figure 4C), are located within the *Mecp2* TAD (*Plxnb3*, *Idh3g*, *Naa10*, *Hcfc1*, *Mecp2*, *Flna*, *Gdi1*, *Fam50a*, and *Ikbkg*). Interestingly, these four TADs, significantly enriched with reactivated genes, contain a small number of escapees compared with other non-significant TADs (Figure 5E). In contrast, one TAD is particularly resistant to reactivation (as indicated by #) and yet contains many genes. We subsequently investigated whether the presence of CpGs, SINEs, LINEs, CTCF, Rad21, *Xist*, H3K4me3, and H3K27me3 within those five TADs could be an indicator of their tendency or resistance to reactivate. TADs with significantly more reactivated genes than other TADs tended to have more CpGs and fewer LINEs (although not significant) and contained significantly more SINEs (Figures 5E and S5F). Finally, they also significantly tended to have more CTCF binding and H3K4me3 deposition on the Xa than non-significant TADs and to display more *Xist* accumulation and H3K27me3 deposition, although not significantly, than TADs that are not enriched or depleted for reactivated genes (Figures 5E and S5G). It seems that the active status of genes on the Xa correlates clearly with the reactivation potential of genes.

## Discussion

A proper mouse model to study reactivation of *Mecp2* from the Xi in a very sensitive manner has been lacking. We provide here a new mouse model where *Mecp2* has been fused with the bioluminescent reporter NLuc, which is 100 times brighter than the frequently used firefly luciferase, and a fluorescent reporter, Tomato. This dual capability permits not only measurement of NLuc activity at a populational level, but also measurement of Tomato fluorescence at the single-cell level and *in vivo*. *Mecp2*^*NLucTom*^ mice are viable and have been created in a Cast/EiJ background that allows tracking of the level of reactivation in a chromosome-X and genome-wide manner, thanks to the presence of hundreds of thousands of informative SNPs with respect to the more commonly used C57BL/6 or 129/Sv strains.

By using Bl6 females carrying an oocyte-specific *Zp3-Cre* transgene and a *Xist*^*2lox*^ allele, we have generated a maternal knockout of *Xist*. Crossing these females with *Mecp2*^*NLucTom*^ cast males has allowed us to generate *Xist*^−/+^:*Mecp2*^+/*NLucTom*^ embryos. An alternative model where the females carry the *Zp3-Cre*, *Xist*^*2lox*^, and *Mecp2*^*2lox*^ alleles has allowed us to generate *Xist*^−/+^:*Mecp2*^−/*NLucTom*^ embryos, that is, *Mecp2* knockouts based on expression. We have derived ESCs, MEFs, and NSCs from these F1 embryos. *Xist*^−/+^:*Mecp2*^+/*NLucTom*^ and *Xist*^−/+^:*Mecp2*^−/*NLucTom*^ NSCs show skewed XCI, as expected, by the presence of the *Xist* deletion on the maternal Bl6 X chromosome, while not showing any *in vitro* escape of *Mecp2* from the Xi. Why our *Xist*^−/+^:*Mecp2*^−/*NLucTom*^ neurons do not show RTT-related phenotypes is unclear. Most RTT-affected neuronal studies have been performed with *ex vivo* neuronal cultures (Baj et al., 2014; Rangasamy et al., 2016; Rietveld et al., 2015). However, *Mecp2* knockout neurons obtained by ESC differentiation showed smaller nuclear size than WT neurons after long-term culture *in vitro* (Yazdani et al., 2012). It is thus possible that our 10- or 11-day NSC differentiation is not sufficient to bring RTT phenotypes to the fore.

We have tested several compounds to assess whether the reporters can be reactivated. In contrast to what has been previously published (Przanowski et al., 2018), neither individual nor combined treatments with LDN193189, GSK650394, or RG2833 resulted in *Mecp2* reactivation in our *Xist*^−/+^:*Mecp2*^−/N*LucTom*^ NSCs. These differences might be due to Przanowski and colleagues using fibroblasts and adult brains instead of NSCs, or our NSCs might be more resilient to reactivation. Another possible reason for these drugs not to properly lead to XCR might be related to our cells being generated in a different and mixed genetic background. In addition, another inhibitor of ACVR1, K0228, also failed to reactivate a *Mecp2*-luciferase reporter in mouse tail fibroblasts (Lee et al., 2020). In conclusion, in our hands, combined treatment with GSK650394, LDN193189, and 5-Aza resulted in similar reactivation of *Mecp2* compared with 5-Aza only.

We have synergistically reactivated our NLuc-Tomato reporter with a combined treatment of 5-Aza and *Xist* knockdown. FACS analysis showed that a small population of treated cells shifted toward high Tomato fluorescence, while RNA-seq analysis indicated that a substantial population of cells in this fraction respond to the treatment and reactivate *Mecp2*, although, as expected, reactivation is not *Mecp2* specific. Eighty-five additional genes become significantly reactivated and several among these are more easily reactivated than *Mecp2*, and this will have to be taken into consideration when using general XCR methods with drugs as therapeutic treatments of RTT. Strikingly, we observed a significant overlap between our reactivated gene pool and genes reactivated at early and intermediate stages by means of iPSC reprogramming of female MEFs and female NPCs (Bauer et al., 2021; Janiszewski et al., 2019). It was intriguing to detect so many escapee genes listed in this last study as present in our reactivated gene list, suggesting these might be genes improperly silenced during their ESC differentiation process toward neuronal progenitors. In contrast, the NSCs in this study were isolated *de novo* from embryos and hence may have gone through a more robust XCI process *in vivo*. Altogether, we conclude that many X-linked genes show a predisposition to reactivate regardless of the technique, be it *Xist* knockdown combined with 5-Aza treatment or overexpression of the OCT4, SOX2, KLF4, or MYC transcription factors.

We examined which genetic or epigenetic mechanisms leading to XCR are at play here. Correlation analysis of reactivated genes with CpG presence and methylation loss after 5-Aza treatment indicates that although increased CpG presence is an indicator of reactivated genes, their reactivation surprisingly does not always seem to be associated with methylation loss. However, this can be reconciled with the fact that a small reduction in promoter methylation or loss of methylation at specific sites, not detectable by MeD-seq, might be sufficient for gene re-expression and may also explain why we detect only limited reactivation of *Mecp2* by NLuc activity analysis. We found that reactivated genes have decreased distances from escapees and that increased SINE and decreased LINE densities are potent indicators of reactivation. Correlating with our study, genes that are more easily silenced on the X chromosome or are ectopically silenced on an autosome carrying a *Xist* transgene tend to have more LINEs and fewer SINEs close to their TSSs (Loda et al., 2017). Moreover, in line with our results, X-linked genes that are reactivated early during iPSC reprogramming of female MEFs or NPCs harbor an increased number of SINEs closer to them than late or very late reactivating genes (Bauer et al., 2021; Janiszewski et al., 2019). There are thus strong indications that SINEs and LINES may play important roles in the capability of genes to be silenced or reactivated. SINE-mediated expansion of CTCF binding sites might explain why we detect increased binding of CTCF around reactivated genes on the Xa and an increased number of SINEs closer to reactivated genes (Bourque et al., 2008; Schmidt et al., 2012). Nevertheless, reactivated genes show an enrichment of all subclasses of SINEs irrespective of their type, and are thus not limited to CTCF-enriched SINE B2 transposable elements (Schmidt et al., 2012). Also, we could not find any correlation between reactivation and different LINE types, organized either by evolutionary age or by size, indicating that genes prone to reactivate have fewer LINEs nearby, independent of the LINE size or age. Although we could not study SINE and LINE methylation directly, we found that genomic regions surrounding SINEs and LINEs that are close to reactivated genes do not show methylation differences from SINEs and LINEs that are in the vicinity of non-reactivated genes.

How SINEs might be involved in silencing and reactivation of X-linked genes remains an open question, but SINEs may be involved in setting up higher-order chromatin structure to overcome gene repression. In addition, the deposition of H3K4me3, a mark of promoter activity on the Xa, also tends to correlate with XCR, indicating that genes with strong activity signatures on the Xa are more easily reactivated, probably owing to their higher capacity to attract transcription factors. Finally, reactivated genes show more presence of *Xist* at their TSSs than non-reactivated genes. This might be explained by their high activity signature when on the Xa, which tends to attract *Xist* more easily (Simon et al., 2013).

Finally, because higher-order chromatin structure may play an important role in reactivation, we interrogated the proclivity of X-linked TADs to reactivate. Likely because we selected a reactivated population based on Tomato fluorescence, we find that the TAD containing *Mecp2* is more easily reactivated than other TADs. Three other TADs also show a tendency to more easily reactivate than other TADs. Their tendency to reactivate correlates again with a higher presence of SINEs, CTCF, and H3K4me3 when on the Xa, in line with our results showing SINEs to be strong indicators of reactivation potential and increased CTCF and H3K4me3 signals at the TSS of reactivated genes on the Xa.

In conclusion, genes that are reactivated by *Xist* knockdown and 5-Aza treatment overlap significantly with genes that are reactivated by other means, namely during reprogramming of MEFs and NPCs toward iPSCs, suggesting general intersecting mechanisms for XCR. We describe here a new mouse model system that is more sensitive than any bioluminescent or fluorescent system currently available in the community to study reactivation of *Mecp2*, *in vitro* and *in vivo*; however, RTT reversal phenotypes that occur on *Mepc2* reactivation will have to be studied with NSCs differentiated *in vitro* into neurons for longer, *ex vivo* neurons, or *in vivo*. These mouse lines could be used to study *Mecp2* reactivation by high-throughput screening of chemical compounds or by more targeted approaches, such as CRISPR-Cas9 fused to activators or repressors.

## Experimental procedures

### Mouse lines

All animal experiments were performed according to the legislation of the Erasmus MC Rotterdam Animal Experimental Commission. *Xist*^*2lox*^ mice were crossed with *Mecp2*^*2lox*^ mice to generate a colony of *Xist*^*2lox/2lox*^*:Mecp2*^*2lox/2lox*^ mice. *Xist*^*2lox/2lox*^ and *Xist*^*2lox/2lox*^*:Mecp2*^*2lox/2lox*^ female mice were crossed with male *Zp3-Cre* mice to generate *Xist*^*2lox*/+^ *Zp3-Cre* and *Xist*^*2lox*/+^:*Mecp2*^*2lox*/+^ *Zp3-Cre* females. These were then crossed with cast *Mecp2*^*NLucTom/Y*^ males to generate *Xist*^−/+^:*Mecp2*^+/*NLucTom*^, *Xist*^−/+^:*Mecp2*^−/*NLucTom*^, and *Xist*^+/+^:*Mecp2*^+/*NLucTom*^ female hybrid embryos. *Mecp2*^*NLucTom/NLucTom*^ female embryos were obtained from the *Mecp2*^*NLucTom*^ cast colony. WT hybrid females were obtained by crossing Bl6 females with cast males.

### Cell culture

All ESC lines were grown in a regular ESC medium (DMEM, 10% fetal calf serum, 100 U mL^−1^ penicillin/streptomycin, 0.1 mM 2-mercapoethanol, 0.1 mM non-essential amino acids [NEAA], 5,000 U mL^−1^ leukemia inhibitory factor [LIF]) supplemented with 2i (1 μM PD0325901, Selleckchem; 3 μM CHIR99021, Axon Medchem) on irradiated male MEFs. An extended description of cell isolations and growth culture conditions is provided in the supplemental information.

### RNA sequencing and MeD-seq

A detailed description is provided in the supplemental information.

## Author contributions

H.M.-B. contributed to the design of the study, performed experiments, analyzed the data, interpreted the results, and wrote the manuscript. B.T. analyzed the data, interpreted the results, and edited the manuscript. C.G. and S.G. performed experiments, analyzed the data, and interpreted the results. R.G.B. and J.B. analyzed the data and interpreted the results. C.D. performed experiments. M.v.R. and W.v.I. analyzed the data. P.F. and A.B. contributed to the design of the study. J.G. contributed to the design of the study and to the interpretation of the results and edited the manuscript.

## Supporting citations

The following reference appears in the supplemental information: Csankovszki et al., 1999.

## Conflict of interests

The authors declare no competing interests or financial interests, except for R.B., J.B., W.v.I., and J.G., who report being shareholders in Methylomics B.V., a commercial company that applies MeD-seq to develop methylation markers for cancer staging. J.G. is a co-founder of Methylomics and a member of its scientific advisory board.

## Data Availability

All raw and processed high-throughput sequencing data (RNA-seq, MeD-seq) generated in this study have been submitted to the NCBI Gene Expression Omnibus (GEO) under accession number https://www.ncbi.nlm.nih.gov/geo/query/acc.cgi?acc=GSE166147.

## Supplemental information

Document S1. Supplemental experimental procedures, Figures S1–S5, and Table S1

Table S2. X-linked differential expression analysis between the Tomato-High and control populations This table shows the differentially expressed X-linked genes between the Tomato-High and control populations using DESeq2. Genes are annotated according to their gene group, and X-inactivated genes that are reactivated in the Low and Med samples are indicated.

Table S3. DNA methylation analysis of X-linked promoters using MeD-seq This table shows the normalized MeD-seq reads of male, Tomato-High, and control populations at the X-linked gene promoters (±1kb of the TSS). Fold change between Tomato-High and control are reported, as well as the p value based on a Mann-Whitney test. Genes are annotated according to their gene class based on the RNA-seq data.

Document S2. Article plus supplemental information

## Footnotes

Supplemental information can be found online at https://doi.org/10.1016/j.stemcr.2022.01.008.
