# Supplementary material for: Genetic and epigenetic determinants of reactivation of Mecp2 and the inactive X chromosome in neural stem cells
Source: Stem Cell Reports. 2022 Feb 10;17(3):693–706. doi: 10.1016/j.stemcr.2022.01.008 (PMC9039756; doi:10.1016/j.stemcr.2022.01.008)
Supplement: Document S1. Supplemental experimental procedures, Figures S1–S5, and Table S1 [file mmc1.pdf]

Stem Cell Reports, Volume *17*

Supplemental Information

Genetic and epigenetic determinants of reactivation of Mecp2 and the inactive X chromosome in neural stem cells

H. Mira-Bontenbal, B. Tan, C. Gontan, S. Goossens, R.G. Boers, J.B. Boers, C. Dupont, M.E. van Royen, W.F.J. IJcken, P. French, A. Bedalov, and J. Gribnau

Supplemental Information

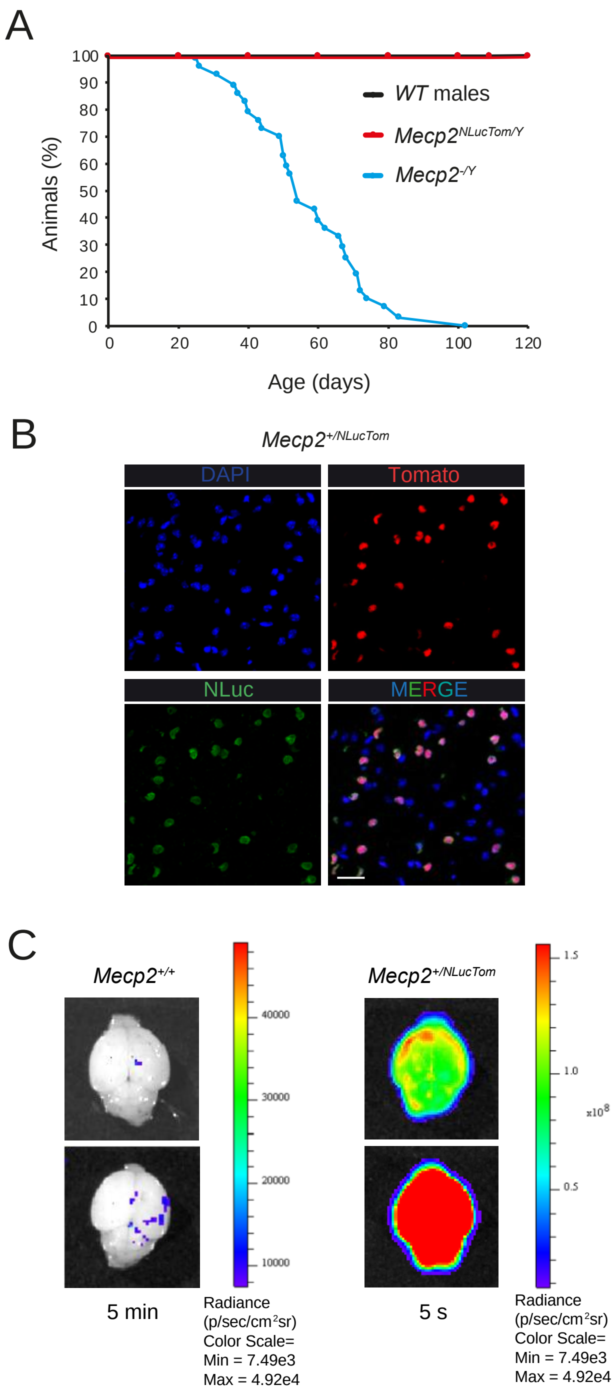

Figure S1. Cast *Mecp2-NLucTom* mice are viable and express the fusion protein in the brain (related to Figure 2).

(A) Cumulative survival plot showing the % of cast *Mecp2*$^{NLucTom/Y}$ males (red) compared to WT cast males (black) and *Mecp2*$^{\Delta/Y}$ males (blue)(Guy et al., 2001) surviving at a given time in days. (B) IF of NLuc (green) and endogenous Tomato fluorescence (red) in brain sections of heterozygote *Mecp2*$^{+/NLucTom}$ females showing random XCI. DAPI, blue. We quantified 86 cells

out of 193 cells (45%) from a single brain showing NLuc and Tomato signals. White scale bar: 25 µm.

(C) Representative bioluminescence images of two P6 WT female brains and two P6 *Mecp2*$^{+/NLucTom}$ female brains after 5 min or 5 s, respectively, of furimazine injection.

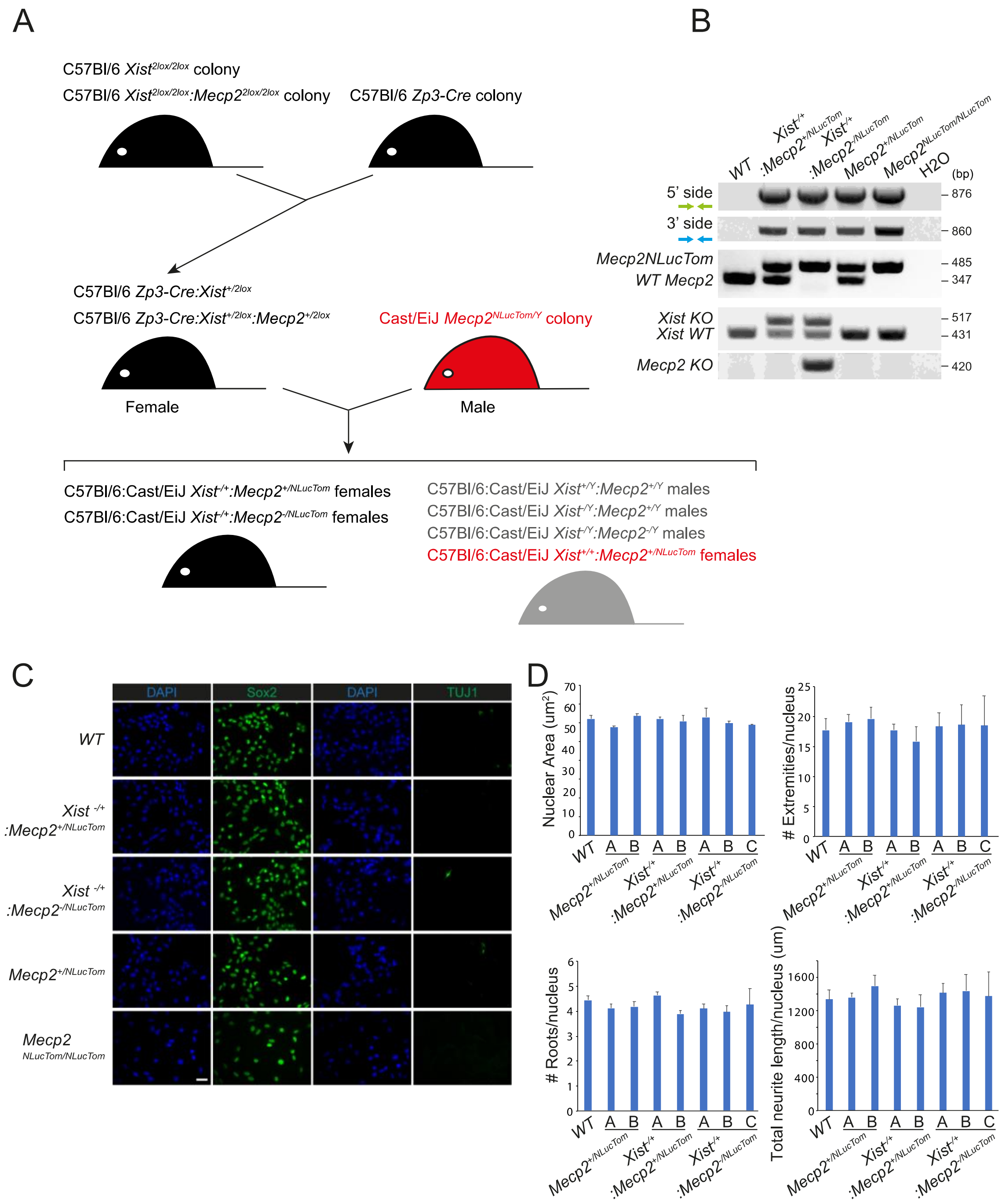

Figure S2. Generation of WT, *Xist*$^{-/+}$:*Mecp2*$^{+/NLucTom}$, *Xist*$^{-/+}$:*Mecp2*$^{-/NLucTom}$, *Mecp2*$^{+/NLucTom}$ and *Mecp2*$^{NLucTom/NLucTom}$ NSCs (related to Figure 2).

(A) Breeding strategy to generate *Xist*$^{-/+}$:*Mecp2*$^{+/NLucTom}$, *Xist*$^{-/+}$:*Mecp2*$^{-/NLucTom}$ embryos and associated lines. We keep four independent colonies in two backgrounds: C57BL/6 *Xist*$^{2lox/2lox}$, C57BL/6 *Xist*$^{2lox/2lox}$:*Mecp2*$^{2lox/2lox}$, C57BL/6 *Zp3-Cre* and Cast/EiJ *Mecp2*^{NLucTom} mice. *Xist*$^{2lox/2lox}$ and *Xist*$^{2lox/2lox}$:*Mecp2*$^{2lox/2lox}$ females or males are crossed with *Zp3-Cre* mice to generate heterozygous *Zp3-Cre:Xist*$^{+/2lox}$ and *Zp3-Cre:Xist*$^{+/2lox}$:*Mecp2*$^{+/2lox}$ females in a C57BL/6

background. These females are then crossed with *Mecp2*$^{NLucTom/Y}$ males, to generate hybrid *Xist*$^{-/+}$*:Mecp2*$^{+/NLucTom}$ or *Xist*$^{-/+}$*:Mecp2*$^{-/NLucTom}$ female embryos. Other possible genotypes of this last crossing are depicted in gray. Notice all genotypes from this second crossing can have Zp3-Cre (50% probability), not depicted in figure.

(B) Genotyping PCR of F1 NSCs with different genotypes. Primers for the 5' side and 3' side of the specific integration are the same primers as in Figure 1C. PCRs for the *Xist* WT and knockout alleles and *Mecp2* WT, tagged with NLucTom or knockout alleles are also shown. Note that *Xist*$^{-/+}$*:Mecp2*$^{-/NLucTom}$ cells do not show any WT Mecp2 allele since it is deleted on the maternal X chromosome.

(C) IF of Sox2 or TUJ1 (left and right green respectively) in WT, *Xist*$^{-/+}$*:Mecp2*$^{+/NLucTom}$, *Xist*$^{-/+}$*:Mecp2*$^{-/NLucTom}$, *Mecp2*$^{+/NLucTom}$ and *Mecp2*$^{NLucTom/NLucTom}$ NSCs. DAPI, blue. White scale bar: 25 µm. n=1.

(D) Nuclear area, number of cellular extremities per nucleus and number of roots per nucleus (average ± s.d., n=3 biological replicates, 191-521 neurons per replicate) of 2-3 independent WT, *Xist*$^{-/+}$*:Mecp2*$^{+/NLucTom}$ and *Xist*$^{-/+}$*:Mecp2*$^{-/NLucTom}$ NSC clones differentiated to neurons.

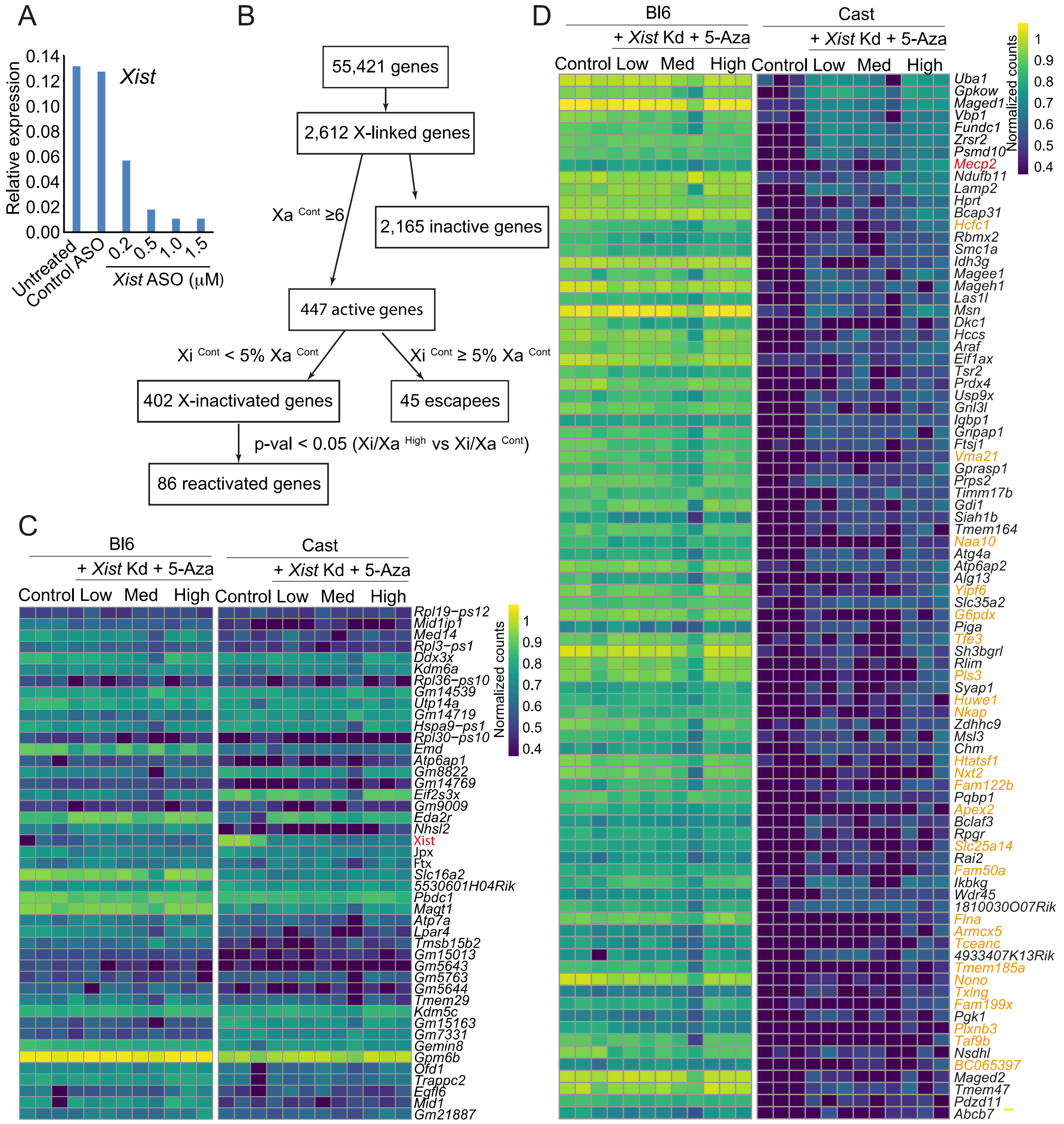

Figure S3. Many X-linked genes are reactivated in mNSCs after *Xist* knockdown and 5-Aza treatment (related to Figures 3 and 4).

(A) Relative *Xist* expression by RT-qPCR analysis in *Xist*$^{-/+}$*:Mecp2*$^{+/NLucTom}$ NSCs after knockdown of *Xist* with *Xist* ASOs or control ASOs. Different concentrations of *Xist* ASOs were tested, n=1.

(B) Flowchart indicating the general steps performed to obtain the different gene subclasses from the RNA-seq analysis.

(C) Expression heatmap of the different escapees across the different samples and alleles. *Xist* is indicated in red.

(D) Expression heatmap of the reactivated genes across the different samples and alleles, ordered by P-value. *Mecp2* is indicated in red. Genes that are also reactivated in the Tomato-Low

and/or Tomato-Medium populations are indicated in black, while genes only significantly reactivated in the Tomato-High population as *Mecp2* are indicated in orange.

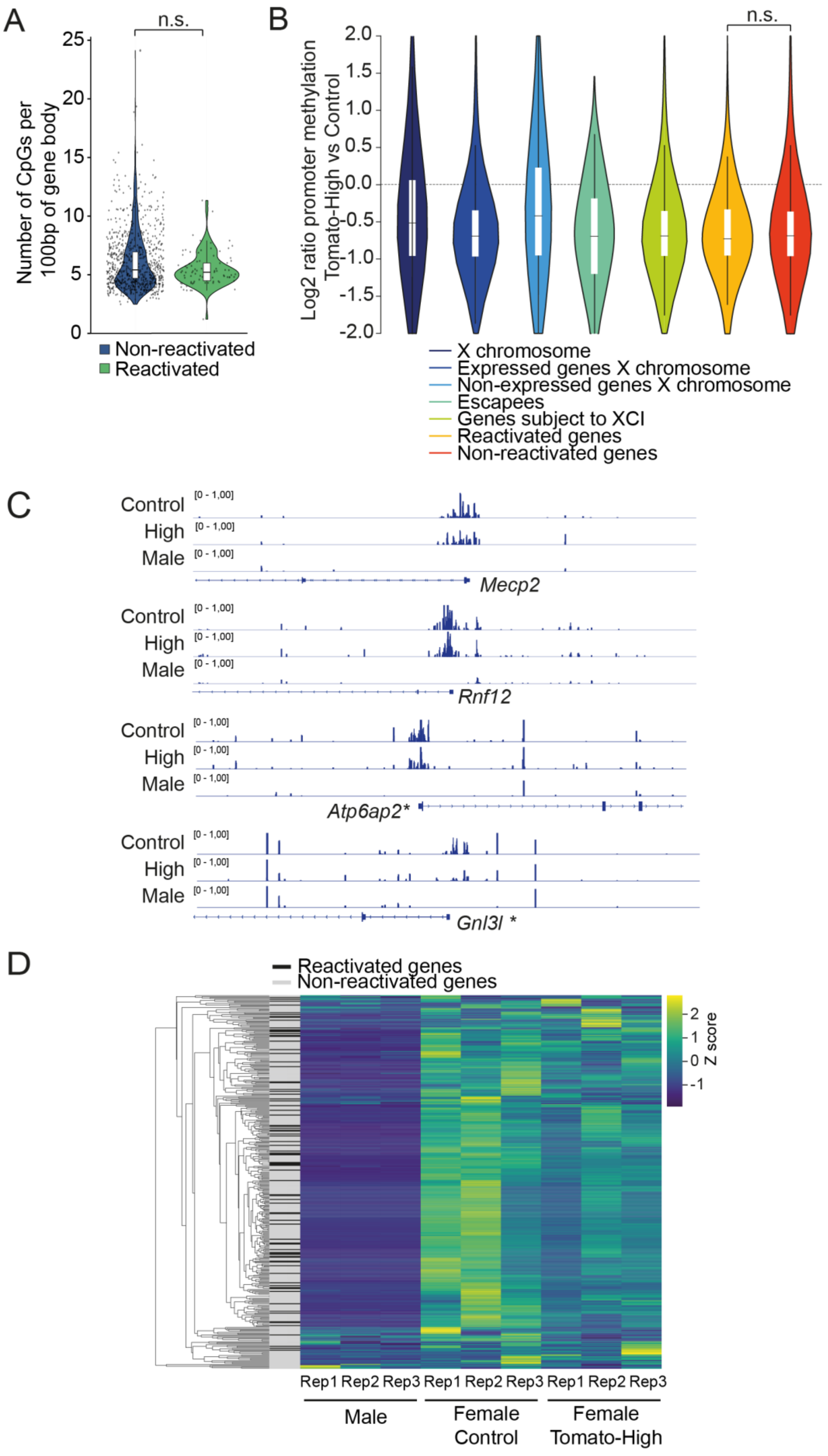
A
n.s.
Number of CpGs per 100bp of gene body
25
20
15
10
5
0
Non-reactivated
Reactivated
B
n.s.
Log2 ratio promoter methylation Tomato-High vs Control
2.0
1.5
1.0
0.5
0.0
-0.5
-1.0
-1.5
-2.0
X chromosome
Expressed genes X chromosome
Non-expressed genes X chromosome
Escapees
Genes subject to XCI
Reactivated genes
Non-reactivated genes
C
Control
High
Male
Mecp2
Rnf12
Atp6ap2*
Gnl3l *
D
Reactivated genes
Non-reactivated genes
Z score
2
1
0
-1
Rep1 Rep2 Rep3 Rep1 Rep2 Rep3 Rep1 Rep2 Rep3
Male
Female Control
Female Tomato-High

Figure S4. Reactivation does not correlate with a general loss of methylation at promoters (related to Figure 5).

(A) Violin plot depicting the number of CpGs per 100bp of gene body of reactivated and non-reactivated genes; n.s., not significant (Mann-Whitney test, p-value < 0.05).

(B) Violin plots depicting the log2 ratio between the methylation status of the promoter (± 1 Kbp around TSS) of the different gene subclasses in the Tomato-High and control populations. Dotted line indicates an identical methylation status for both conditions (ratio = 1); n.s., not significant (Mann-Whitney test, p-value < 0.05).

(C) Genome browser overview with the average normalized MeD-seq tracks at the promoter areas of four reactivated genes *Mecp2*, *Rnf12, Atp6ap2* and *Gnl3l* in female control, female Tomato-High and male NSCs. Genes with significant loss of DNA methylation at their promoters (±1 Kbp of the TSS) are indicated by an asterisk.

(D) Heatmap of the DNA methylation status around the TSSs of reactivated genes and non-reactivated genes of the three biological replicates of female control, female Tomato-High and male NSCs. Z-scores of MeD-seq read counts ±1 Kbp of the TSS are shown. Next to the clustering dendrogram, genes are annotated as reactivated and not-reactivated in black and gray, respectively.

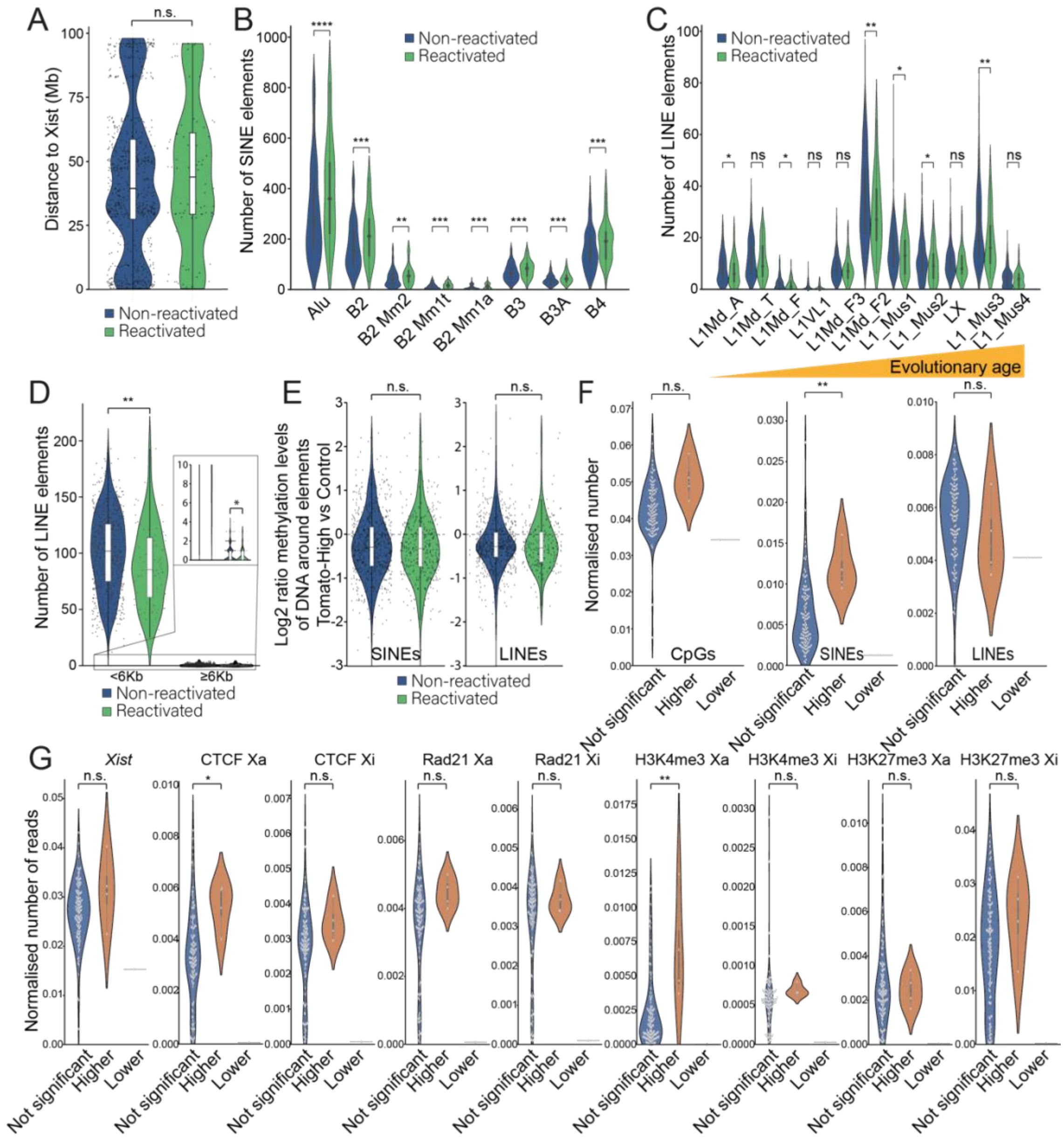

Figure S5. Reactivation correlates with several genetic and epigenetic marks (related to Figure 5).

(A) Violin plots depicting the distance to *Xist* in Mb of non-reactivated genes (blue) and reactivated genes (green). n.s., not significant (Mann-Whitney test, p-value < 0.05).

(B) Violin plots depicting the number of different SINE subclasses in ± 500 Kbp bins around the TSS of non-reactivated genes (blue) and reactivated genes (green). ** p-val<5E-3, *** p-val<5E-4, **** p-val<5E-5, Mann-Whitney test.

(C) Violin plots depicting the number of different LINE subclasses organized by evolutionary age (left, young; right, old) in ± 500 Kbp bins around the TSS of non-reactivated genes (blue) and reactivated genes (green). * p-val<0.05, ** p-val<0.01, Mann-Whitney test.

(D) Violin plots depicting the number of LINEs organized by size (<6 Kbp or ≥6 Kbp) in ± 100 Kbp bins around the TSS of non-reactivated genes (blue) and reactivated genes (green). *, p-val<0.05, ** p-val<0.01, Mann-Whitney test.

(E) Violing plots depicting the levels of DNA methylation around LINEs and SINEs (±200 bp) situated ± 100 Kbp around the TSSs of non-reactivated genes (blue) and reactivated genes (green) in the Tomato-High population vs Control.

(F) Violin plots of the normalized number of CpGs, SINEs and LINEs in TADs that are significantly enriched (higher) or depleted (lower) for reactivated genes or not significant. ** p-val<0.01, Mann-Whitney test; n.s. not significant.

(G) Violin plots of the normalized number of CHART-seq reads of *Xist* presence and ChIP-seq reads of CTCF, Rad21, H3K4me3 and H3K27me3 on the Xa and Xi in TADs that are not significantly enriched for reactivated genes and TADs that are significantly enriched (higher) or depleted (lower) for reactivated genes. * p-val<0.05, ** p-val<0.01, Mann-Whitney test; n.s., not significant.

		Forward	Reverse
PCR	3' side	ACTGTGCTCCCCATCAAGAA	AGTGTGCCATAGTGCAGGAT
PCR	5' side	TCCCACAACGAGGACTACAC	TCCATGGCACCTCTTTAGCA
PCR	Endogenous/ WT *Mecp2*	ATGCTCCAACTACTCCCACC	CCAAGGAGCCAGCTAAGACT
PCR	*Rnf12* control	GCCTTCGAACATCTCTGAGC	GAGCCGGACTAATCCAAACA
PCR	*Xist 2lox/null/*WT *alleles*	CACTGGCAAGGTGAATAGCA and ACCCTTGCCTTTTCCATTTT	CTTCTGGTCTTTGAGGGCAC
PCR	*Mecp2-NLuc-Tom*	CGAGAGAGTTAGCGGATCCA	TTACTCGGAACAGCAGGGAG
PCR	Mecp2 null allele	TGG TAA AGA CCC ATG TGA CCC AAG	TCCACCTAGCCTGCCTGTACT TTG
RT-qPCR	*b-actin*	AACCCTAAGGCCAACCGTGAA AAG	CATGGCTGGGGTGTTGAAGGT CTC
RT-qPCR	*Xist*	GGATCCTGCTTGAACTACTGC	CAGGCAATCCTTCTTCTTGAG
RT-qPCR	*NLuc*	GGCTACAACCTGGACCAAGT	TGGATCGGAGTTACGGACAC

Table S1. Primers used in this study (related to Figures 1 and S2).

Table S2. X-linked differential expression analysis between the Tomato-High and Control populations.

This table shows the differentially expressed X-linked genes between the Tomato-High and Control populations using DESeq2. Genes are annotated according to their gene group and X-inactivated genes that are reactivated in the Low and Medium samples are indicated.

Table S3. DNA methylation analysis of X-linked promoters using MeD-seq.

This table shows the normalized MeD-seq reads of male, Tomato-High and Control populations at the X-linked gene promoters (± 1kb of the TSS). Fold change between Tomato-High and Control are reported, as well as the p-value based on a Mann-Whitney test. Genes are annotated according to their gene class based on the RNA-seq data.

Supplemental Experimental Procedures

Mouse lines

All animal experiments were performed according to the legislation of the Erasmus MC Rotterdam Animal Experimental Commission.

Mecp2^{NLucTom} mice were obtained from *Mecp2*^{NLucTom} ESCs generated in house (see below) and kept in a cast background colony. *Xist*^{2lox} mice (Csankovszki et al., 1999) were kept in a B6 background. *Mecp2*^{2lox} and *Zp3-Cre* mice were obtained from the Jackson Laboratory (B6.129P2-MeCP2tm1Bird/J - #077177, C57BL/6-Tg(Zp3-Cre)93Kw/J - #003651 respectively) and kept in a B6 background. A detailed description of the crosses is provided in supplementary experimental procedures.

Cell culture

ESCs were generated from E3.5 blastocysts. Briefly, E3.5 blastocysts were flushed from uteri in M2 medium. Their zona pellucida was removed with acidic Tyrode's solution (Sigma) at RT for several seconds. Embryos were subsequently washed in M2 medium and transferred to 4-well plates, one blastocyst per well, containing irradiated MEFs and regular ESC medium supplemented twice as much as the normal amount of LIF and PD98059 (final concentration 50 µM; Cell Signaling Technologies). ICM outgrowths were then picked 5-7 days plating the blastocyst and expanded. Once ESCs reached the 12 well stage, they were genotyped. The selected genotypes were grown as single cells/colonies by plating them in serial dilutions in 10 cm dishes. One day after plating, cells were weaned off the increased concentration of LIF and of PD98059. Colonies were picked, selected for morphology and correct karyotype.

To target *Mecp2* in male cast ESC cells, 0.6x10$^{\wedge 6}$ ESCs were transfected with 2 µg CRISPR/Cas9 targeting the *Mecp2* STOP codon and 2 µg of the donor vector carrying 5' and 3' 500bp-long cast-specific homology arms with a NLuc and Tomato reporters in frame with *Mecp2*'s coding region. A P2A signal between NLuc and Tomato leads to Tomato being translated as an independent protein. ESCs were transfected with 4 µL lipofectamine 2000 following the manufacturer's instructions (ThermoFisher). 2 days after transfection, Tomato-positive cells were FACS-sorted, put back into culture for a few days and subsequently injected in B6 blastocysts.

MEFs were isolated from E12.5 embryos. E12.5 embryos were removed from yolk sacs and their heads, liver, hearts and digestive tract were removed. The remaining carcass was chopped into fine pieces and added to 5 mL of Trypsin-EDTA (Life Technologies), for 10 min 37°C in water batch. Falcons were shaken smoothly every 2 min. Remaining clusters and cells were pipetted up and down several times. Medium was added to quench the trypsin and the sample was centrifuged 5 min 1000 rpm. Cell pellet was resuspended in regular ESC medium without LIF and 2i and grown in 0.2% gelatin-coated 15cm dishes. After 3x 15 cm dishes were obtained, cell lines were genotyped and frozen.

NSC lines were isolated from E15.5 embryos. E15.5 brains were extracted, hemispheres were cut and meninges dissected when possible. Cortexes were chopped into pieces and introduced in 15 mL falcon tubes containing 2.5 mL dissecting medium (PBS +3% glucose) plus 300 µL Trypsin-EDTA (10x; Life Technologies, 15400-054). The falcon was then incubated for 10 min at 37°C while shaking it every 2 min manually. Trypsin was then inactivated with 500 µL horse serum (Life Technologies, 16050-130). 50 µL DNaseI (1mg mL^{-1}) was added and incubated for 8-10 min at 37°C. Pipette up and down around 10 times with 1 mL pipette. Centrifuge 1000 rpm 5 min and resuspend in filtered NSC culture medium (Conti 2005): 192 mL EuroMed-N (EuroClone), 2 mL N2 supplement (Invitrogen), 1 mL human insulin (final concentration 20 µg mL^{-1}, Roche), 1ml BSA (final concentration 50 µg mL^{-1}, Gibco), 2 mL L-Glut (100x; Gibco), 10 µL murine EGF (final concentration 10 ng mL^{-1}, Peprotech), 10 µL human bFGF (final concentration 10 ng mL^{-1}, Peprotech), 100 U mL^{-1} penicillin/streptomycin. Cells were grown in suspension in 10cm dishes for a week. Resulting neurospheres were disaggregated and cells were grown as a monolayer. The different lines were established and grown in 6-well plates. Wells were precoated with 0.2% gelatin for 5 min RT, then removed and 1.5 mL Laminin (final concentration 5 µg mL^{-1} in PBS; Sigma) was added to wells for at least 5 h at 37°C, better o/n at 37°C, or kept in the fridge with parafilm for up to a month. Prior to use, wells were washed quickly 3x with PBS and cells added. Cells were passaged with Accutase (Sigma) every 3-5 days up to passage 10-12, generating frozen vials along the way.

NSC differentiation into neurons, astrocytes and oligodendrocytes was performed following the protocol by Spiliotopoulos and colleagues (Spiliotopoulos et al., 2009). NSCs were counted and seeded in 5 mL D1 medium (EuroMed-N, 0.5% N2 (Invitrogen), 1% B27 (Invitrogen),

10 ng mL^{-1} human bFGF and 100 U mL^{-1} penicillin/streptomycin) at 1.35x10^{5} cells cm^{-2} per well of a 6-well dish previously coated with 0.1% gelatin and 5 µg mL^{-1} laminin in PBS for at least 5 hours (day 0). 3 days after plating, cells were collected with accutase, counted and reseeded in 3 mL A medium (1:3 mix DMEM/F12 (Gibco) and Neurobasal (Gibco), 0.5% N2, 1% B27, 10 ng mL^{-1} bFGF, 20 ng mL^{-1} BDNF (Prospec) and 100 U mL^{-1} penicillin/streptomycin) at 5 x10^{4} cells cm^{-2} per well of a 6-well dish with coverslips precoated with gelatin and laminin (day 3). Medium was changed at day 5 and at day 6, medium was changed by B medium (1:3 mix DMEM/F12 and Neurobasal, 0.5% N2, 1% B27, 6.7 ng mL^{-1} bFGF, 30 ng mL^{-1} BDNF and 100 U mL^{-1} penicillin/streptomycin). At day 9, medium was changed to B1 medium (same as B medium although at 5 ng mL^{-1} bFGF). Coverslips were then fixed at day 10-11 after the start of differentiation following the IF protocol below.

Immunofluorescence

Mice were perfused with 4% PFA for 3 min. Brains were removed and fixed again in 4% PFA for 1 h at RT. Brains were left to sink o/n in 10% sucrose in PBS at 4°C and frozen in OCT the next day by bathing a freezing cup isopentane in dry ice and stored afterwards at -80°C. 7 µm coupes were generated with a cryostat microtome and put on adhesive slides. Slides were left to dry 30 min at RT and frozen at -80°C. Slides were thawed 30 min at RT and processed with the same IF protocol as cell cultures, see below.

IF on cell cultures were performed as follows. Cells were grown on coverslips and subsequently blocked in 5% goat serum (Sigma, G9023; or donkey serum when necessary, Sigma, D9963), 1% Triton X100 (performed) in PBS for 1 h at RT. The primary antibody was applied in PBS 5% goat (or donkey) serum, 0.1% Triton X1000 0/n at 4°C in the dark. The next day, slides were washed 3x with 5% goat (or donkey) serum, 0.1% Triton X100 in PBS for 5 min at RT. The second antibody was then applied in 5% goat (or donkey) serum, 0.1% Triton X100 in PBS for 1 h at RT and washed 3x with 5% goat (or donkey) serum, 0.1% Triton X100 in PBS for 5 min at RT (last wash containing 1:5000 DAPI). Slides were then mounted with Prolong™ Gold Antifade Mountant (Thermo Scientific). Images were acquired with a fluorescent Axio Imager M2 microscope (Zeiss) and analysed with Fiji and Photoshop software (Adobe). The following primary antibodies were used: mouse anti-TUJ1 (Biolegend, 801202, 1:250), rabbit anti-GFAP (Dako,

Z0334, 1:250), rabbit anti-OLIG2 (IBL, 18953, 1:250), goat anti-SOX2 (Santa Cruz, sc-17320, 1:500), rabbit anti-NLuc (kind gift of Promega, 1:100). The following Alexa Fluor secondary antibodies were used: goat anti-rabbit 488 (Invitrogen, A-11008, 1:400), goat anti-mouse 488 (Invitrogen, A-11001, 1:400) and goat anti-rabbit 633 (Invitrogen, A-21070, 1:400), donkey anti-goat 488 (Invitrogen, A-11055, 1:400). IFs of neuronal cultures were not performed with serum, BSA was used instead.

Xist knockdown and drug analysis

Xist knockdown was performed following manufacturer's instructions for the Mouse Neural Stem Cell Nucleofector Kit (Lonza). 3-5x10^6 NSCs were collected and resuspended in 70 µL Nucleofector solution, 15 µL supplement and 15 µL *Xist* ASO 10 µM (*XIST*-ANAND_1, cat. 339511 LG00116620-DDA: TCTTGGTTACTAACAG (Carrette et al., 2018); Qiagen). Cells were nucleofected with a Lonza Nucleofector™, program A-033. Cells were then put back into culture and treated with 5-Aza or its vehicle for 3 days.

Decitabine (Selleck Chem) was resuspended in aliquots of 10 µL 10 µM in DMSO and kept in an Argon atmosphere at -80°C. During the 7-day drug test, LDN193189, GSK650394, RG2833 and decitabine were used at 0.5 µM, 2.5 µM, 5 µM and 0.5 µM respectively (Carrette et al., 2018; Janiszewski et al., 2019). The RNA-seq analysis was performed on NSCs treated with 10 µM 5-Aza and 1.5 µM *Xist* ASO, or DMSO and 1 µM scrambled ASOs for 3 days.

Western Blot

Cells were harvested in ice-cold PBS with complete protease inhibitors (Roche). Cell pellets were incubated with 400 µL Buffer A (100 mM HEPES, 1.5 mM MgCl2, 10 mM KCl, 0.5 mM DTT and protease inhibitors) for 10 min on ice, vortexed 30 sec and centrifuges 2000 rpm, 5 min, 4ºC. Nuclei were then lysed by adding 2x the pellet volumes of Buffer C (20 mM HEPES, 25% glycerol, 420 mM NaCl, 1.5 mM MgCl2, 0.2 mM EDTA, 0.5 mM DTT and protease inhibitors) for 20 min on ice, centrifuged max speed, 2min, 4ºC. Protein concentrations were determined with NanoDrop. WB was performed with homemade SDS-PAGE gels and nitrocellulose membranes (Merck). The following antibodies were used, mouse anti-MECP2 (Sigma-Aldrich,

M7443, 1:500), rabbit anti-RFP (Abcam, ab62431, 1:500), rabbit anti-NLuc (kind gift of Promega, 1:1000) and b-actin-peroxidase (Sigma, A3854, 1:20,000). Detection of peroxidase activity was performed using ECL western blotting detection reagent (GE Healthcare) in an Amersham Imager 600 (GE Healthcare). Detection of the remaining proteins was performed with an Odyssey CLx imaging system with Imago Studio 5.2 software (LI-COR Biosciences) with the following antibodies: IRDye 800CW donkey anti-mouse and IRDye 680RD donkey anti-rabbit (both from LI-COR Biosciences, 926-32212 and 926-68073 respectively,1:10,000).

In vitro and *in vivo* NLu Assays

NLuc assays were performed following manufacturer's instructions (Nano-Glo® Luciferase Assay System, Promega). Briefly, cells were collected and counted. A specific amount of cells (<0.5x10^6 cells) were resuspended in 25 µL PBS, and added to a well of a 96-well dish (White Cliniplate, Thermo Scientific) and 25 µL of Nano-Glo® Luciferase Assay Substrate + Buffer were added. Cells were lysed for 3 min and bioluminescence was then read in a VICTOR X4 Multilabel Plate Reader (1 s reading, 3 technical replicates, 10 s interval between readings).

P6 mice were anesthetized by isoflurane inhalation and injected intraperitoneally with furimazine (kindly provided by Promega). In short, solid furimazine was resuspended in 100% ethanol and kept for short periods of times at -80ºC. Before injections, furimazine was resuspended in 8% glycerol, 10% ethanol, 10% hydroxypropyl-β-cyclodextrin and 35% PEG400 in water, as in (Yeh et al., 2017). Anesthetized mice were injected with 5 µg furimazine/ g mouse. Briefly after injection, mice were sacrificed and their brains extracted for imaging in an IVIS Spectrum imager (PerkinElmer). Brains were imaged in an open filter with an exposure time of 5 s (*Mecp2*$^{+/NLucTom}$) or 5 min (*Mecp2*$^{+/+}$). Brain radiance (p/s/cm^2/sr) was measured from same-sized regions of interest and corrected by subtracting the background signal.

RNA-sequencing

DNA libraries from RNA samples (3 independent biological replicates) were prepared using the Smart-seq2 method and subsequently sequenced on an Illumina HiSeq2500 sequencer. The 50 bp single-end RNA-seq reads were processed allele-specifically. The SNPs

in the C57BL_6NJ and Cast/Ei lines were downloaded from the Sanger institute (v.5 SNP142)(Keane et al., 2011). These were used as input for SNPsplit v0.3.4 (Krueger and Andrews, 2016) to construct an N-masked reference genome based on mm10 in which all SNPs between C57BL_6NJ and Cast/Ei were masked. Reads were first mapped to a reference genome file containing the C57BL genome, Cast/Ei genome and the NLuc sequence using the default settings of hisat2 v2.2.1 (Kim et al., 2015). Reads that mapped to the NLuc sequence without mismatches were removed from the fastq files, after which the remaining reads were remapped to the N-masked reference genome. SNPsplit was then used to assign the reads to either the C57BL_6NJ or Cast/Ei bam file based on the best alignment or to a common bam file if mapping to a region without allele-specific SNPs. The allele-specific and unassigned bam files were sorted using samtools v1.10 (Li et al., 2009). The number of mapped reads per gene were counted for both alleles separately using HTSeq v0.12.4 (--nonunique=none -m intersection-nonempty) (Anders et al., 2015) based on the gene annotation from ensembl v98. For each sample, the number of reads that mapped perfectly to the NLuc sequence was added to the *Mecp2* gene count of the Cast/Ei allele. For each condition, genes with more than 20 allele-specific reads across the triplicates were used to calculate the allelic ratio, defined as Xi/(Xi+Xa) where the inactive X (Xi) and active X (Xa) are Cast/Ei and C57BL, respectively. The difference between the allelic ratios of X-linked genes between the High and Control samples were plotted along the X chromosome using only genes with more than 20 allele-specific reads in both conditions.

We filtered the X-linked genes based on the number of reads overlapping Xa and Xi of the control samples and the High samples separately. Active genes were selected as X-linked genes with at least 6 reads overlapping Xa of the control samples, whereas inactive genes were genes with less than 6 reads overlapping the $Xa^{Control}$. Escapee genes were selected as active genes with $Xi^{Control} \geq 5\% \; Xa^{Control}$, whereas the remainder of the genes ($Xi^{Control} < 5\% \; Xa^{Control}$) are labelled as X-inactived genes. To find the reactivated genes, we performed a differential expression analysis using DEseq2 v1.26.0 (Love et al., 2014), resulting in a list of genes with a significant allelic difference between High and Control. Reactivated genes were selected as X-inactivated genes that were also significantly differentially expressed (p-value < 0.05). For plotting, the counts of all genes were normalized using the variance stabilizing transformation (vst) function. For the plots with the low and medium conditions, DESeq2 was run on all four

conditions where the control samples were compared against the 5-Aza samples (*i.e*. low, medium and high conditions) and counts were normalized once more. We also performed differential expression analyses between Low and Control and between Medium and Control and compared the lists of significant genes to the reactivated genes in the High samples.

We compared our lists of reactivated and escapee genes to lists of genes from other studies using venn diagrams. For the comparison with (Janiszewski et al., 2019), we downloaded the allelic ratios (Mus/(Mus+Cast)) of all X-linked genes and defined genes as escapee, early, intermediate, late, very late and escapee when they were biallelically expressed (ratio between 0.15 and 0.85) at day 2, 8, 10, 13 or 15, respectively. Our list of reactivated genes was also compared to data from (Bauer et al., 2021). For each X-linked gene, we calculated the allelic ratio (Mus/(Mus+Cast)) and selected X-inactivated genes and escapees as X-linked genes based on their allelic ratio in NPCs, *i.e.* an allelic ratio < 0.14 or ≥ 0.14, respectively. The list of X-inactivated genes was divided in early and late reactivated genes based on the first day with an allelic ratio ≥ 0.14 where early was defined as genes reactivated in the samples D4 SSEA1+, D4 P-RFP+, D5 RFP+ or D6 RFP+ and late as genes reactivated in D6 X-GFPint, D6 X-GFP+, D7 X-GFP+, D7 X-GFP+, D8 X-GFP+, D9 X-GFP+ or D10 X-GFP+.

The genes from the different gene classes were compared based on several characteristics. We extracted the CpG sites from the mm10 reference genome, and counted the number of CpG sites in the region 2 Kbp upstream of the TSS to the TSS of each gene using BEDTools coverage v2.29.2 (Quinlan and Hall, 2010). Moreover, we counted the number of CpG sites overlapping the gene body of each gene and normalized by dividing this number by the gene length*0.01 to obtain the number of CpGs per 100bp of the gene body. For each gene, the distance from the TSS to the nearest escapee from our escapee list (see paragraph above) and *Xist* was identified using BEDTools closest. A table containing the locations of SINE and LINE repeat elements was downloaded from UCSC and used for calculating the number of SINEs and LINEs in the 200kb region around the TSS. We also evaluated the abundance of several SINE and LINE subtypes by plotting the number of each subtype in a 1Mb region around the TSS. LINE subtypes were organized by evolutionary age based on the youngest predicted age of the corresponding repeat masker classification in (Sookdeo et al., 2013). Finally, the number of full-length and shorter LINE elements in the 200kb region around the TSS were evaluated by

selecting LINEs <6 Kbp or LINEs ≥6 Kbp, respectively. Significant differences between gene classes were tested using a two-sided Mann-Whitney test with α < 0.05.

To evaluate ChIP-seq enrichment around the TSS of the different gene groups, several publicly available ChIP-seq datasets from ESC-derived female neural progenitor cells were reanalysed (CTCF, H3K4me3, H3K27me3 and RAD21 from GSE99991). In short, reads were mapped to the N-masked reference genome generated by SNPsplit based on the SNPs between the C57BL_6NJ and Cast/Ei genomes using bowtie2 v2.4.1 (Kim et al., 2015; Langmead and Salzberg, 2012). SNPsplit was then used to assign the reads to either the C57BL_6NJ or Cast/Ei bam file based on the best alignment or to a common bam file if mapping to a region without allele-specific SNPs. The allele-specific and unassigned bam files were sorted using samtools v1.10 (Li et al., 2009). Finally, the allele-specific bam files were normalized based on the total number of mapped reads per sample. The scaling factor was calculated as 10^6 / total number of mapped reads and used as parameter --scaleFactor using deepTools bamCoverage v3.5.0 (--extendReads --binSize 1). Replicates were merged using using WiggleTools v1.2.3 (Zerbino et al., 2014). Xist CHART-seq data from (Wang et al., 2018) was downloaded and lift over from mm9 to mm10 using CrossMap v0.5.2 (Zhao et al., 2014). Allele-specific ChIP-seq density ± 3kb around the TSS of the different gene groups was visualized using deepTools plotProfile v3.5.0 (Ramírez et al., 2016). Moreover, the overlap of the CHART-seq data with the gene body was plotted using deepTools plotProfile showing 3kb upstream of the TSS to 3kb downstream of the TES.Hi-C data from female NPCs was downloaded from (Bauer et al., 2021) (GSE157448). The allele-specific Hi-C matrices were corrected using HiCExplorer v3.6 (Ramírez et al., 2018) hicCorrectMatrix with ICE as correction method and a lower and upper threshold of -1.4 and 2, respectively. The Xi and Xa Hi-C data was visualized using pyGenomeTracks (Lopez-Delisle et al., 2020). The TAD boundaries were downloaded from Bonev et al 2017. For each TAD on the X-chromosome, the number of overlapping genes, reactivated genes and escapees were counted using bedtools intersect. TADs with significant more or less reactivated genes were selected using a Binomial test based on the ratio between the number of reactivated genes and the total number of genes for the whole X-chromosome (p-value < 0.05). The tracks showing the SINE, LINE and CpG density, the CHART-seq track and the allele-specific tracks from CTCF, RAD21, H3K4me3 and H3K27me3 were added. Differences between TADs were plotted in violin plots by

comparing the TADs with significantly more reactivated genes, significantly less reactivated genes and non-significant TADs. For each TAD, the number of overlapping CpG sites, SINEs and LINEs were counted using bedtools intersect and normalized for the TAD length. Moreover, the number of overlapping reads from the CHART-seq and allele-specific ChIP-seq data was counted using bedtools intersect and normalized for the TAD length.

MeD-seq

MeD-seq analyses were essentially carried out as previously described (Boers et al., 2018). In brief: DNA samples were digested by *LpnPI* (New England Biolabs, Ipswich, MA, USA), resulting in snippets of 32 bp around a fully-methylated recognition site that contains a CpG. These short DNA fragments were further processed using a ThruPlex DNA–seq 96D kit (cat#R400407, Rubicon Genomics Ann Arbor, MI, USA) and a Pippin system. Stem-loop adapters were blunt-end ligated to repaired input DNA and amplified to include dual indexed barcodes using a high-fidelity polymerase to generate an indexed Illumina NGS library. The amplified end product was purified on a Pippin HT system with 3% agarose gel cassettes (Sage Science, Beverly, MA, USA). Multiplexed samples were sequenced on Illumina HiSeq2500 systems for single reads of 50 bp according to the manufacturer's instructions. Dual indexed samples were demultiplexed using bcl2fastq software (Illumina, San Diego, CA, USA). Data processing was carried out using custom scripts in Python. Raw fastq files were subjected to Illumina adaptor trimming and reads were filtered based on *LpnPI* restriction site occurrence between 13-17 bp from either 5' or 3' end of the read. Reads that passed the filter were mapped to mm10 using. For each *LpnPI* site, the number of overlapping reads were counted and normalized for the sequencing depth. We defined the TSS region as the region ± 1kb of the TSS and generated read count scores for the TSS region of each gene. Differentially methylated TSS regions were detected using a Mann-Whitney test on the normalized read counts of the High samples and the control samples.

For each gene, the ratio between high and control was calculated by dividing the normalized number of reads overlapping the TSS region in the high samples by the those overlapping the TSS region in the control samples. Only genes with more than 10 reads overlapping the TSS region across all samples were used. The ratios between the genes of the

different gene classes were compared using a violin plot showing the ratios per group. Methylation differences between the TSS region of reactivated and non-reactivated genes were explored by plotting the methylation profiles for both genes in a heatmap. For each gene, the normalized number of reads overlapping the TSS region were converted to z-scores for plotting. The genes were clustered based on the Euclidean distance and annotated as either reactivated or non-reactivated to reveal clustering differences between both groups.

Demethylation of SINEs and LINEs was examined by selecting SINEs and LINEs in the 200kb region around the TSSs of reactivated and non-reactivated genes. We counted the number of MeD-seq reads in the 400bp region around the SINEs and LINEs and normalized this number for sequencing depth using the total number of mapped MeD-seq reads per sample. For each repeat element, the ratio between high and control was calculated by dividing the normalized number of reads in the high samples by the those in the control samples. The methylation ratios of the SINEs and LINEs close to genes from the different gene classes were visualized in violin plots.

For the genome browser overviews of the MeD-seq samples, the bam files were normalized using deepTools bamCoverage v3.5.0 (Ramírez et al., 2016) with CPM as normalization method and a bin size of 1. For each condition, the tracks of the replicates were merged using WiggleTools v1.2.3 (Zerbino et al. 2014).

Harmony image analysis

Neurons were processed as per the IF protocol described above. Images were acquired with an Opera Phenix confocal microscope (PerkinElmer) and analyzed with a Harmony software (v4.9, PerkinElmer). Nuclei were determined with DAPI while TUJ-1-Alexa488-positive cells were selected and further analyzed for nuclear area, number of extremities per nucleus and number of roots per nucleus.

Supplemental References

Anders, S., Pyl, P.T., and Huber, W. (2015). HTSeq—a Python framework to work with high-throughput sequencing data. Bioinformatics *31*, 166–169.

Bauer, M., Vidal, E., Zorita, E., Üresin, N., Pinter, S.F., Filion, G.J., and Payer, B. (2021). Chromosome compartments on the inactive X guide TAD formation independently of transcription during X-reactivation. Nat Commun *12*, 3499.

Boers, R., Boers, J., Hoon, B. de, Kockx, C., Ozgur, Z., Molijn, A., IJcken, W. van, Laven, J., and Gribnau, J. (2018). Genome-wide DNA methylation profiling using the methylation-dependent restriction enzyme LpnPI. Genome Res *28*, 88–99.

Carrette, L.L.G., Wang, C.-Y., Wei, C., Press, W., Ma, W., Kelleher, R.J., and Lee, J.T. (2018). A mixed modality approach towards Xi reactivation for Rett syndrome and other X-linked disorders. Proceedings of the National Academy of Sciences of the United States of America *115*, E668–E675.

Csankovszki, G., Panning, B., Bates, B., Pehrson, J.R., and Jaenisch, R. (1999). Conditional deletion of Xist disrupts histone macroH2A localization but not maintenance of X inactivation. Nature Genetics *22*, 323–324.

Guy, J., Hendrich, B., Holmes, M., Martin, J.E., and Bird, A. (2001). A mouse Mecp2-null mutation causes neurological symptoms that mimic Rett syndrome. Nature Genetics *27*, 322–326.

Janiszewski, A., Talon, I., Chappell, J., Collombet, S., Song, J., Geest, N.D., To, S.K., Bervoets, G., Marin-Bejar, O., Provenzano, C., et al. (2019). Dynamic reversal of random X-Chromosome inactivation during iPSC reprogramming. Genome Research *29*, 1659–1672.

Keane, T.M., Goodstadt, L., Danecek, P., White, M.A., Wong, K., Yalcin, B., Heger, A., Agam, A., Slater, G., Goodson, M., et al. (2011). Mouse genomic variation and its effect on phenotypes and gene regulation. *477*, 289–294.

Kim, D., Langmead, B., and Salzberg, S.L. (2015). HISAT: a fast spliced aligner with low memory requirements. Nat Methods *12*, 357–360.

Krueger, F., and Andrews, S.R. (2016). SNPsplit: Allele-specific splitting of alignments between genomes with known SNP genotypes. F1000research *5*, 1479.

Langmead, B., and Salzberg, S.L. (2012). Fast gapped-read alignment with Bowtie 2. Nat Methods *9*, 357–359.

Li, H., Handsaker, B., Wysoker, A., Fennell, T., Ruan, J., Homer, N., Marth, G., Abecasis, G., Durbin, R., and Subgroup, 1000 Genome Project Data Processing (2009). The Sequence Alignment/Map format and SAMtools. Bioinformatics *25*, 2078–2079.

Lopez-Delisle, L., Rabbani, L., Wolff, J., Bhardwaj, V., Backofen, R., Grüning, B., Ramírez, F., and Manke, T. (2020). pyGenomeTracks: reproducible plots for multivariate genomic data sets. Bioinformatics *37*, btaa692-.

Love, M.I., Huber, W., and Anders, S. (2014). Moderated estimation of fold change and dispersion for RNA-seq data with DESeq2. Genome Biol *15*, 550.

Quinlan, A.R., and Hall, I.M. (2010). BEDTools: a flexible suite of utilities for comparing genomic features. Bioinformatics *26*, 841–842.

Ramírez, F., Ryan, D.P., Grüning, B., Bhardwaj, V., Kilpert, F., Richter, A.S., Heyne, S., Dündar, F., and Manke, T. (2016). deepTools2: a next generation web server for deep-sequencing data analysis. Nucleic Acids Res *44*, W160–W165.

Ramírez, F., Bhardwaj, V., Arrigoni, L., Lam, K.C., Grüning, B.A., Villaveces, J., Habermann, B., Akhtar, A., and Manke, T. (2018). High-resolution TADs reveal DNA sequences underlying genome organization in flies. Nat Commun *9*, 189.

Sookdeo, A., Hepp, C.M., McClure, M.A., and Boissinot, S. (2013). Revisiting the evolution of mouse LINE-1 in the genomic era. Mobile Dna-Uk *4*, 3–3.

Spiliotopoulos, D., Goffredo, D., Conti, L., Febo, F.D., Biella, G., Toselli, M., and Cattaneo, E. (2009). An optimized experimental strategy for efficient conversion of embryonic stem (ES)-derived mouse neural stem (NS) cells into a nearly homogeneous mature neuronal population. Neurobiol Dis *34*, 320–331.

Wang, C.-Y., Jégu, T., Chu, H.-P., Oh, H.J., and Lee, J.T. (2018). SMCHD1 Merges Chromosome Compartments and Assists Formation of Super-Structures on the Inactive X. Cell *174*, 406-421.e25.

Yeh, H.-W., Karmach, O., Ji, A., Carter, D., Martins-Green, M.M., and Ai, H. (2017). Red-shifted luciferase–luciferin pairs for enhanced bioluminescence imaging. Nat Methods *14*, 971–974.

Zerbino, D.R., Johnson, N., Juettemann, T., Wilder, S.P., and Flicek, P. (2014). WiggleTools: parallel processing of large collections of genome-wide datasets for visualization and statistical analysis. Bioinformatics *30*, 1008–1009.

Zhao, H., Sun, Z., Wang, J., Huang, H., Kocher, J.-P., and Wang, L. (2014). CrossMap: a versatile tool for coordinate conversion between genome assemblies. Bioinformatics *30*, 1006–1007.
