# Supplementary material for: Genetic and epigenetic determinants of reactivation of Mecp2 and the inactive X chromosome in neural stem cells
Source: Stem Cell Reports. 2022 Feb 10;17(3):693–706. doi: 10.1016/j.stemcr.2022.01.008 (PMC9039756; doi:10.1016/j.stemcr.2022.01.008)
Supplement: Document S2. Article plus supplemental information [file mmc4.pdf]

Stem Cell Reports
Resource
OPEN ACCESS

Genetic and epigenetic determinants of reactivation of Mecp2 and the inactive X chromosome in neural stem cells

H. Mira-Bontenbal,[1,6,*] B. Tan,[1,6] C. Gontan,[1] S. Goossens,[1] R.G. Boers,[1] J.B. Boers,[1] C. Dupont,[1] M.E. van Royen,[2] W.F.J. IJcken,[3] P. French,[4] A. Bedalov,[5] and J. Gribnau[1,*]
[1]Oncode Institue, Department of Developmental Biology, Erasmus MC, Rotterdam, the Netherlands
[2]Department of Pathology, Erasmus MC, Rotterdam, the Netherlands
[3]Center for Biomics, Department of Cell Biology, Erasmus MC, Rotterdam, the Netherlands
[4]Department of Neurology, Erasmus MC Cancer Institute, University Medical Center, Rotterdam, the Netherlands
[5]Clinical Research Division, Fred Hutchinson Cancer Research Center, 1100 Fairview Avenue N., Seattle, WA 98109, USA
[6]These authors contributed equally
*Correspondence: h.mirabontenbal@erasmusmc.nl (H.M.-B.), j.gribnau@erasmusmc.nl (J.G.)
https://doi.org/10.1016/j.stemcr.2022.01.008

SUMMARY

Rett syndrome may be treated by reactivating the silent copy of *Mecp2* from the inactive X chromosome in female cells. Most studies that model *Mecp2* reactivation have used mouse fibroblasts rather than neural cells, which would be critical for phenotypic reversal, and rely on fluorescent reporters that lack adequate sensitivity. Here, we present a mouse model based on a dual bioluminescent and fluorescent reporter to assess the level of reactivation of *Mecp2* and the inactive X chromosome by treating neural stem cells with 5-azacytidine and *Xist* knockdown. We show that reactivation of *Mecp2* and other X-linked genes correlates with CpG density, with distance from escapees, and, very strongly, with the presence of short interspersed nuclear elements. In addition, X-linked genes reactivated in neural stem cells overlap substantially with early reactivating genes by induced pluripotent stem cell reprogramming of fibroblasts or neuronal progenitors, indicating that X chromosome reactivation follows similar paths regardless of the technique or cell type used.

INTRODUCTION

Rett syndrome (RTT) is the second most prevalent cause of intellectual disability in girls after Down syndrome, affecting 1 in 10,000 live female births (Weaving et al., 2005). It is caused by heterozygous mutations in the methyl-CpG-binding protein 2 (MECP2), whose gene is X linked and subject to random X chromosome inactivation (XCI) during early embryogenesis. RTT-affected girls are thus mosaic in terms of *MECP2* expression: half of their cells will express the wild-type (WT) copy of *MECP2*, while the other half will express the mutant *MECP2* allele. This also implies that RTT-affected cells have a silenced WT *MECP2* copy located on the inactive X chromosome (Xi). Previous work has shown that postnatal re-expression of WT *Mecp2* copies in an RTT mouse model causes its phenotype to revert (Giacometti et al., 2007; Guy et al., 2007), which has sparked major interest in the RTT field in re-expressing WT MECP2 in human RTT patients. One way of achieving this is by reactivation of the endogenous WT copy of *MECP2* on the Xi in RTT cells.

In mice, XCI is initiated early in pre-implantation development, where at the eight-cell stage the paternally inherited X is inactivated (Okamoto et al., 2004). Subsequently, the inactive X is reactivated in the inner cell mass (ICM), followed by random XCI of either the maternally or the paternally inherited X. From there on, the inactive state is inherited by all daughter cells, and only in the developing oocyte is the inactive X reactivated (Mak et al., 2004). Hence, cells in the ICM of the female mouse blastocyst and female embryonic stem cells (ESCs) bear two active X chromosomes. Upon development and epiblast formation or ESC differentiation, one of the X chromosomes is randomly chosen to upregulate expression of the long non-coding RNA *Xist* (Monkhorst et al., 2008). This results in the coating of a single X chromosome with *Xist* and recruitment of proteins such as SPEN, RBM15, HDAC3, and the polycomb repressive complexes PRC1 and PRC2 to silence X-linked genes in *cis* (Chu et al., 2015; Fang et al., 2004; McHugh et al., 2015; Minajigi et al., 2015; Moindrot et al., 2015; Monfort et al., 2015; Napoles et al., 2004; Plath et al., 2004). Eventually, CpGs at promoters become methylated to lock XCI down (Gendrel et al., 2013).

Several studies have delved into the mechanics of *Mecp2* reactivation or, in more general terms, X chromosome reactivation (XCR) in mouse cells and tissues by looking for factors that are important in maintaining *Xist* expression, by directly knocking down *Xist*, or by inhibiting the DNA methyltransferase DNMT1 (Adrianse et al., 2018; Bhatnagar et al., 2014; Carrette et al., 2018; Lessing et al., 2016; Przanowski et al., 2018; Sripathy et al., 2017). The combination of *Xist* knockdown using short hairpin RNA (shRNA) or antisense oligonucleotides (ASOs) with 5-azacytidine (5-Aza; a DNMT1 inhibitor) treatment

This is an open access article under the CC BY license (http://creativecommons.org/licenses/by/4.0/).

synergistically reactivated *Mecp2* fused to a firefly luciferase reporter on the Xi of a mouse fibroblast cell line (Carrette et al., 2018; Sripathy et al., 2017). In addition, blocking the PI3K/AKT/mTor pathway using inhibition of SGK1, a downstream effector of PDPK1, or mTOR with GSK650394 or rapamycin, respectively, resulted in biallelic expression of *Mecp2* in mouse fibroblasts, while inhibition of ACVR1 with LDN193189 led to similar results (Przanowski et al., 2018). Treatment of fibroblasts carrying a GFP transgene on the Xi with rapamycin, GSK650394, or LDN193189 led to increased fluorescence (Przanowski et al., 2018), confirming that the PI3K/AKT/mTOR and BMP pathways are involved in maintenance of repression of the Xi. *In vivo*, injection of GSK650394 and LDN193189 into brains of *Xist*$^{-/+}$:*Mecp2*$^{+/GFP}$ mice where *Mecp2* is fused to GFP on the Xi resulted as well in significant GFP expression (Przanowski et al., 2018). Additional studies also showed that inhibition of DNMT1 and Aurora kinases results in synergistic reactivation of an Xi-linked GFP transgene (Lessing et al., 2016). A more suitable approach to performing high-throughput chemical compound screens for *Mecp2* reactivation requires the generation of an improved mouse model, the derivation of its associated cell lines closer to the neuronal target cells, and the use of a highly sensitive luciferase, instead of fluorescence, whose expression is under the control of the endogenous *Mecp2* promoter and not a transgene on the X chromosome.

Here, we have developed a mouse model system where *Mecp2* is fused to NLuc, a luciferase enzyme smaller and 100 times brighter than the regular firefly luciferase. We have also introduced a fluorescent TdTomato reporter downstream of NLuc separated by a P2A signal. This dual capability permits not only measurement of NLuc activity at a populational level, but also measurement of Tomato fluorescence at the single-cell level. Our *Xist*$^{-/+}$:*Mecp2*$^{+/NLucTom}$ compound mice display complete skewed XCI of the reporter allele and are generated in a highly polymorphic C57BL/6:Cast/Eij (maternal:paternal) F1 hybrid background, providing a wealth of SNPs for X-chromosome-wide allele-specific expression analysis. From these mutant mice, we have isolated mouse embryonic fibroblasts (MEFs), ESCs, and neural stem cells (NSCs) for further studies and to provide them to the community. We show that 5-Aza treatment in combination with *Xist* knockdown in NSCs leads to XCR with a striking resemblance to induced pluripotent stem cell (iPSC)-reprogramming-specific XCR (Janiszewski et al., 2019; Bauer et al., 2021), suggesting a general pattern in the capability of X-linked genes to reactivate independent of the mechanism or cell type. In this article, we highlight the potential of our model to study XCR.

RESULTS

Generation of *Mecp2-NanoLuciferase-TdTomato* mice

To obtain highly polymorphic *Xist*$^{-/+}$:*Mecp2*$^{+/NLucTom}$ mice, we first generated *Mecp2*$^{NLucTom/Y}$ ESCs in a Cast/EiJ (cast) background. We transfected WT male cast ESCs with the *NanoLuciferase-P2A-TdTomato* (NLucTom) construct, where NLuc is fused to the C terminus of *Mecp2* and TdTomato (Tomato from here on) is translated as an independent protein, thanks to a P2A self-cleaving peptide (Figure 1A). Fluorescence-activated cell sorting (FACS) analysis showed a distinct Tomato-positive cell population that was sorted and expanded (Figure 1B). PCR analysis using primers spanning the 5′- and 3′-specific integration sites and primers against the endogenous allele confirmed proper integration on DNA obtained from sorted Tomato-positive cells (Figures 1A and 1C, Table S1). This resulted in the appearance of a higher-molecular-weight band of MECP2 by immunoblotting owing to its fusion to NLuc (19 kDa, Figure 1D). Luminescence analysis showed very strong NLuc activity in *Mecp2*$^{NLucTom/Y}$ ESCs compared with WT ESCs (Figure 1E). Cells were then injected into blastocysts and a cast colony of *Mepc2-NLucTom* mice was generated. *Mecp2*$^{NLucTom/Y}$ mice are viable with normal lifespan and do not show any RTT-related phenotype, indicating that the fusion of NLuc to MECP2 is not deleterious to its function (Figure S1A). Immunofluorescence (IF) for NLuc and Tomato fluorescence analysis in a *Mecp2*$^{+/LucTom}$ fully cast female brain shows that MECP2-NLuc and Tomato are expressed in 45% of the cells, as expected from random XCI (Figure S1B). Moreover, *Mecp2*$^{+/LucTom}$ female brains also show high NLuc activity compared with WT controls, highlighting the usefulness of this system for *in vivo* studies (Figure S1C). We have thus generated a *Mecp2-NLucTom* mouse colony in a cast background.

Generation of *Xist*$^{-/+}$:*Mecp2*$^{+/NLucTom}$, *Xist*$^{-/+}$:*Mecp2*$^{-/NLucTom}$, *Mecp2*$^{+/NLucTom}$, and *Mecp2*$^{NLucTom/NLucTom}$ cell lines

To study *Mecp2* reactivation, we crossed cast *Mecp2*$^{NLucTom/Y}$ males with C57BL/6 (Bl6) WT, *Zp3-Cre:Xist*$^{+/2lox}$, or *Zp3-Cre:Xist*$^{+/2lox}$:*Mecp2*$^{+/2lox}$ females (Figure S2A). Oocyte-specific expression of Cre, thanks to the *Zp3-Cre* transgene, results in recombination of *loxP* sites before fertilization, resulting in female embryos that are *Xist*$^{-/+}$:*Mecp2*$^{+/NLucTom}$ and *Xist*$^{-/+}$:*Mecp2*$^{-/NLucTom}$ (among other genotypes). In this way, we isolated Bl6:cast WT control, *Xist*$^{-/+}$:*Mecp2*$^{+/NLucTom}$, *Xist*$^{-/+}$:*Mecp2*$^{-/NLucTom}$, *Mecp2*$^{+/NLucTom}$, and cast *Mecp2*$^{NLucTom/NLucTom}$ MEF, ESC, and NSC lines. Genotyping of the F1 NSCs confirmed proper integration of the NLuc-Tomato cassette at the *Mecp2* locus in mice (Figure S2B). We confirmed by IF SOX2 expression and

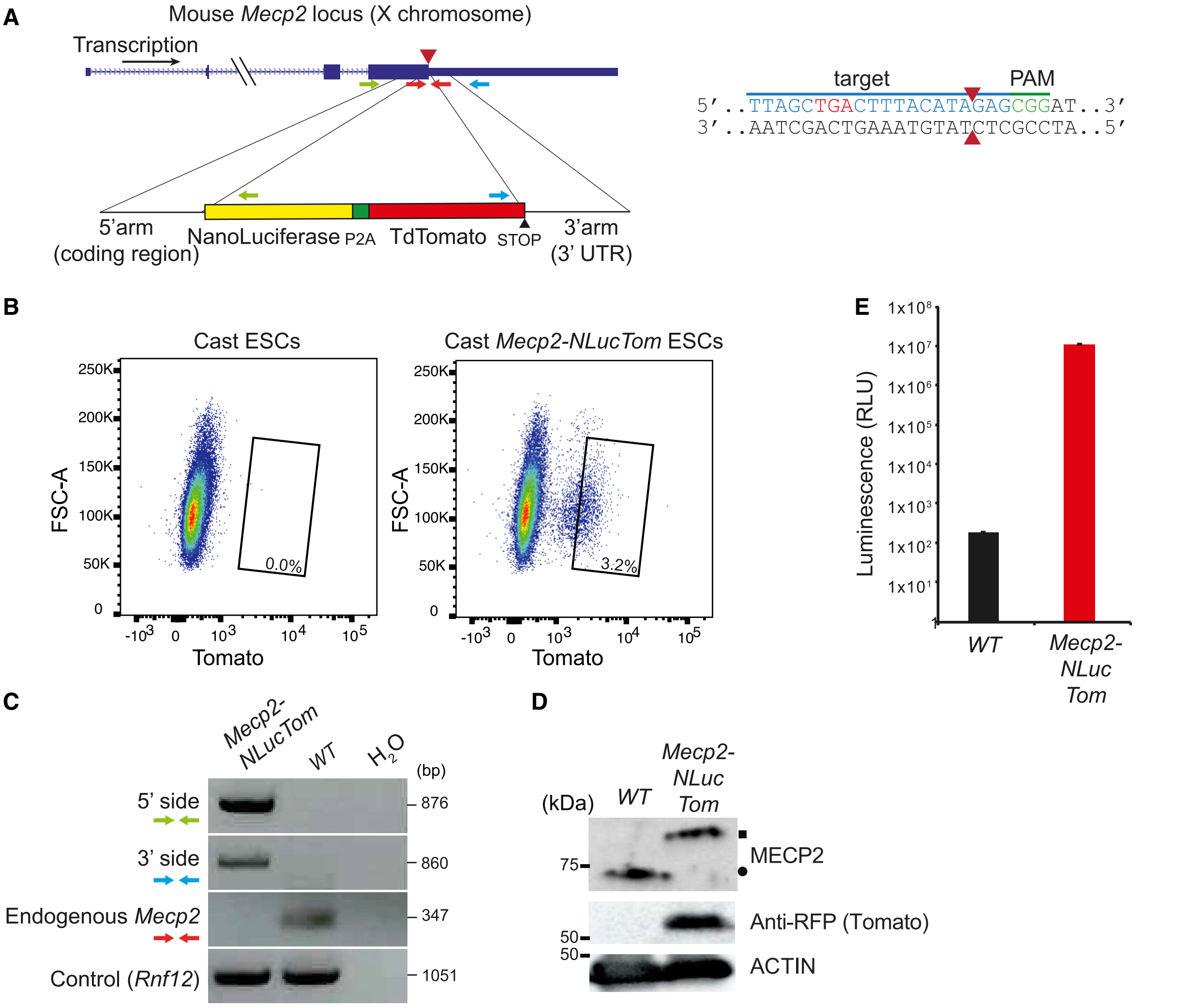

Figure 1. $Mecp2^{NLucTom/Y}$ male cast ESCs show proper reporter integration and expression

(A) Mouse *Mecp2* locus with the NLuc and Tomato donor vector. Green, blue, and red primer sets were used to amplify the 5′ integration site (forward primer outside the 5′ homology arm, inside the coding region, and reverse primer inside NLuc), the 3′ integration site (forward primer in Tomato and reverse primer outside of 3′ homology arm), and the non-targeted endogenous end of *Mecp2*, respectively (see [C]). The guide RNA, PAM, cutting site (red arrowheads), and *Mecp2*'s TGA STOP codon are depicted on the right. Primer sequences are found in Table S1.

(B) FACS plots depicting Tomato fluorescence before and after transfection of WT male cast ESCs with CRISPR-Cas9 and the donor vector depicted in (A). The rectangle shows the sorted population, 3.2% of the total live population.

(C) Genomic PCR with primers described in (A) on FACS-sorted $Mecp2^{NLucTom/Y}$ cast ESCs and parental WT ESCs. A control locus PCR band is depicted (*Rnf12*).

(D) Western blot analysis of FACS-sorted $Mecp2^{NLucTom/Y}$ ESCs and parental WT ESCs. Tomato is translated as an independent protein, thanks to the P2A signal. Loading control, actin. MECP2-NLuc and WT MECP2 are indicated by a square and a circle, respectively.

(E) NLuc activity assay of FACS-sorted $Mecp2^{NLucTom/Y}$ ESCs and parental WT ESCs (500,000 cells analyzed per well, average activity ± SD, n = 3 independent biological replicates).

absence of the differentiated neuron-specific marker TUJ-1 in our NSC lines (Figure S2C). Our NSC lines were also able to differentiate into TUJ-1-expressing neurons, GFAP-expressing astrocytes, and OLIG2-expressing oligodendrocytes, confirming their stemness (Figure 2A). However, *in vitro* grown neurons with RTT ($Xist^{-/+}$:$Mecp2^{-/NLucTom}$) did not show any differences compared with WT or $Xist^{-/+}$:$Mecp2^{+/NLucTom}$ neurons in terms of nuclear size, number of roots and extremities per nucleus, or total neurite length per nucleus (Figure S2D).

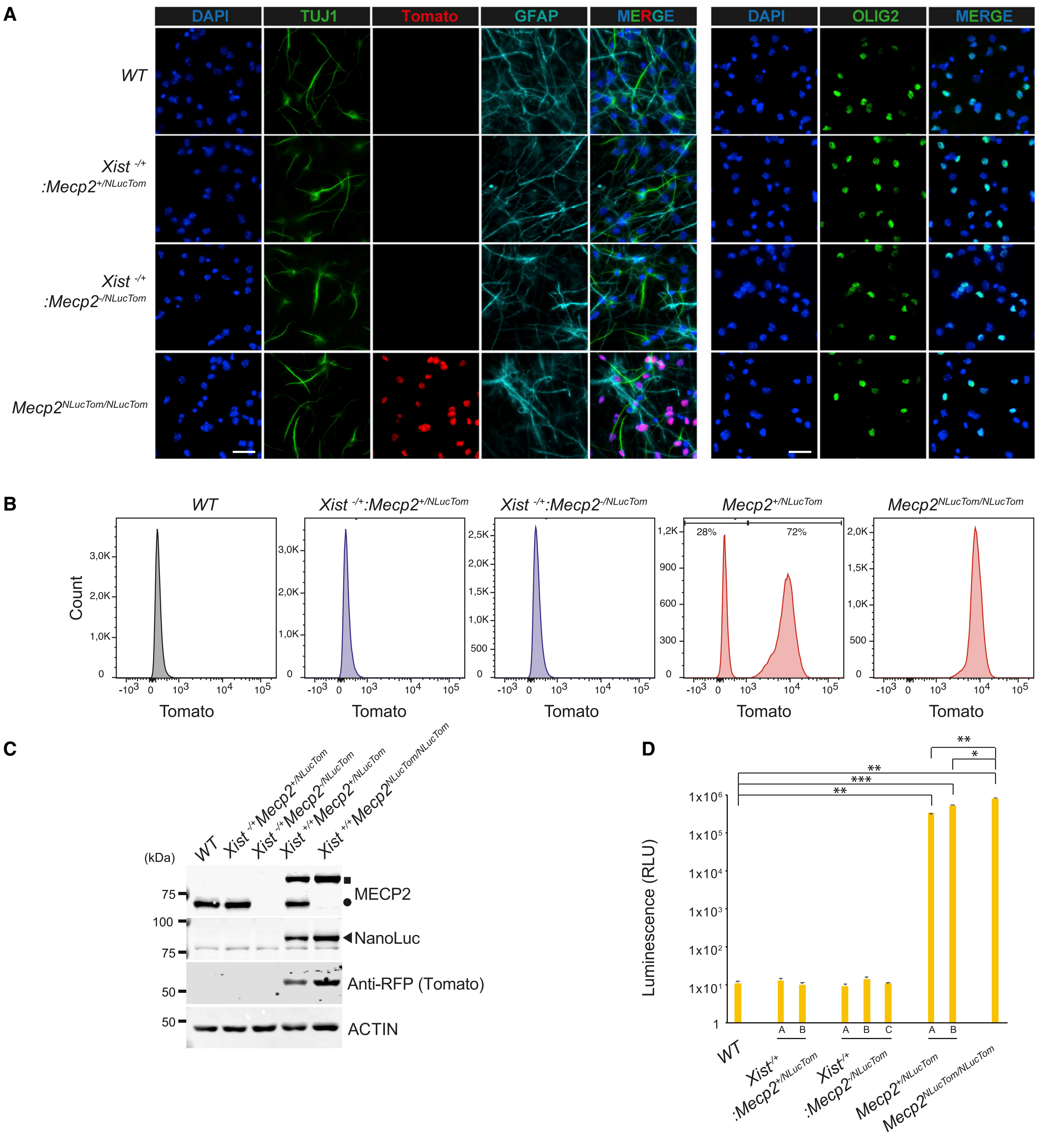

Figure 2. Characterization of *Xist*$^{-/+}$:*Mecp2*$^{+/NLucTom}$, *Xist*$^{-/+}$:*Mecp2*$^{-/NLucTom}$, *Mecp2*$^{+/NLucTom}$, and *Mecp2*$^{NLucTom/NLucTom}$ NSCs

(A) IF of TUJ1 (green, left), GFAP (turquoise), and OLIG2 (green, right) of WT, *Xist*$^{-/+}$:*Mecp2*$^{+/NLucTom}$, *Xist*$^{-/+}$:*Mecp2*$^{-/NLucTom}$, and *Mecp2*$^{NLucTom/NLucTom}$ NSCs differentiated toward neurons, astrocytes, and oligodendrocytes. Tomato fluorescence was measured directly. Blue, DAPI. White scale bars, 25 μm; n = 1.

(B) FACS analysis of Tomato fluorescence of WT, *Xist*$^{-/+}$:*Mecp2*$^{+/NLucTom}$, *Xist*$^{-/+}$:*Mecp2*$^{-/NLucTom}$, and *Mecp2*$^{NLucTom/NLucTom}$ NSCs. The percentage of Tomato-positive *Mecp2*$^{+/NLucTom}$ NSCs is shown.

(C) Western blot analysis of WT, *Xist*$^{-/+}$:*Mecp2*$^{+/NLucTom}$, *Xist*$^{-/+}$:*Mecp2*$^{-/NLucTom}$, and *Mecp2*$^{NLucTom/NLucTom}$ NSCs showing expression of MECP2-NLuc in *Mecp2*$^{+/NLucTom}$ and *Mecp2*$^{NLucTom/NLucTom}$ NSCs but not in *Xist*$^{-/+}$:*Mecp2*$^{+/NLucTom}$, *Xist*$^{-/+}$:*Mecp2*$^{-/NLucTom}$ NSCs as expected.

(legend continued on next page)

Full skewing of XCI of the paternal cast allele in *Xist*$^{-/+}$:*Mecp2*$^{+/NLucTom}$ and *Xist*$^{-/+}$:*Mecp2*$^{-/NLucTom}$ NSCs was confirmed by FACS analysis (Figure 2B). In addition, *Mecp2*$^{+/NLucTom}$ NSCs displayed skewed XCI as expected from their hybrid origin, where around 60%–70% of the cells were reported to show inactivation of the Bl6 allele (Cattanach and Williams, 1972). *Mecp2*$^{NLucTom/NLucTom}$ NSCs displayed a single Tomato-positive peak. Completely skewed XCI in *Xist*$^{-/+}$:*Mecp2*$^{+/NLucTom}$ and *Xist*$^{-/+}$:*Mecp2*$^{-/NLucTom}$ NSCs and absence of *Mecp2* expression in *Xist*$^{-/+}$:*Mecp2*$^{-/NLucTom}$ NSCs were also demonstrated by immunoblotting analysis (Figure 2C). *Mecp2*$^{+/NLucTom}$ and *Mecp2*$^{NLucTom/NLucTom}$ NSCs showed a higher-molecular-weight band for MECP2-NLuc fusion protein and Tomato expression. Moreover, NLuc activity analysis showed that several *Mecp2*$^{+/NLucTom}$ and *Mecp2*$^{NLucTom/NLucTom}$ NSC clones had high levels of NLuc activity, and as expected, several *Xist*$^{-/+}$:*Mecp2*$^{+/NLucTom}$ and *Xist*$^{-/+}$:*Mecp2*$^{-/NLucTom}$ NSC clones did not (Figure 2D). The background levels of NLuc expression in *Xist*$^{-/+}$:*Mecp2*$^{+/NLucTom}$ and *Xist*$^{-/+}$:*Mecp2*$^{-/NLucTom}$ NSCs were identical to WT cells, indicating that escape of *Mecp2-NLuc* from the inactive cast X chromosome is virtually non-existent from *in vivo*-derived NSCs. To quantify the level of transcriptional repression of *Mecp2-NLuc-Tom* on the Xi, we compared the NLuc activity in cells with the reporter on the active and inactive X. The reporter exhibited a >30,000 times lower level of activity on the Xi compared with the active X chromosome (Figure 2D). Finally, the average NLuc activity arising from two hybrid heterozygous *Mecp2*$^{+/NLucTom}$ clones represented 41% and 65% of the activity of the homozygous *Mecp2*$^{NLucTom/NLucTom}$ clone.

Reactivation of the inactive *Mecp2*-NLuciferase allele

Compounds LDN193189 and GSK650394, which inhibit ACVR1 and SGK1, respectively, have been shown to reactivate an inactive GFP reporter on the Xi in fibroblasts and an inactive *Mecp2-GFP* fusion gene in mouse brains (Przanowski et al., 2018). In addition, the HDAC1/3 inhibitor RG2833 has been shown to facilitate XCR during reprogramming of female Xi-linked GFP transgenic MEFs (Janiszewski et al., 2019).

We therefore treated our NSCs with LDN193189, GSK650394, RG2833, and/or decitabine (structurally very similar to 5-Aza, and called 5-Aza henceforth) for 7 days. Single treatments with LDN193189, GSK650394, or RG2833 and combined treatment with LDN193189 or GSK650394 did not result in *Mecp2* reactivation, however (Figure 3A). Combined treatment of LDN193189 or RG2833 with 5-Aza showed reactivation of the silent NLuc reporter, comparable to single treatment with 5-Aza, indicating that in our hands, 5-Aza is the only tested drug that reactivates the silent copy of *Mecp2*.

Previous work has also shown that 5-Aza treatment in combination with *Xist* knockdown results in XCR in MEFs (Carrette et al., 2018). Therefore, we performed a similar analysis on our *Xist*$^{-/+}$:*Mecp2*$^{+/NLucTom}$ NSCs. Treatment of cells with 0.5 μM 5-Aza for 3 days resulted in a significant 10-fold upregulation of NLuc activity (Figure 3B). If *Xist* was knocked down with ASOs in combination with larger amounts of 5-Aza, reactivation was synergistic and 100-fold higher compared with *Xist* knockdown only, and much higher compared with the background of untreated cells (Figures 3B andS3A). Nevertheless, this reactivation still represented around 0.5%–1% of *Mecp2-NLuc* expression from an active X chromosome in homozygous *Mecp2*$^{NLucTom/NLucTom}$ NSCs.

While NLuc bioluminescence analysis is performed at a populational level, flow cytometry allows us to distinguish Tomato fluorescence at the single-cell level. FACS analysis showed that the entire population of cells shifts toward increased Tomato expression after 10 μM 5-Aza treatment for 3 days (Figure 3C), irrespective of whether *Xist* is knocked down or not. This disagrees with the fact that *Xist* knockdown and 10 μM 5-Aza-treated cells show a 100-fold increase in NLuc activity compared with 10 μM 5-Aza-only-treated cells (Figure 3B), suggesting that NLuc bioluminescence is more sensitive than fluorescence. However, control experiments indicated that the shift of the entire population after 5-Aza treatment toward higher Tomato is due to autofluorescence, since WT female cells equally treated with 5-Aza also show indistinguishable increased Tomato fluorescence (Figure 3D). We noticed, however, a small shoulder on the Tomato-High side of the *Xist* ASO-plus-5-Aza-treated population (Figure 3C). We proceeded to use FACS to sort three independent biological replicates of the Tomato-Low, -Med, and -High populations of *Xist*$^{-/+}$:*Mecp2*$^{+/NLucTom}$ mNSCs treated with *Xist* ASOs and 5-Aza and subsequently performed RNA sequencing (RNA-seq), along with control ASO and non-5-Aza-treated non-FACS-sorted cells (control). We first confirmed by qRT-PCR proper knockdown of *Xist* in all

MECP2-NLuc, WT MECP2, and NLuc are indicated by a square, a circle, and a triangle, respectively. Tomato is expressed as an independent protein.

(D) NLuc activity assay of several clones of WT, *Xist*$^{-/+}$:*Mecp2*$^{+/NlucTom}$, *Xist*$^{-/+}$:*Mecp2*$^{-/NlucTom}$, *Mecp2*$^{+/NlucTom}$, and *Mecp2*$^{NLucTom/NLucTom}$ NSCs, showing an increase of four to five levels of magnitude of NLuc activity from an active X chromosome in *Mecp2*$^{+/NLucTom}$ and *Mecp2*$^{NLucTom/NLucTom}$ NSCs (50,000 cells were analyzed per clone per well, average activity ± SD, n = 3 independent biological replicates). $^{*}p < 0.05$, $^{**}p < 0.01$, $^{***}p < 0.001$, two-tailed Student's t test.

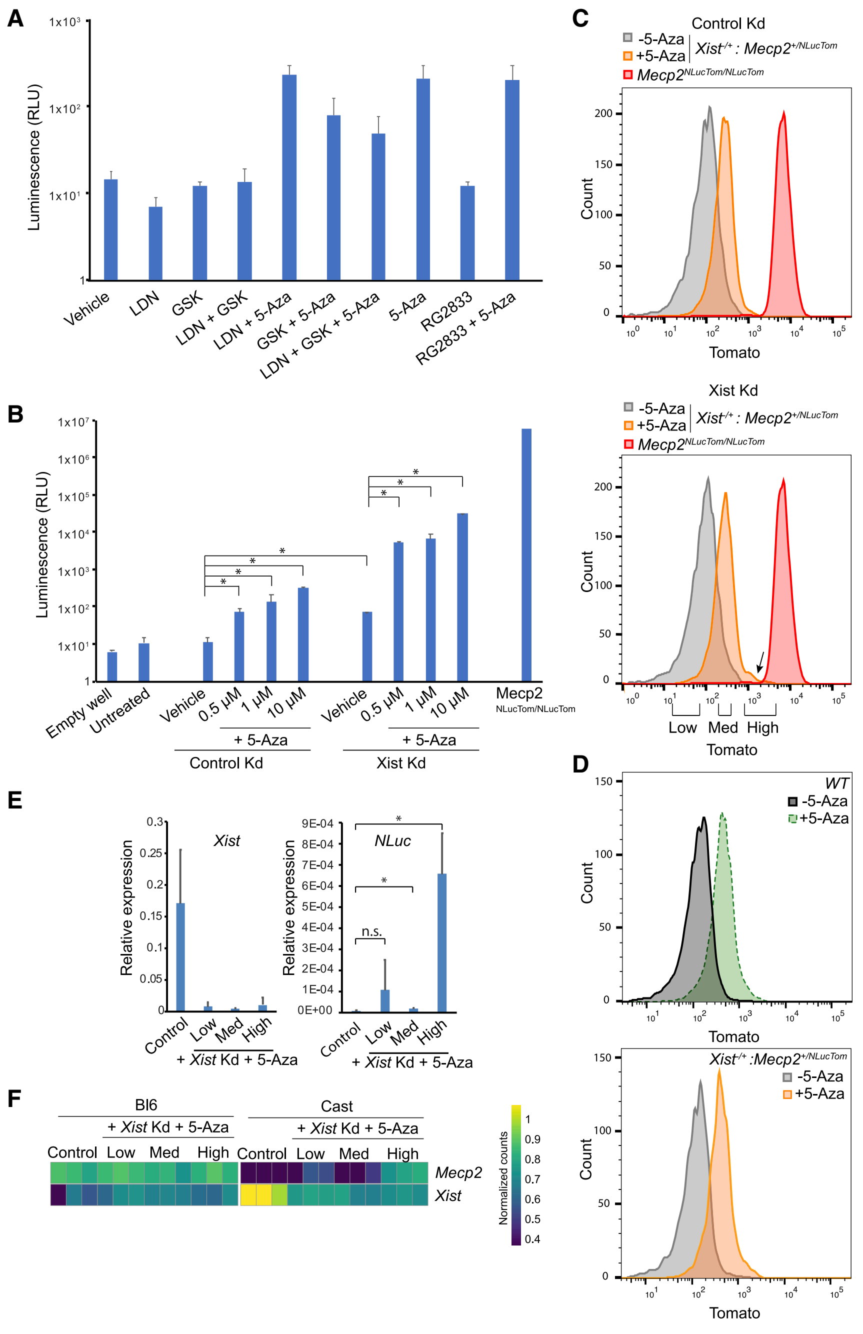

Figure 3. *Xist* knockdown and 5-Aza treatment of *Xist*$^{-/+}$:*Mecp2*$^{+/NLucTom}$ NSCs leads to reactivation of the NLuc-Tomato dual reporter

(A) NLuc activity assay of *Xist*$^{-/+}$:*Mecp2*$^{+/NLucTom}$ NSCs treated with LDN193189 (LDN), GSK650394 (GSK), RG2833, or 5-Aza in different combinations for 7 days (300,000 cells per well, average activity ± SD, n = 3 independent biological replicates).

(B) NLuc activity assay of *Xist*$^{-/+}$:*Mecp2*$^{+/NLucTom}$ NSCs treated with different concentrations of 5-Aza in combination with control ASOs or *Xist* ASOs for 3 days (average activity ± SD, n = 3 independent biological replicates). Significant differences are indicated with an asterisk (500,000 cells were analyzed per well, two-tailed Student's t test, *p < 0.05).

(C) FACS plots depicting *Xist*$^{-/+}$:*Mecp2*$^{+/NLucTom}$ NSCs treated with control or *Xist* ASOs with (orange) or without (gray) 5-Aza for 3 days. *Mecp2*$^{NLucTom/NLucTom}$ NSCs are shown as Tomato-positive controls (red). FACS-sorted populations that were subsequently analyzed by RNA-seq are shown (Low, Med, High). The shoulder in the *Xist* K_d and 5-Aza-treated sample is shown by an arrow; this corresponds to the Tomato-High population; n = 1.

(D) FACS plots depicting WT and *Xist*$^{-/+}$:*Mecp2*$^{+/NLucTom}$ NSCs treated with (dotted green and orange lines, respectively) or without (black and gray, respectively) 5-Aza for 3 days; n = 1.

(E) Relative *Xist* and *NLuc* expression by qRT-PCR analysis in FACS-sorted Tomato-Low, -Med, and -High *Xist*$^{-/+}$:*Mecp2*$^{+/NLucTom}$ NSCs after knockdown of *Xist* and 5-Aza treatment versus non-sorted control cells (average activity ± SD, n = 3 independent biological replicates).

(F) Expression heatmap of *Mecp2* and *Xist* across the different samples and alleles; n = 3 independent biological replicates.

three Tomato populations and significant upregulation of *NLuc* in the Tomato-Med and -High populations (Figure 3E). Among the 2,612 genes on the X chromosome, we obtained sufficient allelic expression information from 447 active genes, of which 45 were classified as escapees, such as previously described *Mid1*, *Eif2s3x*, *Kdm5c*, and *Ddx3x* (Figure S3B and S3C; Yang, 2010; Berletch, 2015). As expected, *Xist* was expressed from the cast Xi and had decreased expression after its knockdown (Figures 3E and 3F). Allele-specific differential expression analysis showed that 86 genes became reactivated from the cast Xi in the Tomato-High population upon *Xist* knockdown and 5-Aza treatment, *Mecp2* included (Figures 3F and 4A). Reactivated genes were seemingly located in a random fashion along the X chromosome, although several clusters were observed (Figure 4B). Among these 86 genes, 7 were more significantly reactivated than *Mecp2* (Figure 4A, Table S2). In addition, *Mecp2* reactivation in the Tomato-Low and -Med populations was not significant by RNA-seq analysis, as expected from the FACS

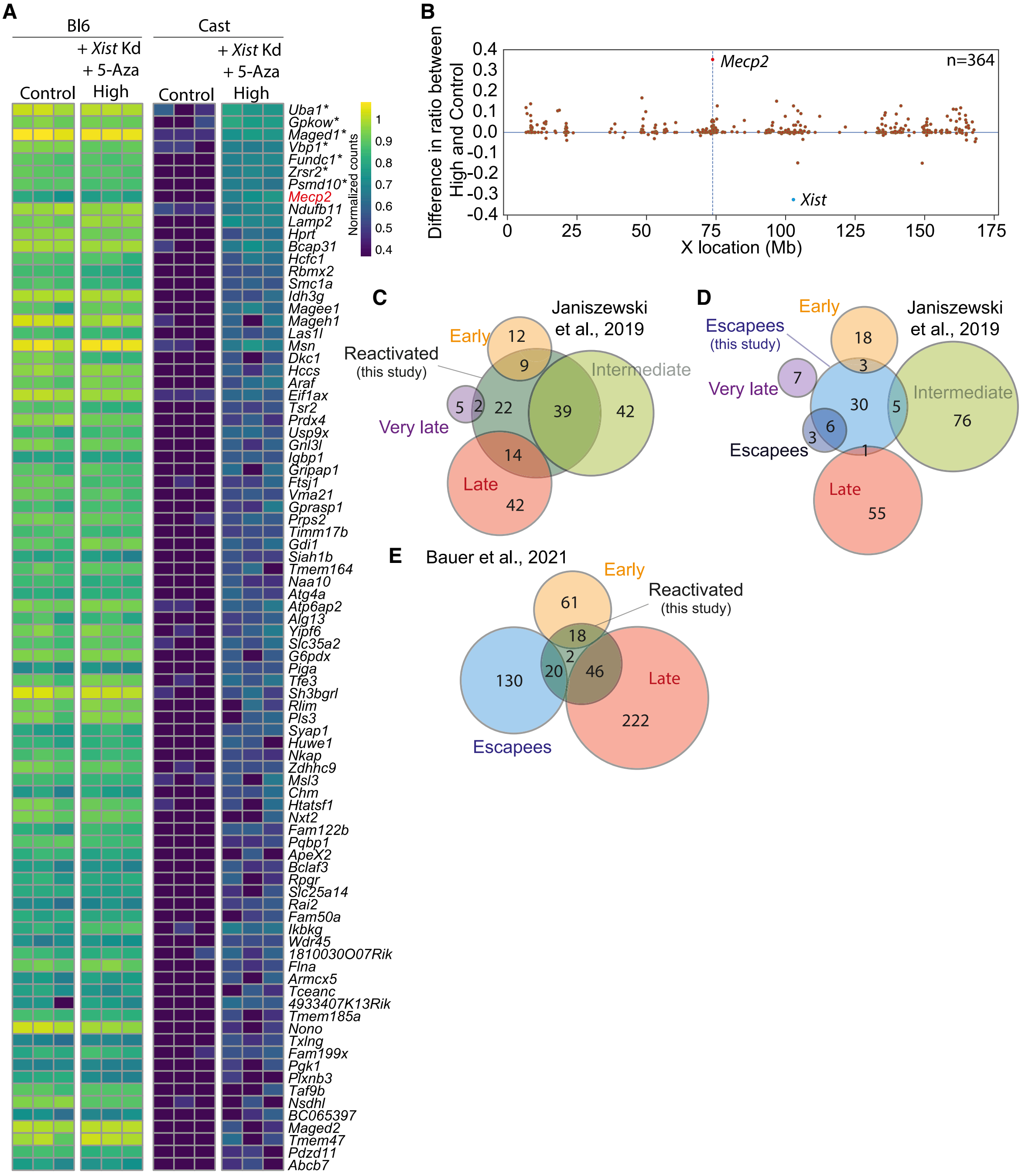

Figure 4. Reactivation of many X-linked genes after *Xist* knockdown and 5-Aza treatment

(A) Heatmap showing the normalized allele-specific counts for the 86 genes that are significantly reactivated from the cast Xi in the FACS-sorted Tomato-High population compared with the negative control sample. *Mecp2* is in red. The seven genes more easily reactivated than *Mecp2* bear an asterisk. n = 3 independent biological RNA replicates that were sequenced for all conditions.

(B) Difference between the ratios of cast expression to total expression (Bl6 + cast) of Tomato-High and control per gene along the X chromosome. Only genes with sufficient allele-specific reads in both conditions are shown.

(C) Venn diagram of the overlap between the reactivated gene list in this study and the early, intermediate, late, or very late reactivated gene subclasses during iPSC reprogramming of MEFs (Janiszewski et al., 2019).

(legend continued on next page)

analysis indicating these populations reflect autofluorescence. However, most of the genes within the 86-gene pool in the Tomato-High population were readily reactivated in the Tomato-Low and -Med populations (in black, Figure S3D, Table S2), while several genes were significantly reactivated only in the Tomato-High population, as was *Mecp2* (in orange, Figure S3D, Table S2). This again suggests that reactivation by *Xist* knockdown and a DNMT1 inhibitor happens more readily for other genes than for *Mecp2*.

In a previous study of iPSC reprogramming of female MEFs, the authors describe different X-linked gene subclasses based on their XCR kinetics, namely early, intermediate, late, and very late reactivation (Janiszewski et al., 2019). We compared our pool of reactivated genes with theirs and observed that 9 and 39 of our genes were among the 21 early (43%) and 81 intermediate (48%) reactivated iPSC genes, respectively (Figure 4C). Similarly, 14/56 (25%) and 2/7 (29%) of our genes were found in their different late and very late reactivation gene subclasses, respectively. This means that a small number of our reactivated genes (22/86, 26%) were not reactivated or were not expressed in the iPSC study. Of note, 6 of their 9 escapees are among our escapee gene pool (67%), while only 9 of their 165 reactivated genes (5%) were in our escapee gene list (Figure 4D), suggesting that our escapee genes are not spuriously reactivated genes, owing to culture conditions, for instance.

XCR kinetics during iPSC reprogramming has also been recently studied in neuronal progenitor cells (NPCs) that were generated through differentiation of ESCs (Bauer et al., 2021). We split their list of reactivated genes into early and late reactivating genes and noticed that 18/79 (23%) and 46/268 (17%) of our genes were found in their early reactivating and late reactivating gene lists, respectively (Figure 4E). Interestingly, we noticed that 20 escapees of their 150-escapee list are genes that were reactivated in our study. This means that 38/86 (44%) of our reactivated genes are either escapees or early reactivating genes in the NPC reprogramming study.

Genomic and epigenomic features of X chromosome reactivation

Since our NSCs were subject to 5-Aza treatment, we investigated whether gene reactivation is dependent on CpG-methylation loss. We first analyzed the density of CpGs in the reactivated and non-reactivated subclasses and found that reactivated genes have significantly more CpGs near their transcription start site (TSS) than non-reactivated genes, but did not show differences in CpG density in their gene bodies (Figures 5A and S4A). We subsequently performed methylated DNA sequencing (MeD-seq) analysis (Boers et al., 2018) on the control and Tomato-High populations to assess the methylation status on the Xi. We additionally analyzed male WT NSCs to assess the methylation status of CpGs on the Xa and in this way be able to infer CpG methylation on the Xi of our female cells. We observed a global decrease in methylation on the X chromosome as expected from the 5-Aza treatment (Figure S4B). However, we surprisingly could not detect a correlation between loss of CpG methylation and reactivation of genes on the Xi (Figure S4B). While male NSCs showed low levels of promoter DNA methylation, as expected from expressed genes on the Xa, reactivated promoters in female cells were not significantly demethylated overall in the Tomato-High population compared with control NSCs, although methylation seemed lower for some of them (Figures S4B and S4C, Table S3). Indeed, 16 of our 86 promoters of reactivated genes showed significantly lower levels of DNA methylation (Figure S4C, Table S3). Nevertheless, cluster analysis of the CpG methylation status of promoters of both reactivated and non-reactivated genes did not result in clustering of reactivated promoters (Figure S4D). Altogether, loss of methylation was not a clear indicator of X-linked gene reactivation, pointing to other mechanisms at play.

We subsequently performed genomic feature correlation analyses on our list of reactivated genes. First, we did not detect a correlation between the position of reactivated genes on the X and proximity to *Xist*, as previously described for X-linked reactivated genes during iPSC reprogramming (Janiszewski et al., 2019) (Figure S5A). However, genes that are reactivated tend to be closer to escapees than non-reactivated genes (Figure 5B), suggesting that proximity to an escapee is a determining factor in the reactivation potential of X-linked genes, as previously described (Bauer et al., 2021; Loda et al., 2017). In addition, we find that genes that are more easily reactivated tend to have significantly fewer long interspersed nuclear elements (LINEs) and more short interspersed nuclear elements (SINEs) around their TSSs (Figure 5C). We found no relationship between specific SINE subclasses and reactivated genes (Figure S5B). We then organized LINEs near genes

(D) Venn diagram of the overlap between the escapees in our study and the early, intermediate, late, very late, and escape gene subclasses during iPSC reprogramming of MEFs (Janiszewski et al., 2019).
(E) Venn diagram of the overlap of the reactivated gene list in this study and the early and late reactivating genes during iPSC reprogramming of NPCs (Bauer et al., 2021). Note that 38/86 (44%) of our reactivated genes are either escapees or early reactivating genes in Bauer et al. (2021).

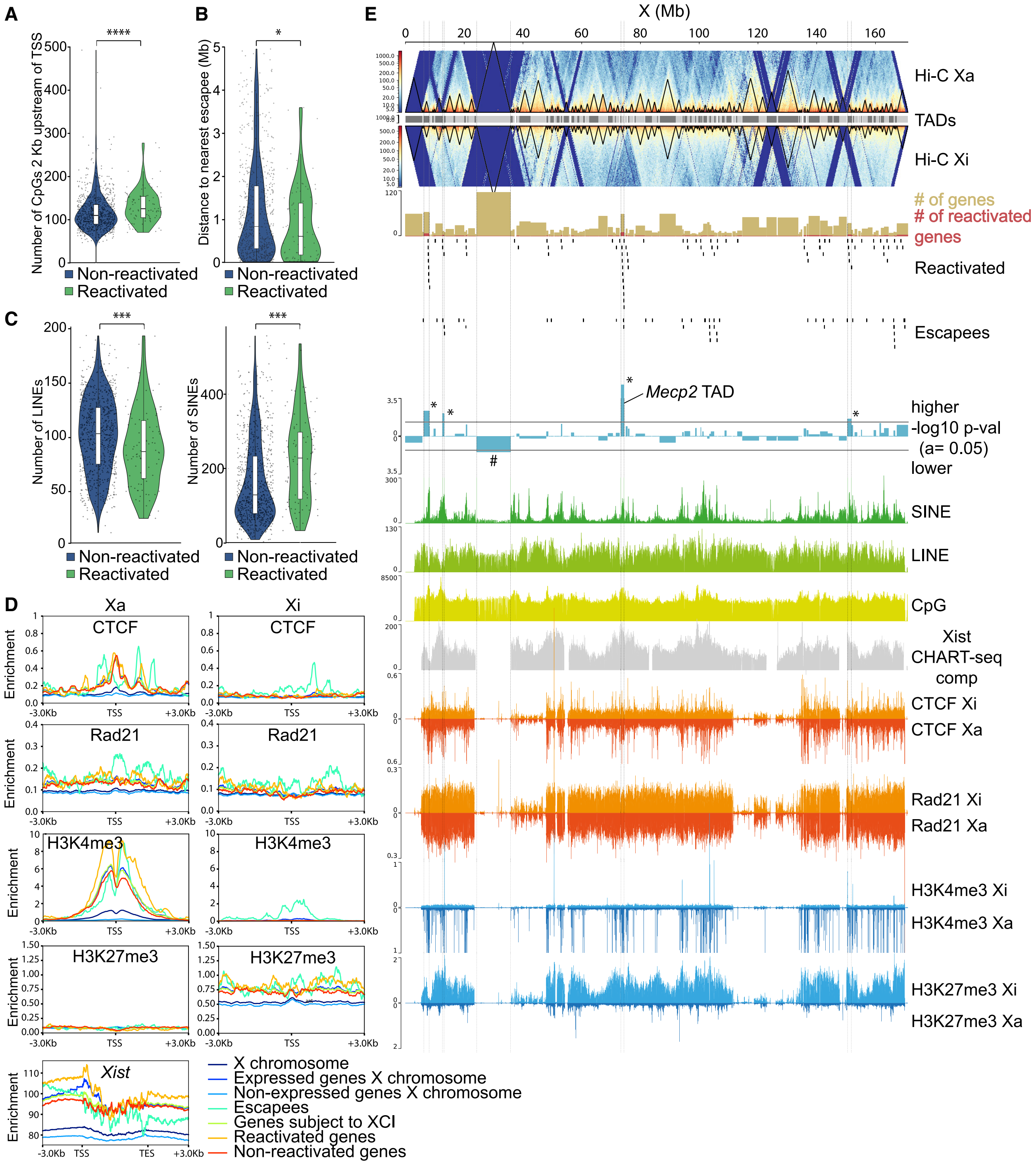

Figure 5. Reactivation of many X-linked genes correlates with genomic and epigenomic features

(A) Violin plot depicting the number of CpGs in a bin of 2 kb upstream of the TSS of non-reactivated (blue) and reactivated (green) genes.****p < 0.0001, Mann-Whitney test.

(B) Violin plots of the distance to the nearest escapee in megabases of non-reactivated (blue) and reactivated genes (green). *p < 0.05, Mann-Whitney test

(legend continued on next page)

by evolutionary age (Figure S5C) (Sookdeo et al., 2013) and size (full-length LINEs of $\geq$6 kb or shorter LINEs) (Figure S5D). Age or size of LINEs in nearby genes is not a predictor of gene reactivation capacity. Finally, we found that DNA flanking SINEs and LINEs close to non-reactivated and reactivated genes shows no difference in methylation levels (Figure S5E). MeD-seq analysis, which is based on 32-bp restriction fragments, prevents direct DNA methylation analysis of LINEs and SINEs owing to their repetitive nature.

We next investigated the correlation between our different gene subclasses with published CTCF and Rad21 binding profiles and several chromatin marks from chromatin immunoprecipitation sequencing (ChIP-seq) datasets obtained from ESC-derived female neural progenitor cells (Wang et al., 2018). Escapees tend to show increased enrichment of CTCF at their TSSs (Figure 5D), as has been previously described (Bonev et al., 2017; Loda et al., 2017). In addition, our reactivated genes tend to have slightly more CTCF binding at their TSSs on the Xa compared with non-reactivated genes, while also bearing increased H3K4me3 deposition on the Xa and very similar H3K27me3 levels on the Xi (Figure 5D). Finally, we also measured the density of *Xist* molecules using a published capture hybridization analysis of RNA targets (CHART)-seq dataset from NPCs (Wang et al., 2018) at promoters of X-linked genes and saw more enrichment of *Xist* at promoters of reactivated genes compared with non-reactivated genes (Figure 5D), in line with what has been previously published for active compartments on the X chromosome (Bauer et al., 2021).

We also examined whether certain topologically associating domains (TADs) are more easily reactivated or prevented from reactivation than others by crossing our gene subclasses with previously published TAD data from NPCs (Bonev et al., 2017). Based on the number of reactivated genes within each TAD, we identified four TADs with significantly more reactivated genes compared with the whole X chromosome (as indicated by an asterisk) (Figure 5E). The TAD containing *Mecp2* shows the significantly largest ratio of reactivated genes, likely because the RNA-seq analysis was performed on Tomato-High (*Mecp2*-reactivated) sorted cells, and suggests that co-activation of nearby genes is limited to the same TAD. Indeed, 9 of the 22 genes that were reactivated in this study, but not by iPSC reprogramming of MEFs (Figure 4C), are located within the *Mecp2* TAD (*Plxnb3*, *Idh3g*, *Naa10*, *Hcfc1*, *Mecp2*, *Flna*, *Gdi1*, *Fam50a*, and *Ikbkg*). Interestingly, these four TADs, significantly enriched with reactivated genes, contain a small number of escapees compared with other non-significant TADs (Figure 5E). In contrast, one TAD is particularly resistant to reactivation (as indicated by #) and yet contains many genes. We subsequently investigated whether the presence of CpGs, SINEs, LINEs, CTCF, Rad21, *Xist*, H3K4me3, and H3K27me3 within those five TADs could be an indicator of their tendency or resistance to reactivate. TADs with significantly more reactivated genes than other TADs tended to have more CpGs and fewer LINEs (although not significant) and contained significantly more SINEs (Figures 5E and S5F). Finally, they also significantly tended to have more CTCF binding and H3K4me3 deposition on the Xa than non-significant TADs and to display more *Xist* accumulation and H3K27me3 deposition, although not significantly, than TADs that are not enriched or depleted for reactivated genes (Figures 5E and S5G). It seems that the active status of genes on the Xa correlates clearly with the reactivation potential of genes.

DISCUSSION

A proper mouse model to study reactivation of *Mecp2* from the Xi in a very sensitive manner has been lacking. We provide here a new mouse model where *Mecp2* has been fused with the bioluminescent reporter NLuc, which is 100 times brighter than the frequently used firefly luciferase, and a fluorescent reporter, Tomato. This dual capability permits

(C) Violin plots of the number of LINEs and SINEs in a window of ±100 kb around the TSSs of non-reactivated genes (blue) and reactivated genes (green). ***p < 0.001, Mann-Whitney test.

(D) Average density plots of CTCF and Rad21 binding to, and H3K4me3 and H3K27me3 deposition at, the TSS ± 3 kb of the different gene subclasses on the Xa and Xi, and CHART-seq composite *Xist* enrichment at gene bodies (TSS-TES [transcription end site]) ± 3 kb. *Xist* was removed from the escapee list here in order not to bias the escapee analysis.

(E) Genome browser overview showing several genetic and epigenetic features from female NPCs, split into Xa- and Xi-specific signals. Female NPC Hi-C data from Bauer et al. (2021) are shown. For each TAD, the number of overlapping reactivated genes and escapees is identified, as plotted here along the X chromosome. The beige and red rectangles show the number of genes and reactivated genes per TAD, respectively. The $-$log10 of the p value of a binomial test between the ratio of reactivated genes/total genes per TAD and for the whole X chromosome is shown in blue for each TAD. TADs with a higher or lower ratio than average are plotted in inverse directions. A p value of 0.05 is indicated by a dotted line, and TADs with significantly higher or lower ratios are indicated by * or #, respectively. SINE, LINE, CpG, *Xist* (CHART-seq), CTCF, Rad21, H3K4me3, and H3K27me3 densities along the X chromosome are depicted in different colors. Data for CTCF, Rad21, H3K4me3, and H3K27me3 are split into Xa- and Xi-specific densities.

not only measurement of NLuc activity at a populational level, but also measurement of Tomato fluorescence at the single-cell level and *in vivo*. *Mecp2*^{NLucTom} mice are viable and have been created in a Cast/EiJ background that allows tracking of the level of reactivation in a chromosome-X and genome-wide manner, thanks to the presence of hundreds of thousands of informative SNPs with respect to the more commonly used C57BL/6 or 129/Sv strains.

By using Bl6 females carrying an oocyte-specific *Zp3-Cre* transgene and a *Xist*^{2lox} allele, we have generated a maternal knockout of *Xist*. Crossing these females with *Mecp2*^{NLucTom} cast males has allowed us to generate *Xist*$^{-/+}$:*Mecp2*$^{+/NLucTom}$ embryos. An alternative model where the females carry the *Zp3-Cre*, *Xist*^{2lox}, and *Mecp2*^{2lox} alleles has allowed us to generate *Xist*$^{-/+}$:*Mecp2*$^{-/NLucTom}$ embryos, that is, *Mecp2* knockouts based on expression. We have derived ESCs, MEFs, and NSCs from these F1 embryos. *Xist*$^{-/+}$:*Mecp2*$^{+/NLucTom}$ and *Xist*$^{-/+}$:*Mecp2*$^{-/NLucTom}$ NSCs show skewed XCI, as expected, by the presence of the *Xist* deletion on the maternal Bl6 X chromosome, while not showing any *in vitro* escape of *Mecp2* from the Xi. Why our *Xist*$^{-/+}$:*Mecp2*$^{-/NLucTom}$ neurons do not show RTT-related phenotypes is unclear. Most RTT-affected neuronal studies have been performed with *ex vivo* neuronal cultures (Baj et al., 2014; Rangasamy et al., 2016; Rietveld et al., 2015). However, *Mecp2* knockout neurons obtained by ESC differentiation showed smaller nuclear size than WT neurons after long-term culture *in vitro* (Yazdani et al., 2012). It is thus possible that our 10- or 11-day NSC differentiation is not sufficient to bring RTT phenotypes to the fore.

We have tested several compounds to assess whether the reporters can be reactivated. In contrast to what has been previously published (Przanowski et al., 2018), neither individual nor combined treatments with LDN193189, GSK650394, or RG2833 resulted in *Mecp2* reactivation in our *Xist*$^{-/+}$:*Mecp2*$^{-/NLucTom}$ NSCs. These differences might be due to Przanowski and colleagues using fibroblasts and adult brains instead of NSCs, or our NSCs might be more resilient to reactivation. Another possible reason for these drugs not to properly lead to XCR might be related to our cells being generated in a different and mixed genetic background. In addition, another inhibitor of ACVR1, K0228, also failed to reactivate a *Mecp2*-luciferase reporter in mouse tail fibroblasts (Lee et al., 2020). In conclusion, in our hands, combined treatment with GSK650394, LDN193189, and 5-Aza resulted in similar reactivation of *Mecp2* compared with 5-Aza only.

We have synergistically reactivated our NLuc-Tomato reporter with a combined treatment of 5-Aza and *Xist* knockdown. FACS analysis showed that a small population of treated cells shifted toward high Tomato fluorescence, while RNA-seq analysis indicated that a substantial population of cells in this fraction respond to the treatment and reactivate *Mecp2*, although, as expected, reactivation is not *Mecp2* specific. Eighty-five additional genes become significantly reactivated and several among these are more easily reactivated than *Mecp2*, and this will have to be taken into consideration when using general XCR methods with drugs as therapeutic treatments of RTT. Strikingly, we observed a significant overlap between our reactivated gene pool and genes reactivated at early and intermediate stages by means of iPSC reprogramming of female MEFs and female NPCs (Bauer et al., 2021; Janiszewski et al., 2019). It was intriguing to detect so many escapee genes listed in this last study as present in our reactivated gene list, suggesting these might be genes improperly silenced during their ESC differentiation process toward neuronal progenitors. In contrast, the NSCs in this study were isolated *de novo* from embryos and hence may have gone through a more robust XCI process *in vivo*. Altogether, we conclude that many X-linked genes show a predisposition to reactivate regardless of the technique, be it *Xist* knockdown combined with 5-Aza treatment or overexpression of the OCT4, SOX2, KLF4, or MYC transcription factors.

We examined which genetic or epigenetic mechanisms leading to XCR are at play here. Correlation analysis of reactivated genes with CpG presence and methylation loss after 5-Aza treatment indicates that although increased CpG presence is an indicator of reactivated genes, their reactivation surprisingly does not always seem to be associated with methylation loss. However, this can be reconciled with the fact that a small reduction in promoter methylation or loss of methylation at specific sites, not detectable by MeD-seq, might be sufficient for gene reexpression and may also explain why we detect only limited reactivation of *Mecp2* by NLuc activity analysis. We found that reactivated genes have decreased distances from escapees and that increased SINE and decreased LINE densities are potent indicators of reactivation. Correlating with our study, genes that are more easily silenced on the X chromosome or are ectopically silenced on an autosome carrying a *Xist* transgene tend to have more LINEs and fewer SINEs close to their TSSs (Loda et al., 2017). Moreover, in line with our results, X-linked genes that are reactivated early during iPSC reprogramming of female MEFs or NPCs harbor an increased number of SINEs closer to them than late or very late reactivating genes (Bauer et al., 2021; Janiszewski et al., 2019). There are thus strong indications that SINEs and LINES may play important roles in the capability of genes to be silenced or reactivated. SINE-mediated expansion of CTCF binding sites might explain why we detect increased binding of CTCF around reactivated genes on

the Xa and an increased number of SINEs closer to reactivated genes (Bourque et al., 2008; Schmidt et al., 2012). Nevertheless, reactivated genes show an enrichment of all subclasses of SINEs irrespective of their type, and are thus not limited to CTCF-enriched SINE B2 transposable elements (Schmidt et al., 2012). Also, we could not find any correlation between reactivation and different LINE types, organized either by evolutionary age or by size, indicating that genes prone to reactivate have fewer LINEs nearby, independent of the LINE size or age. Although we could not study SINE and LINE methylation directly, we found that genomic regions surrounding SINEs and LINEs that are close to reactivated genes do not show methylation differences from SINEs and LINEs that are in the vicinity of non-reactivated genes.

How SINEs might be involved in silencing and reactivation of X-linked genes remains an open question, but SINEs may be involved in setting up higher-order chromatin structure to overcome gene repression. In addition, the deposition of H3K4me3, a mark of promoter activity on the Xa, also tends to correlate with XCR, indicating that genes with strong activity signatures on the Xa are more easily reactivated, probably owing to their higher capacity to attract transcription factors. Finally, reactivated genes show more presence of *Xist* at their TSSs than non-reactivated genes. This might be explained by their high activity signature when on the Xa, which tends to attract *Xist* more easily (Simon et al., 2013).

Finally, because higher-order chromatin structure may play an important role in reactivation, we interrogated the proclivity of X-linked TADs to reactivate. Likely because we selected a reactivated population based on Tomato fluorescence, we find that the TAD containing *Mecp2* is more easily reactivated than other TADs. Three other TADs also show a tendency to more easily reactivate than other TADs. Their tendency to reactivate correlates again with a higher presence of SINEs, CTCF, and H3K4me3 when on the Xa, in line with our results showing SINEs to be strong indicators of reactivation potential and increased CTCF and H3K4me3 signals at the TSS of reactivated genes on the Xa.

In conclusion, genes that are reactivated by *Xist* knockdown and 5-Aza treatment overlap significantly with genes that are reactivated by other means, namely during reprogramming of MEFs and NPCs toward iPSCs, suggesting general intersecting mechanisms for XCR. We describe here a new mouse model system that is more sensitive than any bioluminescent or fluorescent system currently available in the community to study reactivation of *Mecp2*, *in vitro* and *in vivo*; however, RTT reversal phenotypes that occur on *Mepc2* reactivation will have to be studied with NSCs differentiated *in vitro* into neurons for longer, *ex vivo* neurons, or *in vivo*. These mouse lines could be used to study *Mecp2* reactivation by high-throughput screening of chemical compounds or by more targeted approaches, such as CRISPR-Cas9 fused to activators or repressors.

EXPERIMENTAL PROCEDURES

Mouse lines

All animal experiments were performed according to the legislation of the Erasmus MC Rotterdam Animal Experimental Commission. *Xist*^{2lox} mice were crossed with *Mecp2*^{2lox} mice to generate a colony of *Xist*$^{2lox/2lox}$:*Mecp2*$^{2lox/2lox}$ mice. *Xist*$^{2lox/2lox}$ and *Xist*$^{2lox/2lox}$:*Mecp2*$^{2lox/2lox}$ female mice were crossed with male *Zp3-Cre* mice to generate *Xist*$^{2lox/+}$ *Zp3-Cre* and *Xist*$^{2lox/+}$:*Mecp2*$^{2lox/+}$ *Zp3-Cre* females. These were then crossed with cast *Mecp2*$^{NLucTom/Y}$ males to generate *Xist*$^{-/+}$:*Mecp2*$^{+/NLucTom}$, *Xist*$^{-/+}$:*Mecp2*$^{-/NLucTom}$, and *Xist*$^{+/+}$:*Mecp2*$^{+/NLucTom}$ female hybrid embryos. *Mecp2*$^{NLucTom/NLucTom}$ female embryos were obtained from the *Mecp2*^{NLucTom} cast colony. WT hybrid females were obtained by crossing Bl6 females with cast males.

Cell culture

All ESC lines were grown in a regular ESC medium (DMEM, 10% fetal calf serum, 100 U mL^{-1} penicillin/streptomycin, 0.1 mM 2-mercapoethanol, 0.1 mM non-essential amino acids [NEAA], 5,000 U mL^{-1} leukemia inhibitory factor [LIF]) supplemented with 2i (1 μM PD0325901, Selleckchem; 3 μM CHIR99021, Axon Medchem) on irradiated male MEFs. An extended description of cell isolations and growth culture conditions is provided in the supplemental information.

RNA sequencing and MeD-seq

A detailed description is provided in the supplemental information.

SUPPORTING CITATIONS

The following reference appears in the supplemental information: Csankovszki et al., 1999.

Data and code availability

All raw and processed high-throughput sequencing data (RNA-seq, MeD-seq) generated in this study have been submitted to the NCBI Gene Expression Omnibus (GEO) under accession number https://www.ncbi.nlm.nih.gov/geo/query/acc.cgi?acc=GSE166147.

SUPPLEMENTAL INFORMATION

Supplemental information can be found online at https://doi.org/10.1016/j.stemcr.2022.01.008.

AUTHOR CONTRIBUTIONS

H.M.-B. contributed to the design of the study, performed experiments, analyzed the data, interpreted the results, and wrote the manuscript. B.T. analyzed the data, interpreted the results, and

edited the manuscript. C.G. and S.G. performed experiments, analyzed the data, and interpreted the results. R.G.B. and J.B. analyzed the data and interpreted the results. C.D. performed experiments. M.v.R. and W.v.I. analyzed the data. P.F. and A.B. contributed to the design of the study. J.G. contributed to the design of the study and to the interpretation of the results and edited the manuscript.

CONFLICT OF INTERESTS

The authors declare no competing interests or financial interests, except for R.B., J.B., W.v.I., and J.G., who report being shareholders in Methylomics B.V., a commercial company that applies MeD-seq to develop methylation markers for cancer staging. J.G. is a co-founder of Methylomics and a member of its scientific advisory board.

ACKNOWLEDGMENTS

We thank Esther Sleddens-Linkels and Tsung Wai Kan, from the Developmental Biology and Biochemistry Departments, Erasmus MC, for help with cell sorting. We thank Martí Quevedo and Raymond Poot from the Cellular Biology Department, Erasmus MC, for help with mouse NSC isolation and growth and comments on the manuscript. We also thank Robin Adrianse and Smitha Sripathi from the Fred Hutchinson Cancer Research Center for kindly offering plasmid information and 5-Aza advice. We thank Laura Mezzanotte and Yanto Ridwan from the Radiology and Nuclear Medicine Department, Erasmus MC, for helping with furimazine injections and imaging. Finally, we thank Maurice de Wit from the Department of Neurology, Erasmus MC, for help with taking pictures of neural cultures and neurite analysis. This research was funded by the Rett Syndrome Research Trust.

Received: March 19, 2021
Revised: January 10, 2022
Accepted: January 10, 2022
Published: February 10, 2022

REFERENCES

Adrianse, R.L., Smith, K., Gatbonton-Schwager, T., Sripathy, S.P., Lao, U., Foss, E.J., Boers, R.G., Boers, J.B., Gribnau, J., and Bedalov, A. (2018). Perturbed maintenance of transcriptional repression on the inactive X-chromosome in the mouse brain after Xist deletion. Epigenetics Chromatin *11*, 50.

Baj, G., Patrizio, A., Montalbano, A., Sciancalepore, M., and Tongiorgi, E. (2014). Developmental and maintenance defects in Rett syndrome neurons identified by a new mouse staging system in vitro. Front. Cell Neurosci. *8*, 18.

Bauer, M., Vidal, E., Zorita, E., Üresin, N., Pinter, S.F., Filion, G.J., and Payer, B. (2021). Chromosome compartments on the inactive X guide TAD formation independently of transcription during X-reactivation. Nat. Commun. *12*, 3499.

Bhatnagar, S., Zhu, X., Ou, J., Lin, L., Chamberlain, L., Zhu, L.J., Wajapeyee, N., and Green, M.R. (2014). Genetic and pharmacological reactivation of the mammalian inactive X chromosome. Proc. Natl. Acad. Sci. U S A *111*, 12591–12598.

Boers, R., Boers, J., de Hoon, B., Kockx, C., Ozgur, Z., Molijn, A., van IJcken, W., Laven, J., and Gribnau, J. (2018). Genome-wide DNA methylation profiling using the methylation-dependent restriction enzyme LpnPI. Genome Res. *28*, 88–99.

Bonev, B., Cohen, N.M., Szabo, Q., Fritsch, L., Papadopoulos, G.L., Lubling, Y., Xu, X., Lv, X., Hugnot, J.-P., Tanay, A., et al. (2017). Multiscale 3D genome rewiring during mouse neural development. Cell *171*, 557–572.e24.

Bourque, G., Leong, B., Vega, V.B., Chen, X., Lee, Y.L., Srinivasan, K.G., Chew, J.-L., Ruan, Y., Wei, C.-L., Ng, H.H., et al. (2008). Evolution of the mammalian transcription factor binding repertoire via transposable elements. Genome Res. *18*, 1752–1762.

Carrette, L.L.G., Wang, C.-Y., Wei, C., Press, W., Ma, W., Kelleher, R.J., and Lee, J.T. (2018). A mixed modality approach towards Xi reactivation for Rett syndrome and other X-linked disorders. Proc. Natl. Acad. Sci. U S A *115*, E668–E675.

Cattanach, B.M., and Williams, C.E. (1972). Evidence of non-random X chromosome activity in the mouse. Genet. Res. *19*, 229–240.

Chu, C., Zhang, Q.C., da Rocha, S.T., Flynn, R.A., Bharadwaj, M., Calabrese, J.M., Magnuson, T., Heard, E., and Chang, H.Y. (2015). Systematic discovery of Xist RNA binding proteins. Cell *161*, 404–416.

Csankovszki, G., Panning, B., Bates, B., Pehrson, J.R., and Jaenisch, R. (1999). Conditional deletion of Xist disrupts histone macroH2A localization but not maintenance of X inactivation. Nat. Genet. *22*, 323–324.

Fang, J., Chen, T., Chadwick, B., Li, E., and Zhang, Y. (2004). Ring1b-mediated H2A ubiquitination associates with inactive X chromosomes and is involved in initiation of X inactivation. J. Biol. Chem. *279*, 52812–52815.

Gendrel, A.-V., Tang, Y.A., Suzuki, M., Godwin, J., Nesterova, T.B., Greally, J.M., Heard, E., and Brockdorff, N. (2013). Epigenetic functions of smchd1 repress gene clusters on the inactive X chromosome and on autosomes. Mol. Cell Biol. *33*, 3150–3165.

Giacometti, E., Luikenhuis, S., Beard, C., and Jaenisch, R. (2007). Partial rescue of MeCP2 deficiency by postnatal activation of MeCP2. Proc. Natl. Acad. Sci. U S A *104*, 1931–1936.

Guy, J., Gan, J., Selfridge, J., Cobb, S., and Bird, A. (2007). Reversal of neurological defects in a mouse model of Rett syndrome. Science *315*, 1143–1147.

Janiszewski, A., Talon, I., Chappell, J., Collombet, S., Song, J., Geest, N.D., To, S.K., Bervoets, G., Marin-Bejar, O., Provenzano, C., et al. (2019). Dynamic reversal of random X-Chromosome inactivation during iPSC reprogramming. Genome Res. *29*, 1659–1672.

Lee, H.-M., Kuijer, M.B., Blanes, N.R., Clark, E.P., Aita, M., Arjona, L.G., Kokot, A., Sciaky, N., Simon, J.M., Bhatnagar, S., et al. (2020). A small-molecule screen reveals novel modulators of MeCP2 and X-chromosome inactivation maintenance. J. Neurodevelop. Disord. *12*, 29.

Lessing, D., Dial, T.O., Wei, C., Payer, B., Carrette, L.L.G., Kesner, B., Szanto, A., Jadhav, A., Maloney, D.J., Simeonov, A., et al. (2016). A high-throughput small molecule screen identifies

synergism between DNA methylation and Aurora kinase pathways for X reactivation. Proc. Natl. Acad. Sci. U S A *113*, 14366–14371.

Loda, A., Brandsma, J.H., Vassilev, I., Servant, N., Loos, F., Amirnasr, A., Splinter, E., Barillot, E., Poot, R.A., Heard, E., et al. (2017). Genetic and epigenetic features direct differential efficiency of Xist-mediated silencing at X-chromosomal and autosomal locations. Nat. Commun. *8*, 690.

Mak, W., Nesterova, T.B., de Napoles, M., Appanah, R., Yamanaka, S., Otte, A.P., and Brockdorff, N. (2004). Reactivation of the paternal X chromosome in early mouse embryos. Science *303*, 666–669.

McHugh, C.A., Chen, C.-K., Chow, A., Surka, C.F., Tran, C., McDonel, P., Pandya-Jones, A., Blanco, M., Burghard, C., Moradian, A., et al. (2015). The Xist lncRNA interacts directly with SHARP to silence transcription through HDAC3. Nature *521*, 232–236.

Minajigi, A., Froberg, J.E., Wei, C., Sunwoo, H., Kesner, B., Colognori, D., Lessing, D., Payer, B., Boukhali, M., Haas, W., et al. (2015). A comprehensive Xist interactome reveals cohesin repulsion and an RNA-directed chromosome conformation. Science *349*, 282.

Moindrot, B., Cerase, A., Coker, H., Masui, O., Grijzenhout, A., Pintacuda, G., Schermelleh, L., Nesterova, T.B., and Brockdorff, N. (2015). A pooled shRNA screen identifies Rbm15, spen, and Wtap as factors required for Xist RNA-mediated silencing. Cell Rep. *12*, 562–572.

Monfort, A., Minin, G.D., Postlmayr, A., Freimann, R., Arieti, F., Thore, S., and Wutz, A. (2015). Identification of spen as a crucial factor for Xist function through forward genetic screening in haploid embryonic stem cells. Cell Rep *12*, 554–561.

de Napoles, M., Mermoud, J.E., Wakao, R., Tang, Y.A., Endoh, M., Appanah, R., Nesterova, T.B., Silva, J., Otte, A.P., Vidal, M., et al. (2004). Polycomb group proteins Ring1A/B link ubiquitylation of histone H2A to heritable gene silencing and X inactivation. Dev. Cell *7*, 663–676.

Monkhorst, K., Jonkers, I., Rentmeester, E., Grosveld, F., and Gribnau, J. (2008). X inactivation counting and choice is a stochastic process: evidence for involvement of an X-linked activator. Cell *132*, 410–421.

Okamoto, I., Otte, A.P., Allis, C.D., Reinberg, D., and Heard, E. (2004). Epigenetic dynamics of imprinted X inactivation during early mouse development. Science (New York, NY) *303*, 644–649.

Plath, K., Talbot, D., Hamer, K.M., Otte, A.P., Yang, T.P., Jaenisch, R., and Panning, B. (2004). Developmentally regulated alterations in Polycomb repressive complex 1 proteins on the inactive X chromosome. J. Cell Biol. *167*, 1025–1035.

Przanowski, P., Wasko, U., Zheng, Z., Yu, J., Sherman, R., Zhu, L.J., McConnell, M.J., Tushir-Singh, J., Green, M.R., and Bhatnagar, S. (2018). Pharmacological reactivation of inactive X-linked Mecp2 in cerebral cortical neurons of living mice. Proc. Natl. Acad. Sci. U S A *115*, 7991–7996.

Rangasamy, S., Olfers, S., Gerald, B., Hilbert, A., Svejda, S., and Narayanan, V. (2016). Reduced neuronal size and mTOR pathway activity in the Mecp2 A140V Rett syndrome mouse model. F1000Res. *5*, 2269.

Rietveld, L., Stuss, D.P., McPhee, D., and Delaney, K.R. (2015). Genotype-specific effects of Mecp2 loss-of-function on morphology of Layer V pyramidal neurons in heterozygous female Rett syndrome model mice. Front. Cell Neurosci. *9*, 145.

Schmidt, D., Schwalie, P.C., Wilson, M.D., Ballester, B., Gonçalves, Â., Kutter, C., Brown, G.D., Marshall, A., Flicek, P., and Odom, D.T. (2012). Waves of retrotransposon expansion remodel genome organization and CTCF binding in multiple mammalian lineages. Cell *148*, 335–348.

Simon, M.D., Pinter, S.F., Fang, R., Sarma, K., Rutenberg-Schoenberg, M., Bowman, S.K., Kesner, B.A., Maier, V.K., Kingston, R.E., and Lee, J.T. (2013). High-resolution Xist binding maps reveal two-step spreading during X-chromosome inactivation. Nature *504*, 465–469.

Sookdeo, A., Hepp, C.M., McClure, M.A., and Boissinot, S. (2013). Revisiting the evolution of mouse LINE-1 in the genomic era. Mob DNA *4*, 3.

Sripathy, S., Leko, V., Adrianse, R.L., Loe, T., Foss, E.J., Dalrymple, E., Lao, U., Gatbonton-Schwager, T., Carter, K.T., Payer, B., et al. (2017). Screen for reactivation of MeCP2 on the inactive X chromosome identifies the BMP/TGF-β superfamily as a regulator of XIST expression. Proc. Natl. Acad. Sci. U S A *114*, 1619–1624.

Wang, C.-Y., Jégu, T., Chu, H.-P., Oh, H.J., and Lee, J.T. (2018). SMCHD1 Merges chromosome compartments and assists formation of super-structures on the inactive X. Cell *174*, 406–421.e25.

Weaving, L.S., Ellaway, C.J., Gécz, J., and Christodoulou, J. (2005). Rett syndrome: clinical review and genetic update. J. Med. Genet. *42*, 1.

Yazdani, M., Deogracias, R., Guy, J., Poot, R.A., Bird, A., and Barde, Y. (2012). Disease modeling using embryonic stem cells: MeCP2 regulates nuclear size and RNA synthesis in neurons. Stem Cells *30*, 2128–2139.

Stem Cell Reports, Volume *17*

Supplemental Information

Genetic and epigenetic determinants of reactivation of Mecp2 and the inactive X chromosome in neural stem cells

H. Mira-Bontenbal, B. Tan, C. Gontan, S. Goossens, R.G. Boers, J.B. Boers, C. Dupont, M.E. van Royen, W.F.J. IJcken, P. French, A. Bedalov, and J. Gribnau

Supplemental Information

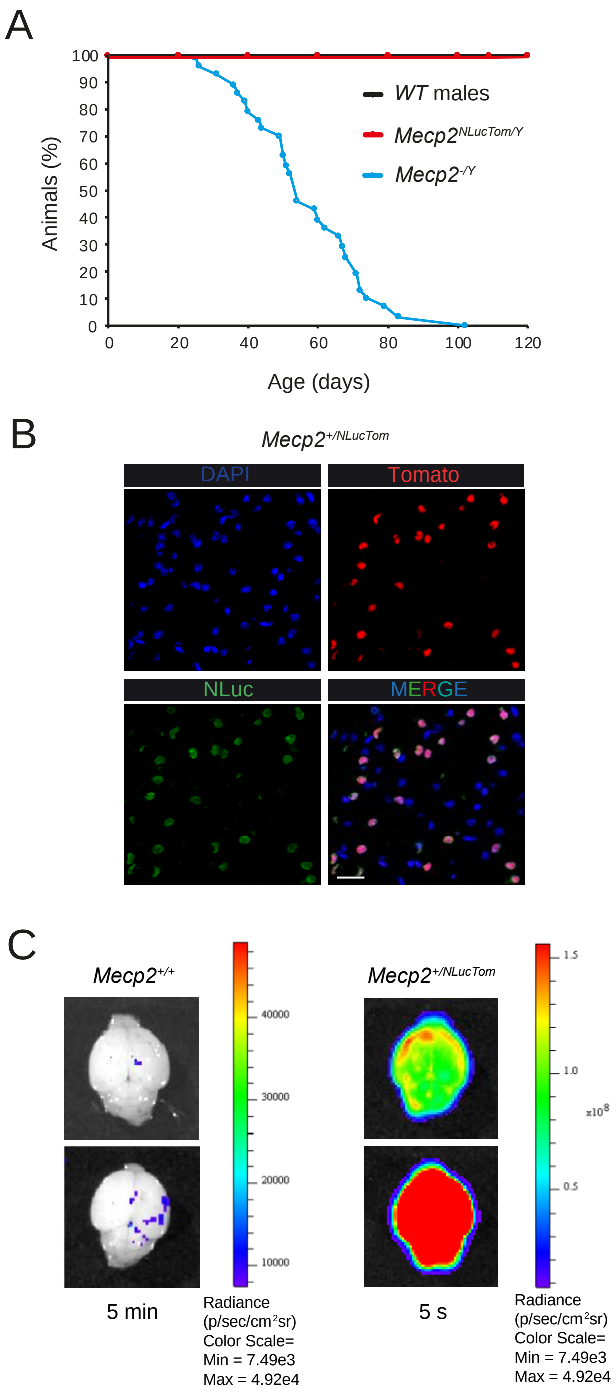

Figure S1. Cast *Mecp2-NLucTom* mice are viable and express the fusion protein in the brain (related to Figure 2).

(A) Cumulative survival plot showing the % of cast *Mecp2*$^{NLucTom/Y}$ males (red) compared to WT cast males (black) and *Mecp2*$^{\Delta/Y}$ males (blue)(Guy et al., 2001) surviving at a given time in days. (B) IF of NLuc (green) and endogenous Tomato fluorescence (red) in brain sections of heterozygote *Mecp2*$^{+/NLucTom}$ females showing random XCI. DAPI, blue. We quantified 86 cells

out of 193 cells (45%) from a single brain showing NLuc and Tomato signals. White scale bar: 25 µm.

(C) Representative bioluminescence images of two P6 WT female brains and two P6 *Mecp2*$^{+/NLucTom}$ female brains after 5 min or 5 s, respectively, of furimazine injection.

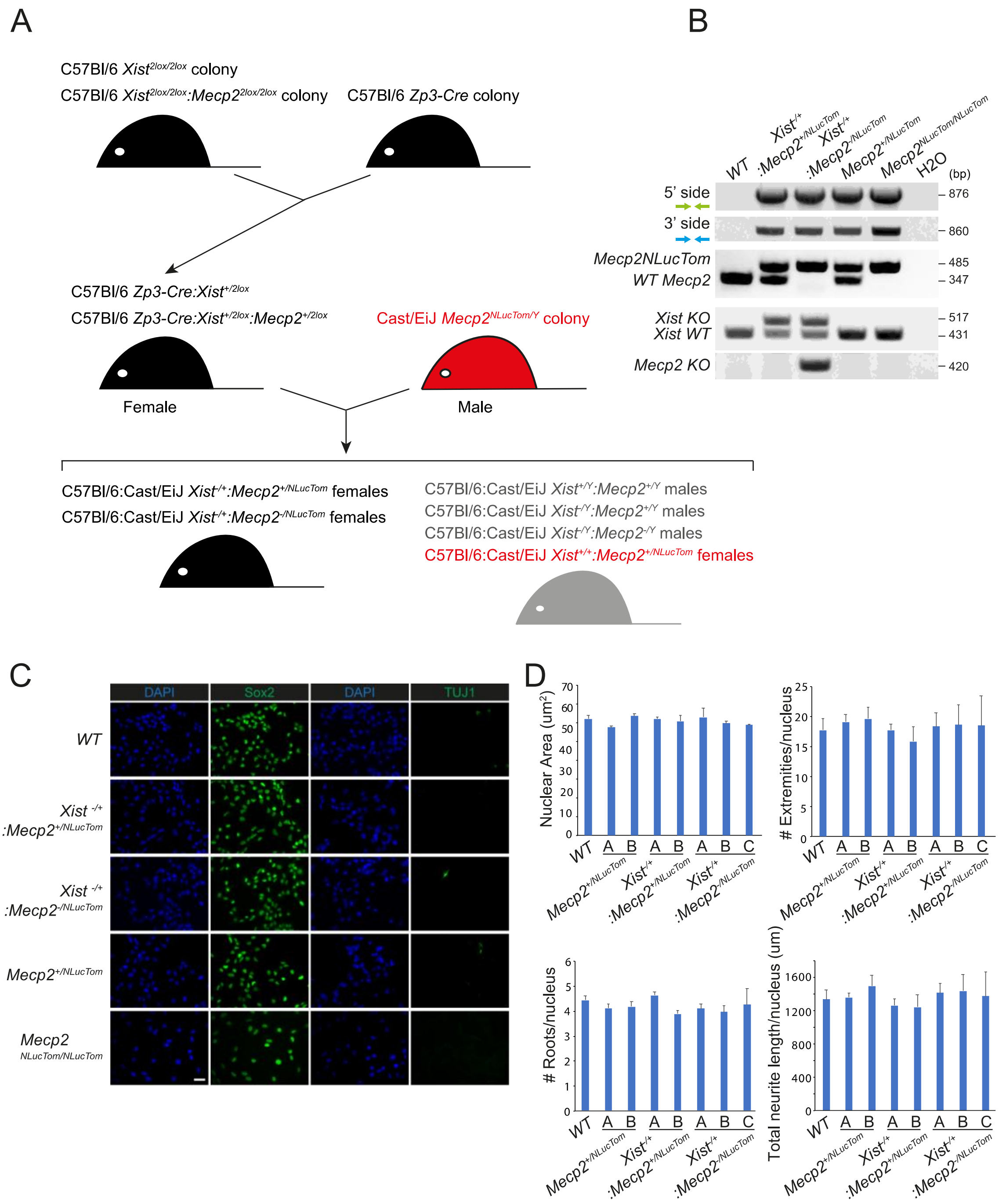

Figure S2. Generation of WT, *Xist*$^{-/+}$:*Mecp2*$^{+/NLucTom}$, *Xist*$^{-/+}$:*Mecp2*$^{-/NLucTom}$, *Mecp2*$^{+/NLucTom}$ and *Mecp2*$^{NLucTom/NLucTom}$ NSCs (related to Figure 2).

(A) Breeding strategy to generate *Xist*$^{-/+}$:*Mecp2*$^{+/NLucTom}$, *Xist*$^{-/+}$:*Mecp2*$^{-/NLucTom}$ embryos and associated lines. We keep four independent colonies in two backgrounds: C57BL/6 *Xist*$^{2lox/2lox}$, C57BL/6 *Xist*$^{2lox/2lox}$:*Mecp2*$^{2lox/2lox}$, C57BL/6 *Zp3-Cre* and Cast/EiJ *Mecp2*^{NLucTom} mice. *Xist*$^{2lox/2lox}$ and *Xist*$^{2lox/2lox}$:*Mecp2*$^{2lox/2lox}$ females or males are crossed with *Zp3-Cre* mice to generate heterozygous *Zp3-Cre:Xist*$^{+/2lox}$ and *Zp3-Cre:Xist*$^{+/2lox}$:*Mecp2*$^{+/2lox}$ females in a C57BL/6

background. These females are then crossed with *Mecp2*$^{NLucTom/Y}$ males, to generate hybrid *Xist*$^{-/+}$*:Mecp2*$^{+/NLucTom}$ or *Xist*$^{-/+}$*:Mecp2*$^{-/NLucTom}$ female embryos. Other possible genotypes of this last crossing are depicted in gray. Notice all genotypes from this second crossing can have Zp3-Cre (50% probability), not depicted in figure.

(B) Genotyping PCR of F1 NSCs with different genotypes. Primers for the 5' side and 3' side of the specific integration are the same primers as in Figure 1C. PCRs for the *Xist* WT and knockout alleles and *Mecp2* WT, tagged with NLucTom or knockout alleles are also shown. Note that *Xist*$^{-/+}$*:Mecp2*$^{-/NLucTom}$ cells do not show any WT Mecp2 allele since it is deleted on the maternal X chromosome.

(C) IF of Sox2 or TUJ1 (left and right green respectively) in WT, *Xist*$^{-/+}$*:Mecp2*$^{+/NLucTom}$, *Xist*$^{-/+}$*:Mecp2*$^{-/NLucTom}$, *Mecp2*$^{+/NLucTom}$ and *Mecp2*$^{NLucTom/NLucTom}$ NSCs. DAPI, blue. White scale bar: 25 µm. n=1.

(D) Nuclear area, number of cellular extremities per nucleus and number of roots per nucleus (average ± s.d., n=3 biological replicates, 191-521 neurons per replicate) of 2-3 independent WT, *Xist*$^{-/+}$*:Mecp2*$^{+/NLucTom}$ and *Xist*$^{-/+}$*:Mecp2*$^{-/NLucTom}$ NSC clones differentiated to neurons.

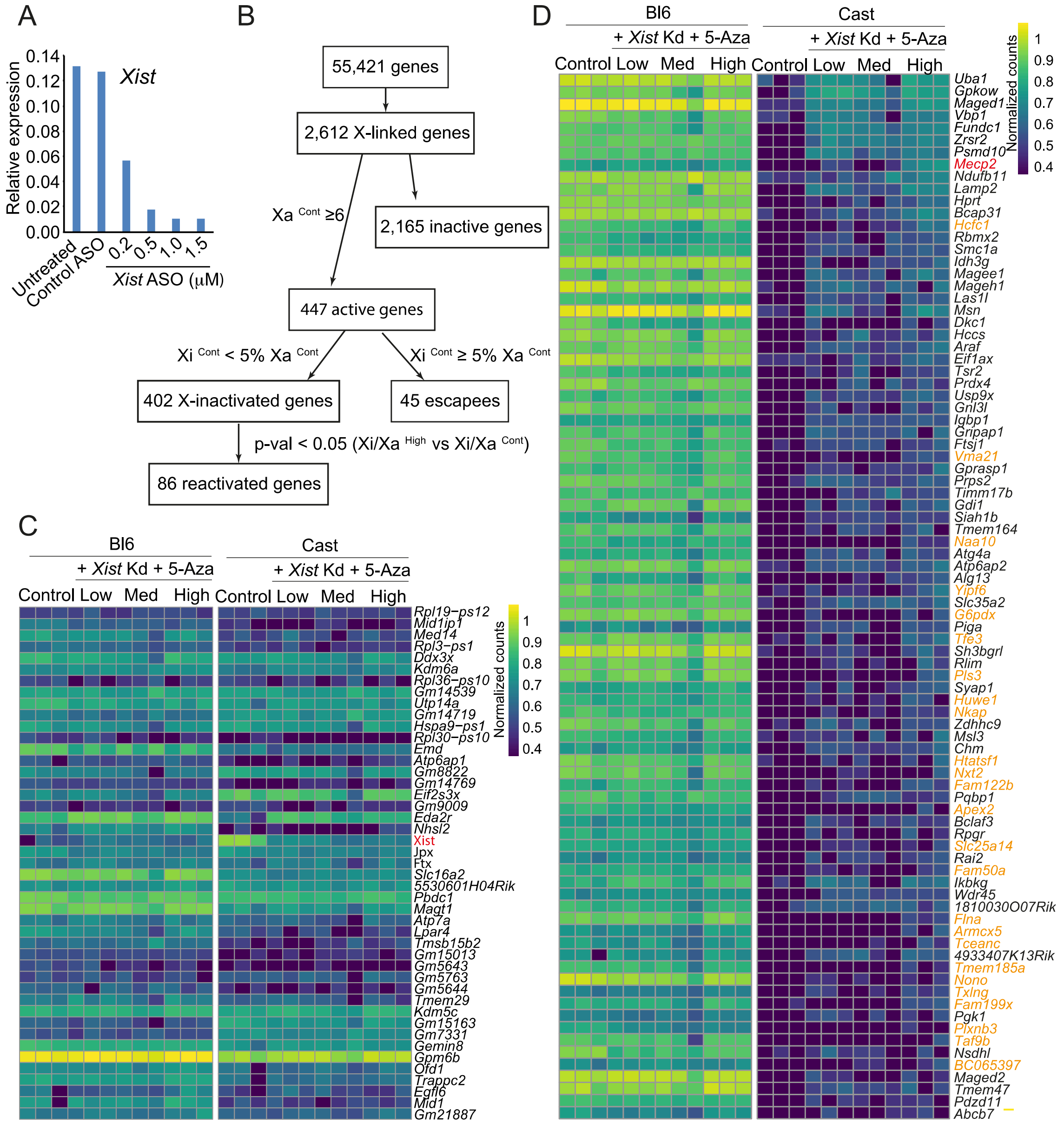

Figure S3. Many X-linked genes are reactivated in mNSCs after *Xist* knockdown and 5-Aza treatment (related to Figures 3 and 4).

(A) Relative *Xist* expression by RT-qPCR analysis in *Xist*$^{-/+}$*:Mecp2*$^{+/NLucTom}$ NSCs after knockdown of *Xist* with *Xist* ASOs or control ASOs. Different concentrations of *Xist* ASOs were tested, n=1.

(B) Flowchart indicating the general steps performed to obtain the different gene subclasses from the RNA-seq analysis.

(C) Expression heatmap of the different escapees across the different samples and alleles. *Xist* is indicated in red.

(D) Expression heatmap of the reactivated genes across the different samples and alleles, ordered by P-value. *Mecp2* is indicated in red. Genes that are also reactivated in the Tomato-Low

and/or Tomato-Medium populations are indicated in black, while genes only significantly reactivated in the Tomato-High population as *Mecp2* are indicated in orange.

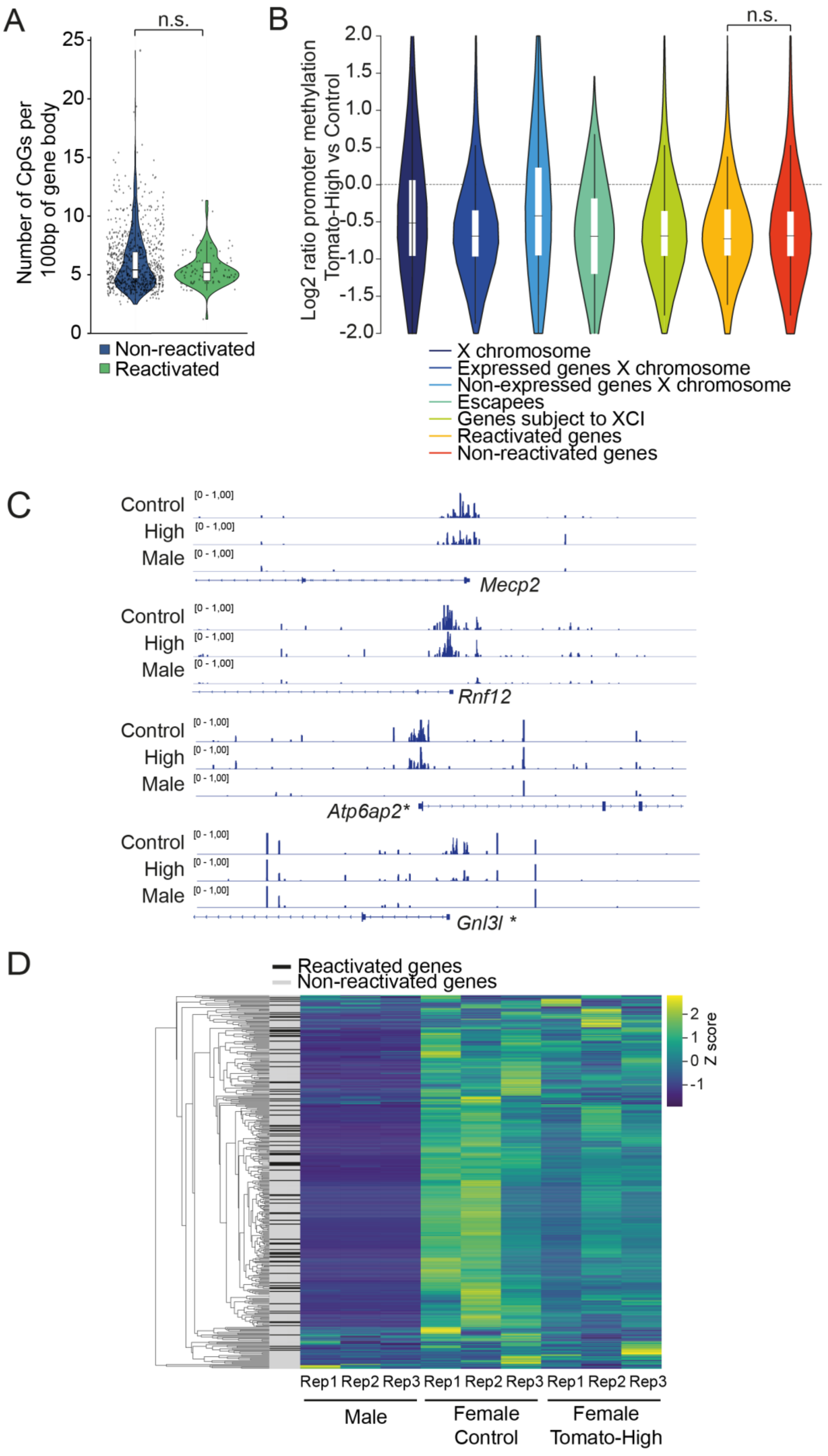

A
n.s.
Number of CpGs per 100bp of gene body
0
5
10
15
20
25
Non-reactivated
Reactivated
B
n.s.
Log2 ratio promoter methylation Tomato-High vs Control
2.0
1.5
1.0
0.5
0.0
-0.5
-1.0
-1.5
-2.0
X chromosome
Expressed genes X chromosome
Non-expressed genes X chromosome
Escapees
Genes subject to XCI
Reactivated genes
Non-reactivated genes
C
Control
High
Male
[0 - 1,00]
Mecp2
Rnf12
Atp6ap2*
Gnl3l *
D
Reactivated genes
Non-reactivated genes
Z score
2
1
0
-1
Rep1 Rep2 Rep3 Rep1 Rep2 Rep3 Rep1 Rep2 Rep3
Male
Female Control
Female Tomato-High

Figure S4. Reactivation does not correlate with a general loss of methylation at promoters (related to Figure 5).

(A) Violin plot depicting the number of CpGs per 100bp of gene body of reactivated and non-reactivated genes; n.s., not significant (Mann-Whitney test, p-value < 0.05).

(B) Violin plots depicting the log2 ratio between the methylation status of the promoter (± 1 Kbp around TSS) of the different gene subclasses in the Tomato-High and control populations. Dotted line indicates an identical methylation status for both conditions (ratio = 1); n.s., not significant (Mann-Whitney test, p-value < 0.05).

(C) Genome browser overview with the average normalized MeD-seq tracks at the promoter areas of four reactivated genes *Mecp2*, *Rnf12, Atp6ap2* and *Gnl3l* in female control, female Tomato-High and male NSCs. Genes with significant loss of DNA methylation at their promoters (±1 Kbp of the TSS) are indicated by an asterisk.

(D) Heatmap of the DNA methylation status around the TSSs of reactivated genes and non-reactivated genes of the three biological replicates of female control, female Tomato-High and male NSCs. Z-scores of MeD-seq read counts ±1 Kbp of the TSS are shown. Next to the clustering dendrogram, genes are annotated as reactivated and not-reactivated in black and gray, respectively.

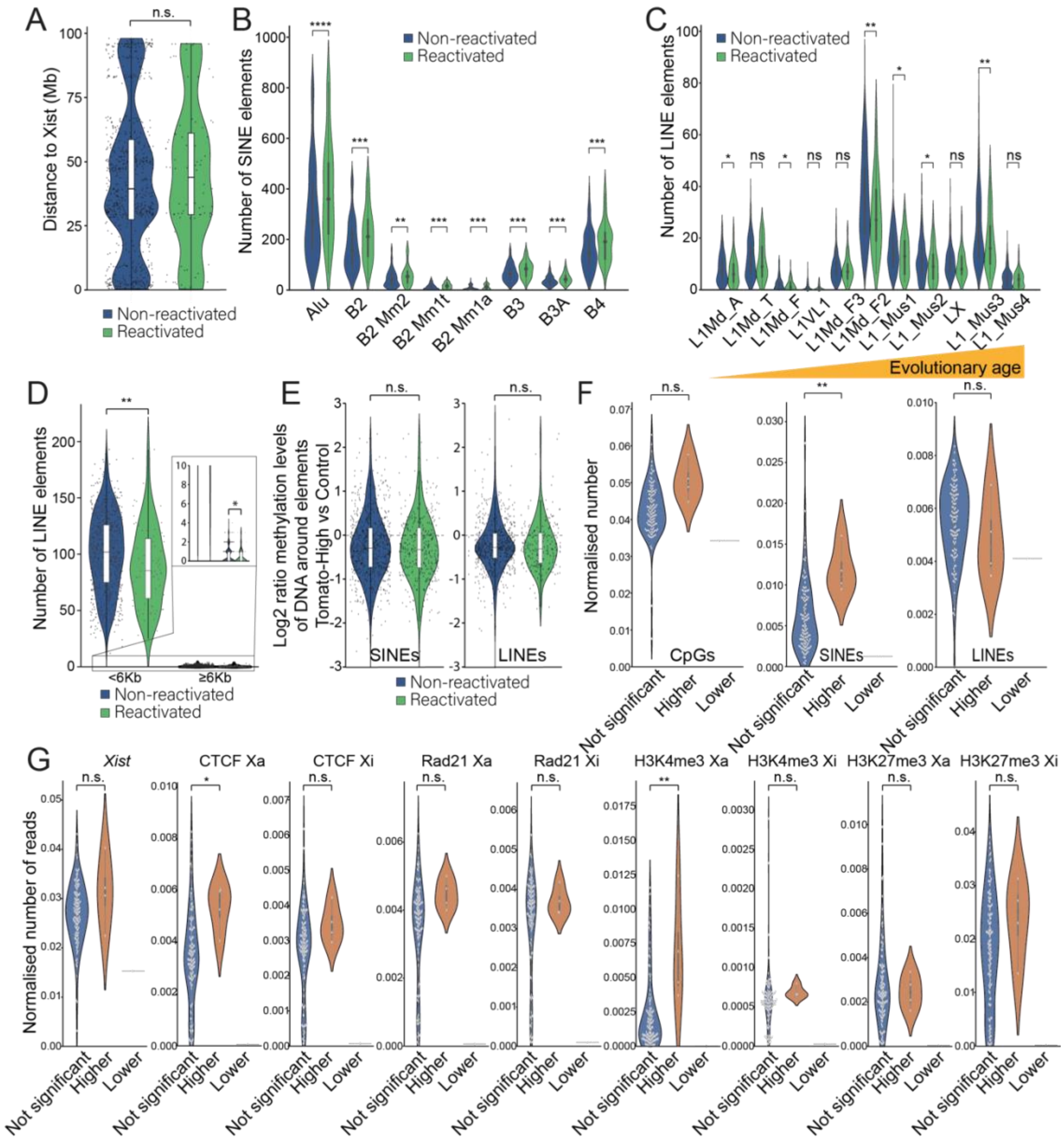

Figure S5. Reactivation correlates with several genetic and epigenetic marks (related to Figure 5).

(A) Violin plots depicting the distance to *Xist* in Mb of non-reactivated genes (blue) and reactivated genes (green). n.s., not significant (Mann-Whitney test, p-value < 0.05).

(B) Violin plots depicting the number of different SINE subclasses in ± 500 Kbp bins around the TSS of non-reactivated genes (blue) and reactivated genes (green). ** p-val<5E-3, *** p-val<5E-4, **** p-val<5E-5, Mann-Whitney test.

(C) Violin plots depicting the number of different LINE subclasses organized by evolutionary age (left, young; right, old) in ± 500 Kbp bins around the TSS of non-reactivated genes (blue) and reactivated genes (green). * p-val<0.05, ** p-val<0.01, Mann-Whitney test.

(D) Violin plots depicting the number of LINEs organized by size (<6 Kbp or ≥6 Kbp) in ± 100 Kbp bins around the TSS of non-reactivated genes (blue) and reactivated genes (green). *, p-val<0.05, ** p-val<0.01, Mann-Whitney test.

(E) Violing plots depicting the levels of DNA methylation around LINEs and SINEs (±200 bp) situated ± 100 Kbp around the TSSs of non-reactivated genes (blue) and reactivated genes (green) in the Tomato-High population vs Control.

(F) Violin plots of the normalized number of CpGs, SINEs and LINEs in TADs that are significantly enriched (higher) or depleted (lower) for reactivated genes or not significant. ** p-val<0.01, Mann-Whitney test; n.s. not significant.

(G) Violin plots of the normalized number of CHART-seq reads of *Xist* presence and ChIP-seq reads of CTCF, Rad21, H3K4me3 and H3K27me3 on the Xa and Xi in TADs that are not significantly enriched for reactivated genes and TADs that are significantly enriched (higher) or depleted (lower) for reactivated genes. * p-val<0.05, ** p-val<0.01, Mann-Whitney test; n.s., not significant.

		Forward	Reverse
PCR	3' side	ACTGTGCTCCCCATCAAGAA	AGTGTGCCATAGTGCAGGAT
PCR	5' side	TCCCACAACGAGGACTACAC	TCCATGGCACCTCTTTAGCA
PCR	Endogenous/ WT *Mecp2*	ATGCTCCAACTACTCCCACC	CCAAGGAGCCAGCTAAGACT
PCR	*Rnf12* control	GCCTTCGAACATCTCTGAGC	GAGCCGGACTAATCCAAACA
PCR	*Xist 2lox/null/*WT *alleles*	CACTGGCAAGGTGAATAGCA and ACCCTTGCCTTTTCCATTTT	CTTCTGGTCTTTGAGGGCAC
PCR	*Mecp2-NLuc-Tom*	CGAGAGAGTTAGCGGATCCA	TTACTCGGAACAGCAGGGAG
PCR	Mecp2 null allele	TGG TAA AGA CCC ATG TGA CCC AAG	TCCACCTAGCCTGCCTGTACT TTG
RT-qPCR	*b-actin*	AACCCTAAGGCCAACCGTGAA AAG	CATGGCTGGGGTGTTGAAGGT CTC
RT-qPCR	*Xist*	GGATCCTGCTTGAACTACTGC	CAGGCAATCCTTCTTCTTGAG
RT-qPCR	*NLuc*	GGCTACAACCTGGACCAAGT	TGGATCGGAGTTACGGACAC

Table S1. Primers used in this study (related to Figures 1 and S2).

Table S2. X-linked differential expression analysis between the Tomato-High and Control populations.

This table shows the differentially expressed X-linked genes between the Tomato-High and Control populations using DESeq2. Genes are annotated according to their gene group and X-inactivated genes that are reactivated in the Low and Medium samples are indicated.

Table S3. DNA methylation analysis of X-linked promoters using MeD-seq.

This table shows the normalized MeD-seq reads of male, Tomato-High and Control populations at the X-linked gene promoters (± 1kb of the TSS). Fold change between Tomato-High and Control are reported, as well as the p-value based on a Mann-Whitney test. Genes are annotated according to their gene class based on the RNA-seq data.

Supplemental Experimental Procedures

Mouse lines

All animal experiments were performed according to the legislation of the Erasmus MC Rotterdam Animal Experimental Commission.

Mecp2^{NLucTom} mice were obtained from *Mecp2*^{NLucTom} ESCs generated in house (see below) and kept in a cast background colony. *Xist*^{2lox} mice (Csankovszki et al., 1999) were kept in a B6 background. *Mecp2*^{2lox} and *Zp3-Cre* mice were obtained from the Jackson Laboratory (B6.129P2-MeCP2tm1Bird/J - #077177, C57BL/6-Tg(Zp3-Cre)93Kw/J - #003651 respectively) and kept in a B6 background. A detailed description of the crosses is provided in supplementary experimental procedures.

Cell culture

ESCs were generated from E3.5 blastocysts. Briefly, E3.5 blastocysts were flushed from uteri in M2 medium. Their zona pellucida was removed with acidic Tyrode's solution (Sigma) at RT for several seconds. Embryos were subsequently washed in M2 medium and transferred to 4-well plates, one blastocyst per well, containing irradiated MEFs and regular ESC medium supplemented twice as much as the normal amount of LIF and PD98059 (final concentration 50 µM; Cell Signaling Technologies). ICM outgrowths were then picked 5-7 days plating the blastocyst and expanded. Once ESCs reached the 12 well stage, they were genotyped. The selected genotypes were grown as single cells/colonies by plating them in serial dilutions in 10 cm dishes. One day after plating, cells were weaned off the increased concentration of LIF and of PD98059. Colonies were picked, selected for morphology and correct karyotype.

To target *Mecp2* in male cast ESC cells, 0.6x10$^{\wedge 6}$ ESCs were transfected with 2 µg CRISPR/Cas9 targeting the *Mecp2* STOP codon and 2 µg of the donor vector carrying 5' and 3' 500bp-long cast-specific homology arms with a NLuc and Tomato reporters in frame with *Mecp2*'s coding region. A P2A signal between NLuc and Tomato leads to Tomato being translated as an independent protein. ESCs were transfected with 4 µL lipofectamine 2000 following the manufacturer's instructions (ThermoFisher). 2 days after transfection, Tomato-positive cells were FACS-sorted, put back into culture for a few days and subsequently injected in B6 blastocysts.

MEFs were isolated from E12.5 embryos. E12.5 embryos were removed from yolk sacs and their heads, liver, hearts and digestive tract were removed. The remaining carcass was chopped into fine pieces and added to 5 mL of Trypsin-EDTA (Life Technologies), for 10 min 37°C in water batch. Falcons were shaken smoothly every 2 min. Remaining clusters and cells were pipetted up and down several times. Medium was added to quench the trypsin and the sample was centrifuged 5 min 1000 rpm. Cell pellet was resuspended in regular ESC medium without LIF and 2i and grown in 0.2% gelatin-coated 15cm dishes. After 3x 15 cm dishes were obtained, cell lines were genotyped and frozen.

NSC lines were isolated from E15.5 embryos. E15.5 brains were extracted, hemispheres were cut and meninges dissected when possible. Cortexes were chopped into pieces and introduced in 15 mL falcon tubes containing 2.5 mL dissecting medium (PBS +3% glucose) plus 300 µL Trypsin-EDTA (10x; Life Technologies, 15400-054). The falcon was then incubated for 10 min at 37°C while shaking it every 2 min manually. Trypsin was then inactivated with 500 µL horse serum (Life Technologies, 16050-130). 50 µL DNaseI (1mg mL^{-1}) was added and incubated for 8-10 min at 37°C. Pipette up and down around 10 times with 1 mL pipette. Centrifuge 1000 rpm 5 min and resuspend in filtered NSC culture medium (Conti 2005): 192 mL EuroMed-N (EuroClone), 2 mL N2 supplement (Invitrogen), 1 mL human insulin (final concentration 20 µg mL^{-1}, Roche), 1ml BSA (final concentration 50 µg mL^{-1}, Gibco), 2 mL L-Glut (100x; Gibco), 10 µL murine EGF (final concentration 10 ng mL^{-1}, Peprotech), 10 µL human bFGF (final concentration 10 ng mL^{-1}, Peprotech), 100 U mL^{-1} penicillin/streptomycin. Cells were grown in suspension in 10cm dishes for a week. Resulting neurospheres were disaggregated and cells were grown as a monolayer. The different lines were established and grown in 6-well plates. Wells were precoated with 0.2% gelatin for 5 min RT, then removed and 1.5 mL Laminin (final concentration 5 µg mL^{-1} in PBS; Sigma) was added to wells for at least 5 h at 37°C, better o/n at 37°C, or kept in the fridge with parafilm for up to a month. Prior to use, wells were washed quickly 3x with PBS and cells added. Cells were passaged with Accutase (Sigma) every 3-5 days up to passage 10-12, generating frozen vials along the way.

NSC differentiation into neurons, astrocytes and oligodendrocytes was performed following the protocol by Spiliotopoulos and colleagues (Spiliotopoulos et al., 2009). NSCs were counted and seeded in 5 mL D1 medium (EuroMed-N, 0.5% N2 (Invitrogen), 1% B27 (Invitrogen),

10 ng mL^{-1} human bFGF and 100 U mL^{-1} penicillin/streptomycin) at 1.35x10^{5} cells cm^{-2} per well of a 6-well dish previously coated with 0.1% gelatin and 5 µg mL^{-1} laminin in PBS for at least 5 hours (day 0). 3 days after plating, cells were collected with accutase, counted and reseeded in 3 mL A medium (1:3 mix DMEM/F12 (Gibco) and Neurobasal (Gibco), 0.5% N2, 1% B27, 10 ng mL^{-1} bFGF, 20 ng mL^{-1} BDNF (Prospec) and 100 U mL^{-1} penicillin/streptomycin) at 5 x10^{4} cells cm^{-2} per well of a 6-well dish with coverslips precoated with gelatin and laminin (day 3). Medium was changed at day 5 and at day 6, medium was changed by B medium (1:3 mix DMEM/F12 and Neurobasal, 0.5% N2, 1% B27, 6.7 ng mL^{-1} bFGF, 30 ng mL^{-1} BDNF and 100 U mL^{-1} penicillin/streptomycin). At day 9, medium was changed to B1 medium (same as B medium although at 5 ng mL^{-1} bFGF). Coverslips were then fixed at day 10-11 after the start of differentiation following the IF protocol below.

Immunofluorescence

Mice were perfused with 4% PFA for 3 min. Brains were removed and fixed again in 4% PFA for 1 h at RT. Brains were left to sink o/n in 10% sucrose in PBS at 4°C and frozen in OCT the next day by bathing a freezing cup isopentane in dry ice and stored afterwards at -80°C. 7 µm coupes were generated with a cryostat microtome and put on adhesive slides. Slides were left to dry 30 min at RT and frozen at -80°C. Slides were thawed 30 min at RT and processed with the same IF protocol as cell cultures, see below.

IF on cell cultures were performed as follows. Cells were grown on coverslips and subsequently blocked in 5% goat serum (Sigma, G9023; or donkey serum when necessary, Sigma, D9963), 1% Triton X100 (performed) in PBS for 1 h at RT. The primary antibody was applied in PBS 5% goat (or donkey) serum, 0.1% Triton X1000 0/n at 4°C in the dark. The next day, slides were washed 3x with 5% goat (or donkey) serum, 0.1% Triton X100 in PBS for 5 min at RT. The second antibody was then applied in 5% goat (or donkey) serum, 0.1% Triton X100 in PBS for 1 h at RT and washed 3x with 5% goat (or donkey) serum, 0.1% Triton X100 in PBS for 5 min at RT (last wash containing 1:5000 DAPI). Slides were then mounted with Prolong™ Gold Antifade Mountant (Thermo Scientific). Images were acquired with a fluorescent Axio Imager M2 microscope (Zeiss) and analysed with Fiji and Photoshop software (Adobe). The following primary antibodies were used: mouse anti-TUJ1 (Biolegend, 801202, 1:250), rabbit anti-GFAP (Dako,

Z0334, 1:250), rabbit anti-OLIG2 (IBL, 18953, 1:250), goat anti-SOX2 (Santa Cruz, sc-17320, 1:500), rabbit anti-NLuc (kind gift of Promega, 1:100). The following Alexa Fluor secondary antibodies were used: goat anti-rabbit 488 (Invitrogen, A-11008, 1:400), goat anti-mouse 488 (Invitrogen, A-11001, 1:400) and goat anti-rabbit 633 (Invitrogen, A-21070, 1:400), donkey anti-goat 488 (Invitrogen, A-11055, 1:400). IFs of neuronal cultures were not performed with serum, BSA was used instead.

Xist knockdown and drug analysis

Xist knockdown was performed following manufacturer's instructions for the Mouse Neural Stem Cell Nucleofector Kit (Lonza). 3-5x10^6 NSCs were collected and resuspended in 70 µL Nucleofector solution, 15 µL supplement and 15 µL *Xist* ASO 10 µM (*XIST*-ANAND_1, cat. 339511 LG00116620-DDA: TCTTGGTTACTAACAG (Carrette et al., 2018); Qiagen). Cells were nucleofected with a Lonza Nucleofector™, program A-033. Cells were then put back into culture and treated with 5-Aza or its vehicle for 3 days.

Decitabine (Selleck Chem) was resuspended in aliquots of 10 µL 10 µM in DMSO and kept in an Argon atmosphere at -80°C. During the 7-day drug test, LDN193189, GSK650394, RG2833 and decitabine were used at 0.5 µM, 2.5 µM, 5 µM and 0.5 µM respectively (Carrette et al., 2018; Janiszewski et al., 2019). The RNA-seq analysis was performed on NSCs treated with 10 µM 5-Aza and 1.5 µM *Xist* ASO, or DMSO and 1 µM scrambled ASOs for 3 days.

Western Blot

Cells were harvested in ice-cold PBS with complete protease inhibitors (Roche). Cell pellets were incubated with 400 µL Buffer A (100 mM HEPES, 1.5 mM MgCl2, 10 mM KCl, 0.5 mM DTT and protease inhibitors) for 10 min on ice, vortexed 30 sec and centrifuges 2000 rpm, 5 min, 4ºC. Nuclei were then lysed by adding 2x the pellet volumes of Buffer C (20 mM HEPES, 25% glycerol, 420 mM NaCl, 1.5 mM MgCl2, 0.2 mM EDTA, 0.5 mM DTT and protease inhibitors) for 20 min on ice, centrifuged max speed, 2min, 4ºC. Protein concentrations were determined with NanoDrop. WB was performed with homemade SDS-PAGE gels and nitrocellulose membranes (Merck). The following antibodies were used, mouse anti-MECP2 (Sigma-Aldrich,

M7443, 1:500), rabbit anti-RFP (Abcam, ab62431, 1:500), rabbit anti-NLuc (kind gift of Promega, 1:1000) and b-actin-peroxidase (Sigma, A3854, 1:20,000). Detection of peroxidase activity was performed using ECL western blotting detection reagent (GE Healthcare) in an Amersham Imager 600 (GE Healthcare). Detection of the remaining proteins was performed with an Odyssey CLx imaging system with Imago Studio 5.2 software (LI-COR Biosciences) with the following antibodies: IRDye 800CW donkey anti-mouse and IRDye 680RD donkey anti-rabbit (both from LI-COR Biosciences, 926-32212 and 926-68073 respectively,1:10,000).

In vitro and *in vivo* NLu Assays

NLuc assays were performed following manufacturer's instructions (Nano-Glo® Luciferase Assay System, Promega). Briefly, cells were collected and counted. A specific amount of cells (<0.5x10^6 cells) were resuspended in 25 µL PBS, and added to a well of a 96-well dish (White Cliniplate, Thermo Scientific) and 25 µL of Nano-Glo® Luciferase Assay Substrate + Buffer were added. Cells were lysed for 3 min and bioluminescence was then read in a VICTOR X4 Multilabel Plate Reader (1 s reading, 3 technical replicates, 10 s interval between readings).

P6 mice were anesthetized by isoflurane inhalation and injected intraperitoneally with furimazine (kindly provided by Promega). In short, solid furimazine was resuspended in 100% ethanol and kept for short periods of times at -80ºC. Before injections, furimazine was resuspended in 8% glycerol, 10% ethanol, 10% hydroxypropyl-β-cyclodextrin and 35% PEG400 in water, as in (Yeh et al., 2017). Anesthetized mice were injected with 5 µg furimazine/ g mouse. Briefly after injection, mice were sacrificed and their brains extracted for imaging in an IVIS Spectrum imager (PerkinElmer). Brains were imaged in an open filter with an exposure time of 5 s ($Mecp2^{+/NLucTom}$) or 5 min ($Mecp2^{+/+}$). Brain radiance (p/s/cm^2/sr) was measured from same-sized regions of interest and corrected by subtracting the background signal.

RNA-sequencing

DNA libraries from RNA samples (3 independent biological replicates) were prepared using the Smart-seq2 method and subsequently sequenced on an Illumina HiSeq2500 sequencer. The 50 bp single-end RNA-seq reads were processed allele-specifically. The SNPs

in the C57BL_6NJ and Cast/Ei lines were downloaded from the Sanger institute (v.5 SNP142)(Keane et al., 2011). These were used as input for SNPsplit v0.3.4 (Krueger and Andrews, 2016) to construct an N-masked reference genome based on mm10 in which all SNPs between C57BL_6NJ and Cast/Ei were masked. Reads were first mapped to a reference genome file containing the C57BL genome, Cast/Ei genome and the NLuc sequence using the default settings of hisat2 v2.2.1 (Kim et al., 2015). Reads that mapped to the NLuc sequence without mismatches were removed from the fastq files, after which the remaining reads were remapped to the N-masked reference genome. SNPsplit was then used to assign the reads to either the C57BL_6NJ or Cast/Ei bam file based on the best alignment or to a common bam file if mapping to a region without allele-specific SNPs. The allele-specific and unassigned bam files were sorted using samtools v1.10 (Li et al., 2009). The number of mapped reads per gene were counted for both alleles separately using HTSeq v0.12.4 (--nonunique=none -m intersection-nonempty) (Anders et al., 2015) based on the gene annotation from ensembl v98. For each sample, the number of reads that mapped perfectly to the NLuc sequence was added to the *Mecp2* gene count of the Cast/Ei allele. For each condition, genes with more than 20 allele-specific reads across the triplicates were used to calculate the allelic ratio, defined as Xi/(Xi+Xa) where the inactive X (Xi) and active X (Xa) are Cast/Ei and C57BL, respectively. The difference between the allelic ratios of X-linked genes between the High and Control samples were plotted along the X chromosome using only genes with more than 20 allele-specific reads in both conditions.

We filtered the X-linked genes based on the number of reads overlapping Xa and Xi of the control samples and the High samples separately. Active genes were selected as X-linked genes with at least 6 reads overlapping Xa of the control samples, whereas inactive genes were genes with less than 6 reads overlapping the $Xa^{Control}$. Escapee genes were selected as active genes with $Xi^{Control} \geq 5\% \ Xa^{Control}$, whereas the remainder of the genes ($Xi^{Control} < 5\% \ Xa^{Control}$) are labelled as X-inactived genes. To find the reactivated genes, we performed a differential expression analysis using DEseq2 v1.26.0 (Love et al., 2014), resulting in a list of genes with a significant allelic difference between High and Control. Reactivated genes were selected as X-inactivated genes that were also significantly differentially expressed (p-value < 0.05). For plotting, the counts of all genes were normalized using the variance stabilizing transformation (vst) function. For the plots with the low and medium conditions, DESeq2 was run on all four

conditions where the control samples were compared against the 5-Aza samples (*i.e*. low, medium and high conditions) and counts were normalized once more. We also performed differential expression analyses between Low and Control and between Medium and Control and compared the lists of significant genes to the reactivated genes in the High samples.

We compared our lists of reactivated and escapee genes to lists of genes from other studies using venn diagrams. For the comparison with (Janiszewski et al., 2019), we downloaded the allelic ratios (Mus/(Mus+Cast)) of all X-linked genes and defined genes as escapee, early, intermediate, late, very late and escapee when they were biallelically expressed (ratio between 0.15 and 0.85) at day 2, 8, 10, 13 or 15, respectively. Our list of reactivated genes was also compared to data from (Bauer et al., 2021). For each X-linked gene, we calculated the allelic ratio (Mus/(Mus+Cast)) and selected X-inactivated genes and escapees as X-linked genes based on their allelic ratio in NPCs, *i.e.* an allelic ratio < 0.14 or ≥ 0.14, respectively. The list of X-inactivated genes was divided in early and late reactivated genes based on the first day with an allelic ratio ≥ 0.14 where early was defined as genes reactivated in the samples D4 SSEA1+, D4 P-RFP+, D5 RFP+ or D6 RFP+ and late as genes reactivated in D6 X-GFPint, D6 X-GFP+, D7 X-GFP+, D7 X-GFP+, D8 X-GFP+, D9 X-GFP+ or D10 X-GFP+.

The genes from the different gene classes were compared based on several characteristics. We extracted the CpG sites from the mm10 reference genome, and counted the number of CpG sites in the region 2 Kbp upstream of the TSS to the TSS of each gene using BEDTools coverage v2.29.2 (Quinlan and Hall, 2010). Moreover, we counted the number of CpG sites overlapping the gene body of each gene and normalized by dividing this number by the gene length*0.01 to obtain the number of CpGs per 100bp of the gene body. For each gene, the distance from the TSS to the nearest escapee from our escapee list (see paragraph above) and *Xist* was identified using BEDTools closest. A table containing the locations of SINE and LINE repeat elements was downloaded from UCSC and used for calculating the number of SINEs and LINEs in the 200kb region around the TSS. We also evaluated the abundance of several SINE and LINE subtypes by plotting the number of each subtype in a 1Mb region around the TSS. LINE subtypes were organized by evolutionary age based on the youngest predicted age of the corresponding repeat masker classification in (Sookdeo et al., 2013). Finally, the number of full-length and shorter LINE elements in the 200kb region around the TSS were evaluated by

selecting LINEs <6 Kbp or LINEs ≥6 Kbp, respectively. Significant differences between gene classes were tested using a two-sided Mann-Whitney test with $\alpha < 0.05$.

To evaluate ChIP-seq enrichment around the TSS of the different gene groups, several publicly available ChIP-seq datasets from ESC-derived female neural progenitor cells were reanalysed (CTCF, H3K4me3, H3K27me3 and RAD21 from GSE99991). In short, reads were mapped to the N-masked reference genome generated by SNPsplit based on the SNPs between the C57BL_6NJ and Cast/Ei genomes using bowtie2 v2.4.1 (Kim et al., 2015; Langmead and Salzberg, 2012). SNPsplit was then used to assign the reads to either the C57BL_6NJ or Cast/Ei bam file based on the best alignment or to a common bam file if mapping to a region without allele-specific SNPs. The allele-specific and unassigned bam files were sorted using samtools v1.10 (Li et al., 2009). Finally, the allele-specific bam files were normalized based on the total number of mapped reads per sample. The scaling factor was calculated as 10^6 / total number of mapped reads and used as parameter --scaleFactor using deepTools bamCoverage v3.5.0 (--extendReads --binSize 1). Replicates were merged using using WiggleTools v1.2.3 (Zerbino et al., 2014). Xist CHART-seq data from (Wang et al., 2018) was downloaded and lift over from mm9 to mm10 using CrossMap v0.5.2 (Zhao et al., 2014). Allele-specific ChIP-seq density ± 3kb around the TSS of the different gene groups was visualized using deepTools plotProfile v3.5.0 (Ramírez et al., 2016). Moreover, the overlap of the CHART-seq data with the gene body was plotted using deepTools plotProfile showing 3kb upstream of the TSS to 3kb downstream of the TES.Hi-C data from female NPCs was downloaded from (Bauer et al., 2021) (GSE157448). The allele-specific Hi-C matrices were corrected using HiCExplorer v3.6 (Ramírez et al., 2018) hicCorrectMatrix with ICE as correction method and a lower and upper threshold of -1.4 and 2, respectively. The Xi and Xa Hi-C data was visualized using pyGenomeTracks (Lopez-Delisle et al., 2020). The TAD boundaries were downloaded from Bonev et al 2017. For each TAD on the X-chromosome, the number of overlapping genes, reactivated genes and escapees were counted using bedtools intersect. TADs with significant more or less reactivated genes were selected using a Binomial test based on the ratio between the number of reactivated genes and the total number of genes for the whole X-chromosome (p-value < 0.05). The tracks showing the SINE, LINE and CpG density, the CHART-seq track and the allele-specific tracks from CTCF, RAD21, H3K4me3 and H3K27me3 were added. Differences between TADs were plotted in violin plots by

comparing the TADs with significantly more reactivated genes, significantly less reactivated genes and non-significant TADs. For each TAD, the number of overlapping CpG sites, SINEs and LINEs were counted using bedtools intersect and normalized for the TAD length. Moreover, the number of overlapping reads from the CHART-seq and allele-specific ChIP-seq data was counted using bedtools intersect and normalized for the TAD length.

MeD-seq

MeD-seq analyses were essentially carried out as previously described (Boers et al., 2018). In brief: DNA samples were digested by *LpnPI* (New England Biolabs, Ipswich, MA, USA), resulting in snippets of 32 bp around a fully-methylated recognition site that contains a CpG. These short DNA fragments were further processed using a ThruPlex DNA–seq 96D kit (cat#R400407, Rubicon Genomics Ann Arbor, MI, USA) and a Pippin system. Stem-loop adapters were blunt-end ligated to repaired input DNA and amplified to include dual indexed barcodes using a high-fidelity polymerase to generate an indexed Illumina NGS library. The amplified end product was purified on a Pippin HT system with 3% agarose gel cassettes (Sage Science, Beverly, MA, USA). Multiplexed samples were sequenced on Illumina HiSeq2500 systems for single reads of 50 bp according to the manufacturer's instructions. Dual indexed samples were demultiplexed using bcl2fastq software (Illumina, San Diego, CA, USA). Data processing was carried out using custom scripts in Python. Raw fastq files were subjected to Illumina adaptor trimming and reads were filtered based on *LpnPI* restriction site occurrence between 13-17 bp from either 5' or 3' end of the read. Reads that passed the filter were mapped to mm10 using. For each *LpnPI* site, the number of overlapping reads were counted and normalized for the sequencing depth. We defined the TSS region as the region ± 1kb of the TSS and generated read count scores for the TSS region of each gene. Differentially methylated TSS regions were detected using a Mann-Whitney test on the normalized read counts of the High samples and the control samples.

For each gene, the ratio between high and control was calculated by dividing the normalized number of reads overlapping the TSS region in the high samples by the those overlapping the TSS region in the control samples. Only genes with more than 10 reads overlapping the TSS region across all samples were used. The ratios between the genes of the

different gene classes were compared using a violin plot showing the ratios per group. Methylation differences between the TSS region of reactivated and non-reactivated genes were explored by plotting the methylation profiles for both genes in a heatmap. For each gene, the normalized number of reads overlapping the TSS region were converted to z-scores for plotting. The genes were clustered based on the Euclidean distance and annotated as either reactivated or non-reactivated to reveal clustering differences between both groups.

Demethylation of SINEs and LINEs was examined by selecting SINEs and LINEs in the 200kb region around the TSSs of reactivated and non-reactivated genes. We counted the number of MeD-seq reads in the 400bp region around the SINEs and LINEs and normalized this number for sequencing depth using the total number of mapped MeD-seq reads per sample. For each repeat element, the ratio between high and control was calculated by dividing the normalized number of reads in the high samples by the those in the control samples. The methylation ratios of the SINEs and LINEs close to genes from the different gene classes were visualized in violin plots.

For the genome browser overviews of the MeD-seq samples, the bam files were normalized using deepTools bamCoverage v3.5.0 (Ramírez et al., 2016) with CPM as normalization method and a bin size of 1. For each condition, the tracks of the replicates were merged using WiggleTools v1.2.3 (Zerbino et al. 2014).

Harmony image analysis

Neurons were processed as per the IF protocol described above. Images were acquired with an Opera Phenix confocal microscope (PerkinElmer) and analyzed with a Harmony software (v4.9, PerkinElmer). Nuclei were determined with DAPI while TUJ-1-Alexa488-positive cells were selected and further analyzed for nuclear area, number of extremities per nucleus and number of roots per nucleus.

Supplemental References

Anders, S., Pyl, P.T., and Huber, W. (2015). HTSeq—a Python framework to work with high-throughput sequencing data. Bioinformatics *31*, 166–169.

Bauer, M., Vidal, E., Zorita, E., Üresin, N., Pinter, S.F., Filion, G.J., and Payer, B. (2021). Chromosome compartments on the inactive X guide TAD formation independently of transcription during X-reactivation. Nat Commun *12*, 3499.

Boers, R., Boers, J., Hoon, B. de, Kockx, C., Ozgur, Z., Molijn, A., IJcken, W. van, Laven, J., and Gribnau, J. (2018). Genome-wide DNA methylation profiling using the methylation-dependent restriction enzyme LpnPI. Genome Res *28*, 88–99.

Carrette, L.L.G., Wang, C.-Y., Wei, C., Press, W., Ma, W., Kelleher, R.J., and Lee, J.T. (2018). A mixed modality approach towards Xi reactivation for Rett syndrome and other X-linked disorders. Proceedings of the National Academy of Sciences of the United States of America *115*, E668–E675.

Csankovszki, G., Panning, B., Bates, B., Pehrson, J.R., and Jaenisch, R. (1999). Conditional deletion of Xist disrupts histone macroH2A localization but not maintenance of X inactivation. Nature Genetics *22*, 323–324.

Guy, J., Hendrich, B., Holmes, M., Martin, J.E., and Bird, A. (2001). A mouse Mecp2-null mutation causes neurological symptoms that mimic Rett syndrome. Nature Genetics *27*, 322–326.

Janiszewski, A., Talon, I., Chappell, J., Collombet, S., Song, J., Geest, N.D., To, S.K., Bervoets, G., Marin-Bejar, O., Provenzano, C., et al. (2019). Dynamic reversal of random X-Chromosome inactivation during iPSC reprogramming. Genome Research *29*, 1659–1672.

Keane, T.M., Goodstadt, L., Danecek, P., White, M.A., Wong, K., Yalcin, B., Heger, A., Agam, A., Slater, G., Goodson, M., et al. (2011). Mouse genomic variation and its effect on phenotypes and gene regulation. *477*, 289–294.

Kim, D., Langmead, B., and Salzberg, S.L. (2015). HISAT: a fast spliced aligner with low memory requirements. Nat Methods *12*, 357–360.

Krueger, F., and Andrews, S.R. (2016). SNPsplit: Allele-specific splitting of alignments between genomes with known SNP genotypes. F1000research *5*, 1479.

Langmead, B., and Salzberg, S.L. (2012). Fast gapped-read alignment with Bowtie 2. Nat Methods *9*, 357–359.

Li, H., Handsaker, B., Wysoker, A., Fennell, T., Ruan, J., Homer, N., Marth, G., Abecasis, G., Durbin, R., and Subgroup, 1000 Genome Project Data Processing (2009). The Sequence Alignment/Map format and SAMtools. Bioinformatics *25*, 2078–2079.

Lopez-Delisle, L., Rabbani, L., Wolff, J., Bhardwaj, V., Backofen, R., Grüning, B., Ramírez, F., and Manke, T. (2020). pyGenomeTracks: reproducible plots for multivariate genomic data sets. Bioinformatics *37*, btaa692-.

Love, M.I., Huber, W., and Anders, S. (2014). Moderated estimation of fold change and dispersion for RNA-seq data with DESeq2. Genome Biol *15*, 550.

Quinlan, A.R., and Hall, I.M. (2010). BEDTools: a flexible suite of utilities for comparing genomic features. Bioinformatics *26*, 841–842.

Ramírez, F., Ryan, D.P., Grüning, B., Bhardwaj, V., Kilpert, F., Richter, A.S., Heyne, S., Dündar, F., and Manke, T. (2016). deepTools2: a next generation web server for deep-sequencing data analysis. Nucleic Acids Res *44*, W160–W165.

Ramírez, F., Bhardwaj, V., Arrigoni, L., Lam, K.C., Grüning, B.A., Villaveces, J., Habermann, B., Akhtar, A., and Manke, T. (2018). High-resolution TADs reveal DNA sequences underlying genome organization in flies. Nat Commun *9*, 189.

Sookdeo, A., Hepp, C.M., McClure, M.A., and Boissinot, S. (2013). Revisiting the evolution of mouse LINE-1 in the genomic era. Mobile Dna-Uk *4*, 3–3.

Spiliotopoulos, D., Goffredo, D., Conti, L., Febo, F.D., Biella, G., Toselli, M., and Cattaneo, E. (2009). An optimized experimental strategy for efficient conversion of embryonic stem (ES)-derived mouse neural stem (NS) cells into a nearly homogeneous mature neuronal population. Neurobiol Dis *34*, 320–331.

Wang, C.-Y., Jégu, T., Chu, H.-P., Oh, H.J., and Lee, J.T. (2018). SMCHD1 Merges Chromosome Compartments and Assists Formation of Super-Structures on the Inactive X. Cell *174*, 406-421.e25.

Yeh, H.-W., Karmach, O., Ji, A., Carter, D., Martins-Green, M.M., and Ai, H. (2017). Red-shifted luciferase–luciferin pairs for enhanced bioluminescence imaging. Nat Methods *14*, 971–974.

Zerbino, D.R., Johnson, N., Juettemann, T., Wilder, S.P., and Flicek, P. (2014). WiggleTools: parallel processing of large collections of genome-wide datasets for visualization and statistical analysis. Bioinformatics *30*, 1008–1009.

Zhao, H., Sun, Z., Wang, J., Huang, H., Kocher, J.-P., and Wang, L. (2014). CrossMap: a versatile tool for coordinate conversion between genome assemblies. Bioinformatics *30*, 1006–1007.
